# Temporal dynamics of whole body residues of the neonicotinoid insecticide imidacloprid in live or dead honeybees

**DOI:** 10.1038/s41598-017-06259-z

**Published:** 2017-07-24

**Authors:** Matthias Schott, Gabriela Bischoff, Gerrit Eichner, Andreas Vilcinskas, Ralph Büchler, Marina Doris Meixner, Annely Brandt

**Affiliations:** 1 Institute for Zoology, University of Cologne, Cologne, Germany; 2 Institute for Insectbiotechnology, Justus-Liebig University of Giessen, Giessen, Germany; 3 Julius Kühn-Institut - Federal Research Centre for Cultivated Plants, Institute for Bee Protection, Berlin, Germany; 4 Mathematical Institute, Justus-Liebig University of Giessen, Giessen, Germany; 5 Fraunhofer Institute for Molecular Biology and Applied Ecology, Department of Bioresources, Giessen, Germany; 6 LLH Bee Institute, Erlenstr. 9, 35274 Kirchhain, Germany

## Abstract

In cases of acute intoxication, honeybees often lay in front of their hives for several days, exposed to sunlight and weather, before a beekeeper can take a sample. Beekeepers send samples to analytical laboratories, but sometimes no residues can be detected. Temperature and sun light could influence the decrease of pesticides in bee samples and thereby residues left for analysis. Moreover, samples are usually sent via normal postal services without cooling. We investigated the temporal dynamics of whole-body residues of imidacloprid in live or dead honeybees following a single-meal dietary exposure of 41 ng/bee under various environmental conditions, such as freezing, exposure to UV light or transfer of individuals through the mail system. Immobile, “dead” looking honeybees recovered from paralysis after 48 hours. The decrease of residues in living but paralysed bees was stopped by freezing (= killing). UV light significantly reduced residues, but the mode of transport did not affect residue levels. Group feeding increased the variance of residues, which is relevant for acute oral toxicity tests. In conclusion, elapsed time after poisoning is key for detection of neonicotinoids. Freezing before mailing significantly reduced the decrease of imidacloprid residues and may increase the accuracy of laboratory analysis for pesticides.

## Introduction

Honeybees (*Apis mellifera L*.) and wild bees are primary pollinators of wild plants and cultivated crops and therefore essential for ecosystem function and agriculture^1–3^. Over the past decade there has been a serious decline in bee populations reported in parts of the world, including the European Union (EU)^4–9^. A multitude of causative factors is being discussed, such as parasites and pathogens, diet quantity, quality, diversity, and the exposure to pesticides^9–15^. In particular the application of neonicotinoid insecticides has been suspected to represent a major threat to honeybee survival^16–21^. Especially three neonicotinoids, frequently used as seed dressings (imidacloprid, thiamethoxam, and clothianidin), are discussed in the context of bee declines, which led to a moratorium of these substances in the EU^22^.

Neonicotinoids are neurotoxins that act as agonists of the nicotinic acetylcholine receptor. The disruption of the neuronal cholinergic signal transduction causes abnormal behavior, immobility, and death of target insect pests^23–25^. Frequently, also non-target insects like honeybees are exposed to these insecticides^12, 19, 26–28^, since forager bees transport neonicotinoid-contaminated pollen, nectar, dust, or guttation fluids to their colonies, where they can be frequently detected in honey and bee bread^12, 13, 29–31^. Imidacloprid, one of the most commonly used insecticides^32^, is highly toxic to honeybees and other beneficial insects such as wild bees^12, 20, 33^. Frequently, honeybees are exposed to lower concentrations of neonicotinoids, leading to sublethal effects, like impaired learning or homing behaviour, or suppressed immune defence^34–37^. Whether sublethal effects of neonicotinoids are reversible or even low-level dietary residues can accumulate to be lethal is controversial^38–43^.

Direct lethal effects of neonicotinoids occur only rarely, mostly caused by accidental exposure of forager bees to acute toxic concentrations of neonicotinoids^44^. In such cases, when a high number of bees are found in front of the hives, beekeepers can send a sample of bees for residue analysis to a laboratory to test for pesticide residues, including neonicotinoids and their metabolites. In Germany, the recommended practice is to send the samples via standard postal routes without extra cooling. During transportation, samples can be exposed to fluctuations in temperature and humidity, depending on climate, season, and weather, which could influence the decrease of imidacloprid, and thereby the level of residues left for analysis upon arrival at the analytical laboratory.

In this study, we investigated the temporal dynamics of whole-body residues of imidacloprid in live or dead honeybees following a single-meal dietary exposure (acute dose paradigm) under various environmental conditions, such as freezing of individuals, exposure to UV light or transfer of individuals through the mail system. Under field conditions, it is often not clear whether immobile bees found in front of the hives are really dead or only have been paralysed by the pesticides. Therefore, we wanted to test, (1) if rapidly freezing (= killing) of the bees can prevent or slow down the decrease of imidacloprid residues. Beekeepers do not check on their colonies on a daily basis, thus intoxicated honeybees may lie in front of their hives for several days, exposed to sun and weather. Since neonicotinoids are known to be sensitive to photolytic degradation^45^ by sunlight, we wanted to test (2), whether exposure to UV light might affect the decrease of whole-body residues in honeybee samples. (3) To test if the mode of transportation affects the decrease of imidacloprid, we examined whether rapid transportation of dead bees on dry ice can preserve the level of residues left for analysis. According to the OECD guideline^46^, oral toxicity tests are conducted in cages with groups of ten bees exposed to test substances. To examine whether the group feeding influences the residue level in honeybee, (4) we compared imidacloprid residues in single fed bees compared to bees that were fed in groups.

## Results

To study the dynamic decrease of whole-body imidacloprid residues in live or dead honeybees, we individually fed worker bees with a single dose of 41 ng imidacloprid. Some bees were exposed to intensive UV light (RT24UV). Subsequently, after one (RT1, RT1mail), 24 (RT24, RT24UV), and 48 (RT48) hours after the feeding, the bees were frozen. The samples (n = 150 individuals per treatment group) were either transported at ambient temperature via normal postal mail service (RT1mail) or on dry ice via express service to the analytical laboratory (all other samples).

Immediately after the intake of the imidacloprid spiked feeding solution (41 ng/bee), honeybees showed spasms and appeared immobile and lifeless in the centrifuge tubes one hour after the feeding took place. However, bees that were kept at room temperature after dosing (RT48 treatment) recovered and walked up and down the tubes 48 hours after ingesting treated syrup.

### Impact of time at room temperature

**Figure 1:**
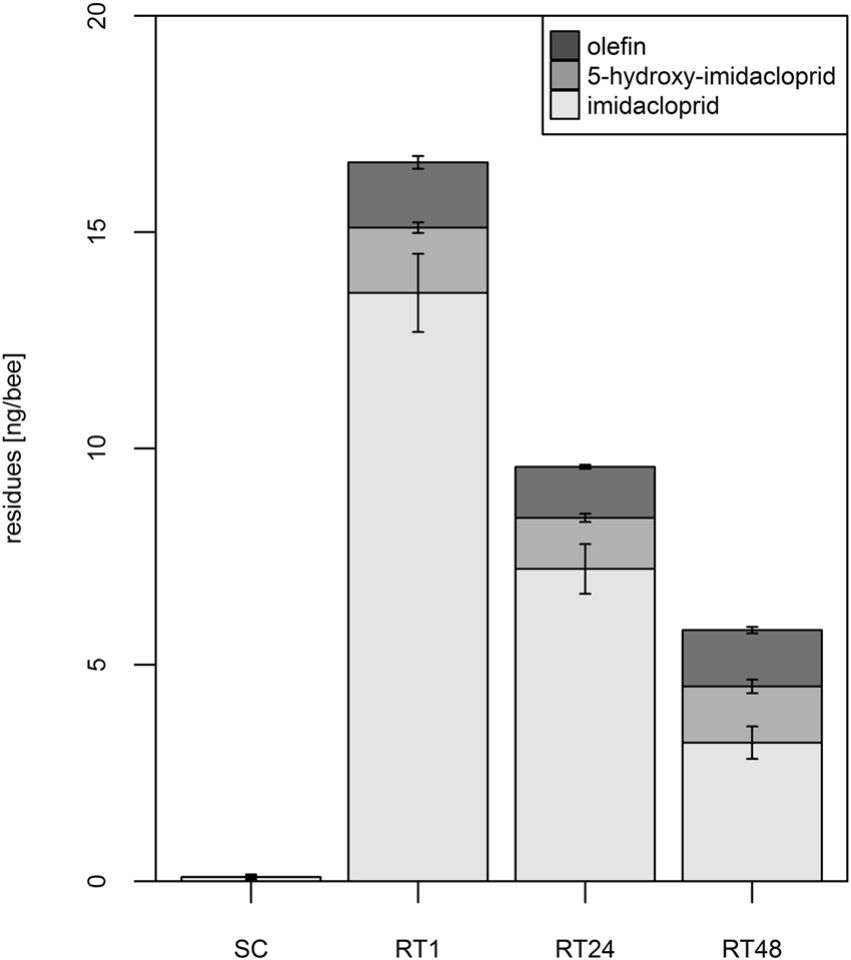
Whole-body imidacloprid residues decrease in honeybee samples over time. Individual honeybees were fed with a sugar syrup control (SC), containing only the solvent acetone but no imidacloprid or a dosage of 41 ng/bee imidacloprid (n = 150 bees/group). The honeybees were frozen after one hour (SC and RT1), 24 hours (RT24), or 48 hours (RT48) at room temperature. LC/MS/MS was used to quantify residue levels of imidacloprid, 5-hydroxy-imidacloprid, and imidacloprid-olefin. Error bars indicate standard error. The imidacloprid residue level was only significantly influenced (p < 0.001) by the time passed after ingestion. The residue level of 5-hydroxy-imidacloprid was influenced by the hive the bees originated from, the time passed after ingestion and interaction effects between both (all p < 0.001). The residue level of imidacloprid-olefin was only influenced by the interaction effects between the hive the bees originated and the time passed after ingestion (p < 0.001, see statistical report in supplement).

Under field conditions, it is often not clear, whether immobile bees are dead or only paralysed by a toxin. Hence, active cellular detoxification may take place even in “dead” looking bees. Therefore, we wanted to test, whether killing the bees by freezing can slow down or stop the decrease of imidacloprid residues. Indeed, the period of time elapsed before freezing significantly affected the amount of imidacloprid detected in the samples (Fig. 1). The regression analysis yielded a significant time main effect with a half-life of imidacloprid of about 22 hours (with a 95%-confidence interval from 18.6 to 26.8 hours, see statistical report in supplement). The hive experimental bees originated from did not show a significant influence in the regression model.

We detected 33.2 ± 2.2% (mean ± relative standard error) of the ingested imidacloprid dose in the RT1 sample, 17.6 ± 1.4% in the RT24 sample and 7.8 ± 0.9% in the RT48 sample. The degradation process and recovery rate were investigated in more detail by quantifying the imidacloprid metabolites 5-hydroxy-imidacloprid and imidacloprid-olefin (Fig. 1). For 5-hydroxy-imidacloprid residue levels we observed a significant main effect of the hive the bees originated from as well as a significant main effect of time (elapsed after ingestion), and a significant interaction between hive and time (see statistical report in supplement). The levels of Imidacloprid-olefin were similar in RT1, RT24 and RT48 samples. Hive and time before freezing had no significant impact on olefin residues, but the interaction between hive and time had a significant impact on the detectable Imidacloprid-olefin amount (see statistical report in supplement).

### Impact of transport method

To test whether the transport of the samples to the analytical laboratory influences imidacloprid residue levels, bees were individually exposed to imidacloprid and frozen one hour after the feeding. All bee samples were frozen at −20 °C. One treatment group (RT1mail) was only frozen for one hour at −20 °C, but subsequently stored at 4 °C as it is recommended practice for beekeepers to store bee samples in the refrigerator. Later, the bees of the treatment group RT1mail were sent for analysis using a standard parcel delivery service at ambient temperatures. For these samples, we chose a selection of dispatch dates, including the days before weekends and bank holidays, so the delivery took between one and six days. All other samples were sent by express delivery on dry ice and arrived on the same day at the analytical laboratory.

**Figure 2:**
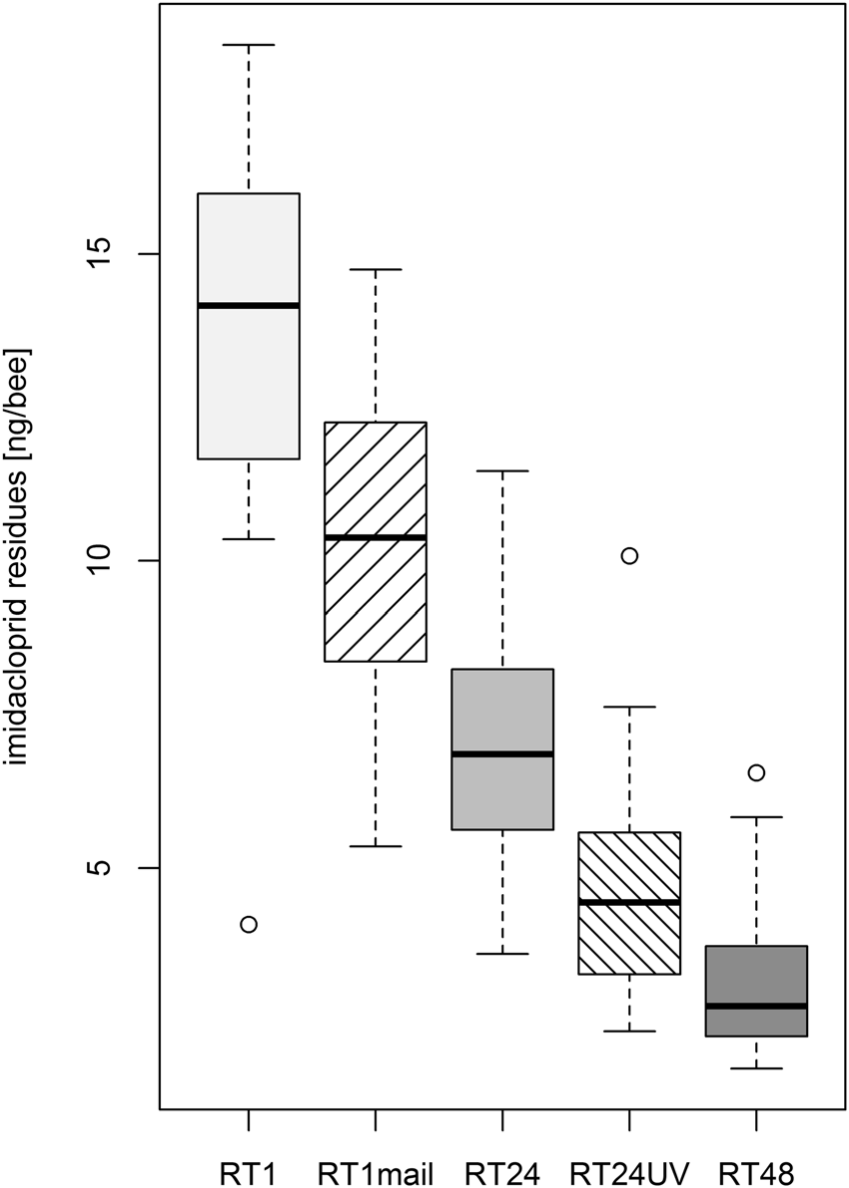
UV light accelerated the decrease of whole-body imidacloprid residues in honeybees, transportation on ice had no significant effect. Individual honeybees were fed with a dosage of 41 ng/bee. The honeybees were frozen after one hour (RT1, RT1mail), 24 hours (RT24, RT24UV), or 48 hours at room temperature. The samples of RT1, RT24, RT24UV, and RT48 were sent on dry ice with express delivery, RT1mail samples were sent without cooling via standard parcel service to the analytical laboratory (n = 150 bees/group). The mode of transport did not significantly affect imidacloprid residue levels (Comparison RT1 vs. RT1mail: p = 0.093). Intensive UV-light (RT24UV) significantly accelerated the decrease of imidacloprid (comparison RT24 vs. RT24UV: p = 0.0163; RT48 vs. RT24UV: p = 0.1637, see statistical report in supplement).

The transport at ambient temperature via standard postal service did not significantly reduce the amount of imidacloprid compared to samples sent on dry ice (Fig. 2; RT1 vs. RT1mail, multiple comparisons using Dunnett contrasts, p = 0.093, see statistical report in supplement), although the samples were exposed to ambient temperature over long periods, with a maximum shipping duration of 144 hours. The residues in the RT24 and RT48 samples were significantly lower (p = 0.0218 and p < 0.001, respectively) than those of the RT1mail group. The hives the bees originated from and the interaction between hive and treatment (mailing) had no significant effect on the detectable imidacloprid values.

### Impact of UV radiation

Exposure to UV light significantly reduced the imidacloprid concentration (p = 0.0163 comparing RT24 and RT24UV using Dunnett contrasts, Fig. 2). The concentration in RT24UV was not significantly different from the one observed in RT48.

### Impact of feeding condition

**Figure 3:**
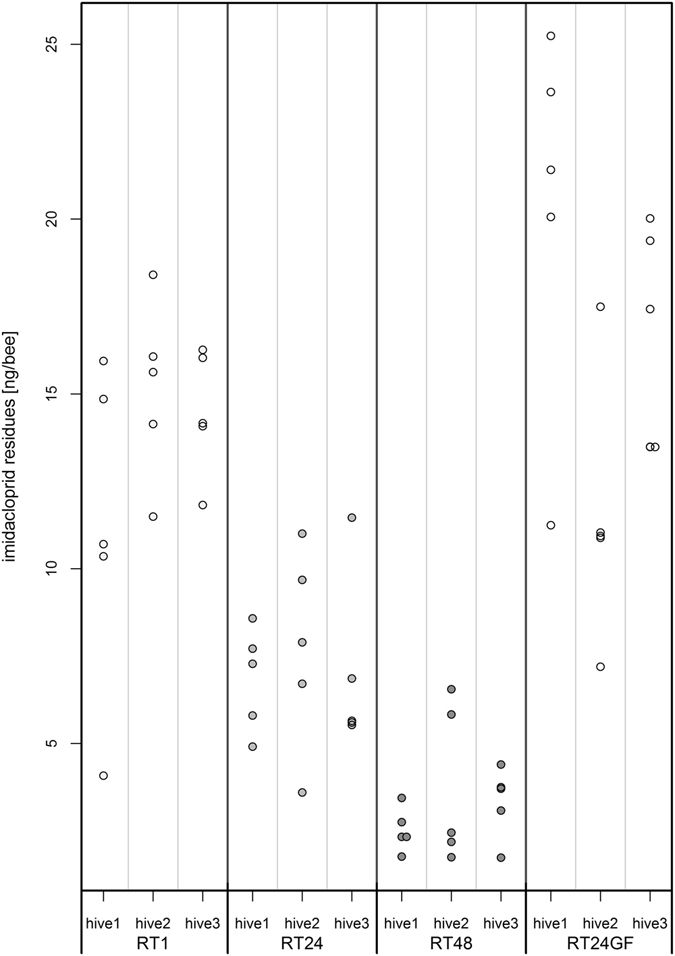
Group feeding resulted in significantly higher whole-body imidacloprid residues compared to the individually fed honeybees. Individual honeybees were fed with a single dosage of 41 ng/bee (RT1, RT24, RT48). Alternatively, groups of ten honeybees per cage were fed with 410 ng/cage imidacloprid (RT24GF). One dot indicates imidacloprid value of one sample of ten bees. Five samples of each hive are presented in one column (n = 150 bees/group). Statistical comparison with an ANOVA yielded a significant effect of the applied treatment (p < 0.001) and multiple comparisons using Dunnett contrasts resulted in RT1 vs. RT24GF: p = 0.0128; RT24 vs. RT 24GF: p < 0.001; RT48 vs. RT24GF: p < 0.001 (see statistical report in supplement).

Compared to individually fed bees, bees that were fed in groups of ten had significantly higher imidacloprid residues (Fig. 3, RT1 vs. RT24GF, Dunnett contrasts, p = 0.0128; RT24 vs. RT24GF, Dunnett contrasts, p < 0.001; RT48 vs. RT24GF, Dunnett contrasts, p < 0.001, see statistical report in supplement). We assessed the “quality” of the obtained data using the Bartlett-, the Fligner-Killeen- and the F-test to compare variances of the residue levels. Only if we neglected the hive the bees originated from, variances of RT24 and RT48 were significantly different from the variance in RT24GF.

### Dose-dependent effects on locomotory behavior

**Figure 4:**
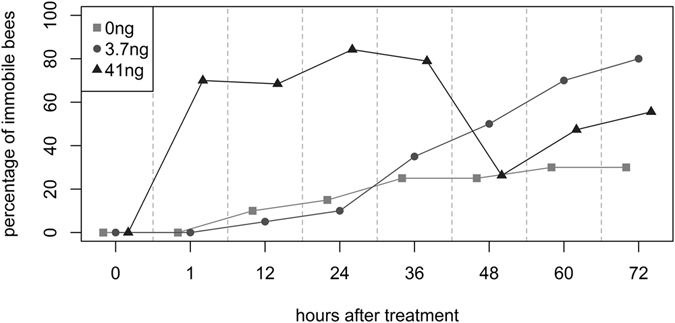
Imidacloprid-exposed bees partly recovered from paralysis after 48 hours. Individual honeybees were fed with a single dose of sugar syrup control or a dosage of 3.7 and 41 ng/bee imidacloprid (20 bees per treatment group, single experiment). After one, 12, 24, 36, 48, 60, and 72 hours bees were examined for activity. Non-moving bees were counted as immobile. After one hour, 70% of the bees of the 41 ng treatment were immobile, but recovered to the same level of immobility as the control after 48 hours.

In order to assess the effect of different dosages of imidacloprid on the bee’s mobility, individual bees were exposed to different imidacloprid dosages and observed for 72 hours. Bees of the control group showed a decline in activity over the observation period (Fig. 4 and Supplementary Fig. 1). Bees fed with a dosage of 3.7 ng (corresponding to LD50_oral 48h_^47^, showed normal activity until 24 hours after exposure. At 36 hours, a steep decline in activity began. At 72 hours, most bees (80%) were completely immobile. In contrast, the bees fed with a dosage of 41 ng (corresponding to the dosage used in the experiments described above), showed cramps, uncoordinated movements, or paralysis one hour after exposure. At 36 hours most bees (78.9%) were completely immobile. Strikingly, after 48 hours only 26.3% of the bees were immobile, 47.3% of the bees exhibited some movement of body parts and 26.3% showed coordinated movements, e.g. walking or ventilation (Supplementary Figure 1). After this peak, activity declined again at 72 hours. In consequence to the uncoordinated movements most bees were not able to feed properly (pers. observation) and the experiment was stopped.

## Discussion

Here, we provide data on the decrease of whole-body residues of imidacloprid in acutely poisoned bees and explore the effects of various environmental conditions, such as freezing of individuals, exposure to UV light or routes of shipping on the whole-body residue levels measured. The results allow recommending a standard sampling and shipping procedure to beekeepers in cases of suspected acute poisoning with imidacloprid. Thus, they contribute to a decrease of uncertainty in interpretation of analysis results and eventually to strengthen the confidence of beekeepers in extension and communication services.

To study the temporal dynamics of the decrease of whole-body residues that takes place in imidacloprid exposed bees, we analysed the residue levels in individual bees one, 24, and 48 hours after exposure. We used a high dosage of imidacloprid (41 ng/bee) to ensure that a measurable level of imidacloprid was left for detection after 48 hours. In the assessment of risks from imidacloprid to bees, the EFSA considered the median lethal dose (LD50_48h_) at 3.7 ng/bee^47^. However, reports of the oral acute LD50_48h_ range up to >81 ng^48^. Surprisingly, a decrease of whole-body residues took place even in motionless, “dead” looking bees. Supporting these findings, poisoned, motionless bees partially recovered after approx. 48 hours post administration of 41 ng imidacloprid. The bees in our study became active again, when imidacloprid was degraded to a level of approx. 4 ng/bee.

Neonicotinoids act as neurotoxins. Whether the interactions of imidacloprid with its target site in the nervous system – the nicotinic acetylcholine receptor - is reversible or not is an academic controversy which has practical implications for the risk assessment of neonicotinoids^38–43^. In case bees would fail to fully clear ingested pesticides from their bodies, the persistence of even small daily intakes could eventually build up to harmful or even lethal levels over time. Some scientists argue that imidacloprid irreversibly blocks the nicotinic acetylcholine receptors^40–42^. Indeed, the lethality of imidacloprid to insects appears to be dependent on the time of exposure: the longer the exposure time, the less amount of total chemical is needed to kill honeybees^40, 49, 50^. However, imidacloprid and its metabolites are rapidly metabolised by detoxification enzymes in bees and a partial or full recovery from imidacloprid-induced sublethal effects on bees has been observed^38, 51, 52^. In support of the reversibility-hypothesis, our data clearly indicate, that a recovery from the paralysing neurotoxic effects of a high dose of imidacloprid is possible. Strikingly, we found a non-linear dose-response relationship: the lower dose (LD50_48h_ 3.7 ng/bee) had a greater impact on bee immobility/death than the higher dose of 41 ng (48 hours after ingestion). Apparently, lethal effects are delayed in bees paralysed by a high dose of imidacloprid. Non-linear dose-response relationships of toxins have been reported before^53, 54^. This raises the question, what the ultimate physiological cause of death by neonicotinoids is in honeybees. Further studies are needed to elucidate this relevant question.

In small invertebrates it is hard to measure brain waves, heartbeat, or respiration; hence it is difficult to define the exact moment of death. The OECD guideline defines mortality/death in honeybees as when an animal is “completely immobile”^46^. This is an important criterion, since in acute oral toxicity tests required for regulatory risk assessment procedures, the LD50 is defined as a single dose of a substance that can cause death (= immobility) in 50% of animals^46^. The possibility that honeybees can recover from an immobile state has not been considered before in the OECD guideline. Based on our observation of honeybees recovering from a completely immobile state, the definition of “mortality” by the OECD may need to be reconsidered. Up to now, immobile, “dead” looking bees have to be removed daily and are usually discarded; hence there was no chance to observe a potential recovery form paralysing toxins. In field realistic conditions however, this question is less relevant since most foragers which come into contact with extremely toxic, paralysing substances may become easy prey and are lost to the colony.

In cases of suspected acute poisoning, beekeepers can send honeybee samples to certified laboratories for residue analysis. Sometimes, no residues can be detected in such samples, but it is often unclear whether no substance was present from the beginning or whether contaminant residue levels decreased in the time that passed since the poisoning event or during shipping. Beekeepers usually do not inspect their bee yards daily, thus poisoned honeybees may lay in front of their hives for several days before a sample can be taken. Consequently, the chances of finding any dead bees decreases with time, since immobilised bees may become easy prey for birds or ants.

From controlled laboratory studies it is known that whole-body imidacloprid residues can rapidly decrease^38, 51, 52, 55^. Hardly any data exist, however, on the breakdown of the substance under conditions that may apply to a field situation where bees are acutely poisoned and become immobilised (and are taken for dead), or in bee samples during shipping to analytic facilities. Thus, a reliable interpretation of residue analyses results is often difficult and may be hampered by substantial uncertainty. After a single feeding of imidacloprid, honeybees eliminate the neonicotinoid from their body within 24 hours mostly through metabolic degradation rather than by excretion of the compound^51, 52^. Our data confirm the rapid decrease of whole-body imidacloprid residues in honeybees^38, 51, 52^. Even though we used a relatively high dose of imidacloprid, only small amounts of the two imidacloprid metabolites tested, imidacloprid-olefin and 5-hydroxy-imidacloprid, were detectable after 48 hours. However, for a complete picture of the dynamics of the decrease of imidacloprid residues in honeybee samples, it would be necessary to quantify all known metabolites^20, 33^.

While the decrease of whole-body imidacloprid residues continuously progressed, even in bees that appeared dead, freezing the bees for a short time significantly slowed down this process. In cases where immobilised bees are found in an apiary and the beekeeper suspects poisoning by neonicotinoids, we therefore recommend instantaneous short freezing of the samples to stop possible cellular detoxification processes. Provided the decrease of whole-body residues has been slowed or stopped by freezing, our data show that no further significant decrease of imidacloprid occurs during standard shipping, compared to the transport on dry ice. Thus, the mode of transport does not affect the residue levels of imidacloprid in honeybee samples, and beekeepers can continue to send their samples without cooling via standard postal routes.

In the field, poisoned honeybees laying in front of their hive may eventually be exposed to sunlight which could further contribute to the decrease of detectable residues. A previous study demonstrates that imidacloprid is photodegradable by UV-irradiation^45^, however, the data were generated using *in vitro* assays and did not involve live organisms or bees. In live bees or dead bees, the cuticle or the melanisation of the body may provide protection against UV radiation and may prevent photolytic degradation processes. In fact, in our experiment a moderately accelerated decrease of whole-body imidacloprid residues was observed after intensive UV irradiation. However, although the applied amount of UV radiation corresponded to the amount of UV light during two cloudless weeks at the equator, its effect on the residue degradation was less pronounced than that of an additional 24 hours of room temperature (see RT24UV and RT48, Fig. 2). In consequence, we consider photolytic degradation a less important factor affecting the detectable level of imidacloprid residues in honeybee samples.

In accordance with regulatory guidelines for pesticide risk assessments, acute oral toxicity tests are conducted with caged bees, where groups of ten bees per cage are exposed to the test compound^46^. When applying imidacloprid under group feeding conditions, we observed a high variance and a significantly higher amount of residue level compared to individually fed bees. Our data suggest that some individuals may have consumed more than others and therefore the bees in a cage may not have been equally exposed to the test substance, confirming observations from Brodschneider *et al*.^56^ who demonstrated by radioactively labelled markers that differences in food intake exist within caged bees that eventually may lead to differential exposure in toxicity tests. Since a worker bee which consumes the spiked sugar syrup becomes immobile after a few minutes, it has only little chances to share the food by trophallaxis with its cage mates. Thus, maybe only few individuals of a cage consume most of the contaminated food and others receive less. In this case, the variance in the recovery rates between the cages may result from the variable number of individuals which had direct contact to the syrup. The individuals that ate most of the contaminated food could not have been able to detoxify as fast as the single fed bees that really received the planned concentrations per individual. In contrast to the OECD guidelines^46^, where a minimum of 100 µl sugar syrup for one cage is proposed, we only gave them 50 µl of the feeding solution. Thus, in an experiment performed exactly conforming to the guideline, the variance between cages might be lower, since more bees would be in direct contact to the test substance. However, our findings of the impact of feeding condition are not only relevant for immediately paralyzing test substances like imidacloprid, but for all substances (e.g. neurotoxins), which might interfere with the trophalactic exchange of food. We should therefore consider whether social insects like honeybees should always be monitored at group level. This may depend on the aim of the study. Under field conditions intoxications occur first on an individual level. Trophallaxis will only occur later if the bees are able to fly back to the hive. Group feeding does not account for the estimation of the detoxification process in full hive situation. The processes there are more complex and can hence be only evaluated by full hive studies.

## Conclusion

The metabolism and toxicokinetics of imidacloprid has been intensively investigated^33, 38, 51, 52, 55, 57^. Our data complement the knowledge of the temporal dynamics imidaclopid residues under field relevant conditions. Our study indicates that a short freezing of bee samples stops the decrease of whole-body residues and further shipping time does not change the amount of traceable residues. Importantly, when bees appear immobile or dead a fast decrease of the ingested pesticides could still occur. Therefore, we recommend freezing honeybee samples as fast as possible, before sending them via standard postal routes to the analytical laboratories. However, honeybees come in to contact with many different chemical compounds used in agriculture. In beebread-samples regularly more than 70 different active compounds can be found (on average 5.2 active compounds per hive), some of them highly toxic for insects^29, 58^. In cases of suspected honeybee poisoning a high number of pesticides are analyzed to identify and quantify the relevant substance. Yet, about the metabolism, degradation rates and possible synergistic interactions^59^ of the majority of these compounds only very little is known. Our work gives new insights into an important aspect of residue analysis in honeybees and contributes to the ongoing discussion among scientist, beekeepers, and stakeholders about the impact of pesticides on honeybee health and survival.

## Experimental Procedures

### Specimens

Honeybee worker bees were collected from brood combs of a colony of Carnolian bees located at the Fraunhofer Institute, Giessen, Germany (suburban landscape, in immediate vicinity to an arboreous environmental protection area), and at two field sites at the Bee Institute, Kirchhain, Germany (suburban landscape) in early September 2014 and 2015. At this time of the year, agricultural applications of neonicotinoids are uncommon in this region. All source colonies were briefly inspected, were healthy and showed no symptoms of bacterial, fungal, or viral disease.

### Feeding solution

We spiked 999 µl of a 50% sugar/water feeding solution with 1 µl acetone (GC Ultra Grade, Carl Roth, Germany). The sugar control group (sample SC) received feeding solutions spiked with pure acetone, whereas the other groups were presented with feeding solutions spiked with acetone containing imidacloprid (Sigma-Aldrich, St Louis, MO, USA), resulting in a 8.2 ng imidacloprid per µl feeding solution. For the individual feeding experiments, bees were isolated in 15 ml centrifuge tubes (SLG, Gauting, Germany) and starved for 1.5 h. The caps of the tubes were spotted with 5 µl of the feeding solution, representing a total imidacloprid dosage of 41 ng per bee. The starved bees ingested all of the solution immediately after the presentation. For the group feeding experiment, 10 worker bees were transferred to standard metal cages (8.5 × 6.5 × 4 cm) and starved for 1.5 h. Subsequently, caps with 50 µl of the feeding solution were placed into the cages, representing a total imidacloprid dosage of 410 ng per cage and 41 ng per bee, respectively. Since the treatments groups sugar control, RT1, and RT1mail were frozen one hour after the feeding of the single dose of imidacloprid, there was no need to provide them with additional food. The bees of the other treatment groups were paralysed and appeared to be “dead” until they became mobile again (approx. 46–48 hours after the feeding) but they received no additional food.

### Experimental design

We assigned 50 worker bees to each treatment. Each experiment (see Table 1) was carried out three times, and each repetition involved bees from a different hive. Each treatment was therefore applied to 150 bees in total.

**Table 1:** Treatment conditions of experimental groups.

Name	Treatment	Feeding condition	Time before freezing	Storage temperature before transport	Transport temperature	UV-exposure
SC	**Sugar control**	individual	**1 h**	freezer (ca. −80 °C)	dry ice (ca. −80 °C)	**−**
RT1	41 ng imidacloprid/bee	individual	**1 h**	freezer (ca. −80 °C)	dry ice (ca. −80 °C)	**−**
RT24	41 ng imidacloprid/bee	individual	**24 h**	freezer (ca. −80 °C)	dry ice (ca. −80 °C)	**−**
RT48	41 ng imidacloprid/bee	individual	**48 h**	freezer (ca. −80 °C)	dry ice (ca. −80 °C)	**−**
RT1mail	41 ng imidacloprid/bee	individual	**1 h**	**fridge (ca. 4 °C)**	**ambient temperature**	**−**
RT24UV	41 ng imidacloprid/bee	individual	**24 h**	freezer (ca. −80 °C)	dry ice (ca. −80 °C)	**+**
RT24GF	**410 ng imidacloprid/ten bees**	**group**	**24 h**	freezer (ca. −80 °C)	dry ice (ca. −80 °C)	**−**

### Impact of time at room temperature

In the initial degradation experiment, three groups of individually fed bees were frozen after different periods of exposure to room temperature (ca. 21°C, Table 1): one hour (sample RT1), 24 hours (sample RT24), and 48 hours (sample RT48). Until freezing, tubes and cages containing the bees were placed on laboratory benches; direct sunlight was avoided.

### Impact of transport method

Bees were individually exposed to imidacloprid, frozen for one hour (−20 °C), but subsequently stored in a fridge (−4 °C). Later, they were sent for analysis using a standard German parcel delivery service (DHL Paket GmbH, Bonn, Germany) at ambient temperatures (sample RT1mail). For these samples, we chose a selection of dispatch dates, including the days before weekends and bank holidays, so the delivery took between 1 and 6 days.

All other samples were sent by express delivery (TNT Express GmbH, Troisdorf, Germany) on dry ice and arrived on the same day at the analytical laboratory.

### Impact of UV radiation

A group of individually fed bees were exposed to 20 J/cm^2^ UV light one hour after feeding and were frozen after 24 hours at room temperature (sample RT24UV).

### Impact of feeding condition

In all our previous experiments bees were fed individually to minimise “dilution”-effects by trophallaxis (transfer of food droplets from one bee to another). In this experiment (sample RT24GF), the bees were kept in cages of 10 individuals and had access to the ten-fold volume (50 µl) of the imidacloprid spiked sugar solution. Every other factor was kept similar. After feeding, these bees were left at room temperature for 24 hours (comparable to RT24).

### Dose-dependent effects on locomotory behavior

To assess the effect of different dosages of imidacloprid, including the LD50_oral 48h_ (3.7 ng/bee) on the bee’s mobility, individual bees were placed in petri dishes (20 bees per treatment group, one replicate). After a starving period of 1.5 hours, the bees were exposed to 5 µl of feeding solution representing a total imidacloprid dosage of 0 ng, 3.7 ng, or 41 ng per bee. After one hour, bees were fed a 50% sucrose/water solution (v/v) *ad libitum* with a perforated Eppendorf tube through a hole in the lid of the petri dish. At different time points (one hour before exposure, one, 12, 24, 36, 48, 60, and 72 hours after exposure), the activity was examined. Immobility was defined as absence of any visible movements of body parts (including ventilation of the abdomen) or coordinated activity (e.g. standing, hanging, waking, etc.). Bees were observed for 30 seconds at each time interval.

### Chemical residue analysis

#### Preparation of samples for residue analysis of worker bees

A sample of worker bees (10 individuals, about 1 g) was weighed in a centrifuge tube made of glass (100 ml), and 50 μl of a surrogate standard solution (acetamiprid-d3, used to evaluate the analysis, not used for calculations) and 30 ml of a acetone/water-mixture (2/1, v/v) were added to every sample. The tubes were closed and left to stand for 30 minutes. The samples were homogenized for 3 minutes with a disperser and then centrifuged (10 minutes at 3,000 rpm). After centrifugation, 15 ml of the extract were removed and after addition of 5 ml sodium chloride-solution (20%) to this aliquot transferred onto a disposable cartridge filled with diatomaceous earth (ChemElut® cartridges, 20 ml, unbuffered, Agilent). After a waiting time of 15 minutes the samples were eluted with dichloromethane (2 × 50 ml). The eluates were evaporated to approximately 2 ml by using a rotary evaporator, then transferred to a graduated tube and evaporated to dryness with nitrogen, using a metal block thermostat with nitrogen blow device. The residual extract was taken up with 1 ml of a methanol/water mixture (1/1, v/v) containing the internal standards (imidacloprid-d4, clothianidin-d3), dissolved using an ultrasonic liquid mixer (30 seconds) and then put into the freezer (−18 °C) over night. On the next day, the samples were filtered cold (syringe filter: 0.2 µm).

### Identification and quantification of imidacloprid and its main metabolites

LC-MS/MS was used for the identification and quantification of the substances in the samples. The system used was a Prominence UFLC XR HPLC (SHIMADZU) coupled to a triple quadrupole mass spectrometer 4000 Q TRAP® (AB SCIEX) equipped with an electrospray ionization (ESI) source. Imidacloprid was measured in positive ion mode and its main metabolites imidacloprid-5-hxdroxy and imidacloprid-olefin were measured in negative ion mode. All three substances were identified by their retention time and three MRM-transitions. The residues in the samples were quantified with reference standards in matrix (concentrations: 0.05, 0.1, 0.5, 1, 5, 10, 25, 50 and 100 pg/µl). The quantification was carried out by the internal standard method. Certified pesticide standards were purchased from Dr. Ehrenstorfer (Augsburg, Germany, now LGC Standards, Wesel, Germany), except imidacloprid-5-hydroxy ([(6-Chloro-3-pyridinyl)methyl]-4,5-dihydro-2-(nitroamino)-1H-imidazol-5-ol) that was obtained from Bayer Crop Science (Frankfurt am Main, Germany). All solvents used were classified as highly pure for residue analysis and LC/MS. The residue values shown for the samples are averages of measurements out of duplicate injections of the sample extracts. The limit of detection (LOD) was determined as the lowest concentration tested in which the peak signal of the main MRM, which was used for quantification, was three times higher than the background noise of the chromatogram. The limit of quantification (LOQ) was 1 pg/µl for imidacloprid, 0.5 pg/µl for imidacloprid-5-hydroxy, and 5 pg/µl for imidacloprid-olefin, this refers to 2 µg/kg, 1 µg/kg and 10 µg/kg taking into account an initial sample weight of 1 g and the terms of the described residue analysis.

### Statistical analysis

To investigate

1. Whether rapid transportation on dry ice can preserve the level of residue left for analysis, linear regression models of residue-concentrations on time (in hours exposed to room temperature before freezing) and hives were used (including a potential interaction between time and hive).
2. If the degradation process can be slowed down by short freezing, a two-factorial ANOVA for the imidacloprid-concentration on treatment and hive (including their interaction) was employed, followed by multiple comparisons with a reference group (here RT1mail) and calculation of simultaneous confidence intervals for the respective differences.
3. Whether exposure to UV light affects residue degradation in honeybee samples, the same analyses were applied as in (2).
4. If imidacloprid residues in individually fed bees are different from those in bees that were fed in groups, the analysis was two-fold: Bartlett’s several samples test for homogeneity of variances of imidacloprid concentrations in combinations of the RT24 and RT24GF treatments and hives was used to see if measurement quality is affected by feeding. To determine if quantified residue levels are affected by the feeding method, the same analyses were applied as in (2) but with RT24GF as reference.

In all analyses a decadic (base 10) log-transformation of the respective response variable was considered and used if model diagnostics (like q-q-plots and scale-location-plots for the model residuals) suggested it to achieve homoscedastic normality for the data.

All statistical calculations and analyses were performed using R 3.3.1 (R Core Team, 2016) including the packages car (Fox and Weisberg, 2011; for q-q-plots with confidence intervals) and multcomp (Hothorn *et al*., 2008; for multiple pairwise comparisons).

## Electronic supplementary material

Supplementary information
