# Supplementary material for: Temporal dynamics of whole body residues of the neonicotinoid insecticide imidacloprid in live or dead honeybees
Source: Sci Rep. 2017 Jul 24;7:6288. doi: 10.1038/s41598-017-06259-z (PMC5524747; doi:10.1038/s41598-017-06259-z)
Supplement: Supplementary file 1 — Supplementary information [file 41598_2017_6259_MOESM1_ESM.pdf]

# Supplement of the article

## Temporal dynamics of whole body residues of the neonicotinoid insecticide imidacloprid in live or dead honeybees

Matthias Schott[1,2], Gabriela Bischoff[3], Gerrit Eichner[4], Andreas Vilcinskas[2,5], Ralph Büchler[6], Marina Doris Meixner[6], Annely Brandt[5,6*]

[1]Institute for Zoology, University of Cologne, Germany;

[2]Institute for Insectbiotechnology, Justus-Liebig University of Giessen, Giessen, Germany;

[3]Julius Kühn-Institut - Federal Research Centre for Cultivated Plants, Institute for Bee Protection, Germany;

[4]Mathematical Institute, Justus-Liebig University of Giessen, Giessen, Germany;

[5]Fraunhofer Institute for Molecular Biology and Applied Ecology, Department of Bioresources, Giessen, Germany;

[6]LLH Bee Institute, Erlenstr. 9, 35274 Kirchhain, Germany

Statistical Report page 1

Supplement Figure 1 page 58

# Technical report for the article "Temporal dynamics of whole body residues of the neonicotinoid insecticide imidacloprid in live or dead honeybees"

by


Dr. Gerrit Eichner
Mathematical Institute
Justus-Liebig-University Giessen
Arndtstr. 2, D-35392 Giessen
Tel.: +49-(0)641-9932104
Email: gerrit.eichner@math.uni-giessen.de
URL: http://www.uni-giessen.de/eichner

and

Dr. Matthias Schott
Institute for Zoology
Cologne Biocenter
University of Cologne
Zülpicher Str. 47b, D-50674 Köln
Tel.: +49-(0)221-470-6637
Email: m.schott@uni-koeln.de
URL: http://www.uni-koeln.de/math-nat-fak/zoologie/oekologie/agvonelert/

Authors of article: Matthias Schott, Gabriela Bischoff, Gerrit Eichner, Andreas Vilcinskas, Ralph Büchler, Marina Doris Meixner, Annely Brandt


May 1, 2017

## Contents


**0 R-technical preparations** **1**

**1 Raw data** **2**
1.1 Import and conversion . . . . . . . . 2
1.2 Inspection of the imported raw data . . . . . . . . 5

**2 The actual questions** **7**
2.1 Question 1: Time before freezing . . . . . . . . 8
2.1.1 Imidachloprid . . . . . . . . 8
2.1.2 5-hydroxyimidacloprid . . . . . . . . 17
2.1.3 Oelefin . . . . . . . . 23
2.2 Question 2: Short freezing before mailing . . . . . . . . 29
2.2.1 Imidachloprid . . . . . . . . 29
2.3 Question 3: UV-light . . . . . . . . 37
2.3.1 Imidachloprid . . . . . . . . 37
2.4 Question 4: Single vs. group feeding . . . . . . . . 45
2.4.1 Imidachloprid . . . . . . . . 45
2.4.1.1 Comparing variances . . . . . . . . 47
2.4.1.2 Comparing locations . . . . . . . . 51

**3 Software & References** **57**

**4 SFig1** **58**


## List of Figures


1 Q1: Imidachloprid by hive . . . . . . . . 9

2 Q1: Imidachloprid vs. time . . . . . 9
3 Q1: $\log_{10}$(Imidachloprid) on time and hive, model (2): Augmented prediction plots . . . . . 12
4 Q1: Imidachloprid, models (1) and (2): Diagnostic plots . . . . . 13
5 Q1: $\log_{10}$(Imidachloprid) on time, model (4): Augmented prediction plots . . . . . 16
6 Q1: 5-hydroxyimidacloprid by hive . . . . . 17
7 Q1: 5-hydroxyimidacloprid vs. time . . . . . 17
8 Q1: 5-hydroxyimidacloprid, models (1) and (2): Diagnostic plots . . . . . 20
9 Q1: 5-hydroxyimidacloprid on time and hive, model (1): Augmented prediction plots . . . . . 21
10 Q1: Oelefin by hive . . . . . 23
11 Q1: Oelefin vs. time . . . . . 23
12 Q1: Oelefin, models (1) and (2): Diagnostic plots . . . . . 26
13 Q1: Oelefin on time and hive, model (1): Augmented prediction plots . . . . . 27
14 Q2: Imidachloprid by method and hive . . . . . 30
15 Q2: Imidachloprid by method per hive with interaction profiles . . . . . 30
16 Q2: Imidachloprid, models (8) and (9): Diagnostic plots . . . . . 32
17 Q2: Imidachloprid by method per hive: Simultaneous confidence intervals for MCC . . . . . 34
18 Q2: Imidachloprid by method and hive: Simultaneous confidence intervals for MCC . . . . . 36
19 Q3: Imidachloprid by method and hive . . . . . 38
20 Q3: Imidachloprid by method per hive with interaction profiles . . . . . 38
21 Q3: Imidachloprid, models (8) and (9): Diagnostic plots . . . . . 40
22 Q3: Imidachloprid by method per hive: Simultaneous confidence intervals for MCC . . . . . 42
23 Q3: Imidachloprid by method and hive: Simultaneous confidence intervals for MCC . . . . . 44
24 Q4: Imidachloprid by method and hive . . . . . 46
25 Q4: Imidachloprid by method per hive with interaction profiles . . . . . 46
26 Q4: Imidachloprid, models (8) and (9): Diagnostic plots . . . . . 52
27 Q4: Imidachloprid by method per hive: Simultaneous confidence intervals for MCC . . . . . 54
28 Q4: Imidachloprid by method and hive: Simultaneous confidence intervals for MCC . . . . . 56
29 **Supplementary Fig.1**: Dose-dependent effects on locomotory behaviour. Individual honeybees were fed with a single dose of sugar syrup (control) or a dosage of 3.7 and 41 ng/bee imidacloprid (20 bees per treatment group, single experiment). After one, 12, 24, 36, 48, 60, and 72 hours bees were examined for activity. The locomotory behaviour was categorised in (black) immobility, (shaded area) movements of body parts, including ventilation movements of the abdomen, and (white) coordinated activity (e.g. standing, hanging, or walking). After 12 hours, 68% of the bees of the 41 ng treatment group were immobile and 31% only showed movement of body parts. After 48 hours the 41 ng treatment group had the same number of completely immobile bees as the control but the percentage of bees with coordinated movements was lower in this group. . . . . . 58

# List of Tables

1 Q1: Imidachloprid: Regression on time and hive, model (1) . . . . . 10
2 Q1: $\log_{10}$(Imidachloprid): Regression on time and hive, model (2) . . . . . 11
3 Q1: $\log_{10}$(Imidachloprid): ANOVA table for regression model (2) . . . . . 14
4 Q1: $\log_{10}$(Imidachloprid): Regression on time, model (4) . . . . . 15
5 Q1: $\log_{10}$(Imidachloprid): ANOVA table for the influence of hive in model (2) . . . . . 15
6 Q1: $\log_{10}$(Imidachloprid): estimates with confidence intervals in model (4) . . . . . 16
7 Q1: 5-hydroxyimidacloprid: Regression on time and hive, model (1) . . . . . 18
8 Q1: $\log_{10}$(5-hydroxyimidacloprid): Regression on time and hive, model (2) . . . . . 19
9 Q1: 5-hydroxyimidacloprid: ANOVA table for regression model (1) . . . . . 22
10 Q1: 5-hydroxyimidacloprid: estimates with confidence intervals in model (4) . . . . . 22
11 Q1: Oelefin: Regression on time and hive, model (1) . . . . . 24
12 Q1: $\log_{10}$(Oelefin): Regression on time and hive, model (2) . . . . . 25
13 Q1: $\log_{10}$(Oelefin): ANOVA table for regression model (2) . . . . . 28
14 Q1: $\log_{10}$(Oelefin): estimates with confidence intervals in model (4) . . . . . 28
15 Q2: Imidachloprid: Two-factorial ANOVA on hive and method with interaction, model (8) . 31
16 Q2: $\log_{10}$(Imidachloprid): Two-factorial ANOVA on hive and method w. interaction, model (9) 31
17 Q2: Imidachloprid by method per hive: multiple comparisons with a control . . . . . 33
18 Q2: Imidachloprid by method per hive: Simultaneous confidence intervals for MCC . . . . . 34

19 Q2: Imidachloprid by method and hive: confidence intervals of model (9) . . . . . . . . . . . 35
20 Q2: Imidachloprid by method and hive: mult. comp. w. a control in model (9) w/o interaction 35
21 Q2: Imidachloprid by method and hive: Simultaneous confidence intervals for MCC . . . . . 36
22 Q2: Imidachloprid: Two-factorial ANOVA on hive and method with interaction, model (8) . 39
23 Q3: $\log_{10}$(Imidachloprid): Two-factorial ANOVA on hive and method w. interaction, model (9) 39
24 Q3: Imidachloprid by method per hive: multiple comparisons with a control . . . . . . . . . . 41
25 Q3: Imidachloprid by method per hive: Simultaneous confidence intervals for MCC . . . . . . 42
26 Q3: Imidachloprid by method and hive: confidence intervals of model (8) . . . . . . . . . . . 43
27 Q3: Imidachloprid by method and hive: mult. comp. w. a control in model (8) w/o interaction 43
28 Q3: Imidachloprid by method and hive: Simultaneous confidence intervals for MCC . . . . . 44
29 Q4: Imidachloprid: Various variances . . . . . . . . . . . . . . . . . . . . . . . . . . . . . . . 47
30 Q4: $\log_{10}$(Imidachloprid): Various variances . . . . . . . . . . . . . . . . . . . . . . . . . 47
31 Q4: Imidachloprid: Bartlett's test for homoscedasticity . . . . . . . . . . . . . . . . . . . . . . 48
32 Q4: $\log_{10}$(Imidachloprid): Bartlett's test for homoscedasticity . . . . . . . . . . . . . . . . . . . 48
33 Q4: Imidachloprid: Pairwise $F$-tests for homoscedasticity . . . . . . . . . . . . . . . . . . . . 49
34 Q4: $\log_{10}$(Imidachloprid): Pairwise $F$-tests for homoscedasticity . . . . . . . . . . . . . . . . . 49
35 Q4: Imidachloprid: Pairwise $F$-tests for homoscedasticity per hive . . . . . . . . . . . . . . . 50
36 Q4: Imidachloprid: Two-factorial ANOVA on hive and method with interaction, model (8) . 51
37 Q4: $\log_{10}$(Imidachloprid): Two-factorial ANOVA on hive and method w. interaction, model (9) 51
38 Q4: Imidachloprid by method per hive: multiple comparisons with a control . . . . . . . . . . 53
39 Q4: Imidachloprid by method per hive: Simultaneous confidence intervals for MCC . . . . . . 54
40 Q4: Imidachloprid by method and hive: confidence intervals of model (9) . . . . . . . . . . . 55
41 Q4: Imidachloprid by method and hive: mult. comp. w. a control in model (8) w/o interaction 55
42 Q4: Imidachloprid by method and hive: Simultaneous confidence intervals for MCC . . . . . 56

# 0 R-technical preparations

The ("contributed") R-packages that will be used in the following are loaded:

```
> library( Hmisc)
> library( car)
> library( lattice)
> library( multcomp)
```

Storing Rs current options for their recovery after generating this report:

```
> startoptions <- options()
```

# 1 Raw data

The original-raw data had been saved in several MS-Excel-sheets and have been exported as ‘comma-separated values’ (CSV) files, which fields have been separated by semicolon (;) and in case of the sugar control “SC” groups missing values (below limit of detection) have been noted as NA (= ‘not available’, synonymous for missing values). The decimal sign is the dot (.). The columns in the CSV-file possess names (in its first row). The file names are as follows:

```
> (Filenames <- list.files( "Daten",
+                           pattern = glob2rx( "*.csv", trim.head = TRUE)))

[1] "Hydroxy.csv" "Imi.csv"     "Oel.csv"

> Filepaths <- file.path( "Daten", Filenames)
> names( Filepaths) <- names( Filenames) <-         # File names w/o last 4 cha-
+   substring( Filenames, 1, nchar( Filenames) - 4) # racters (file name extension)
> NFiles <- length( Filenames)     # Number of csv-files
```

## 1.1 Import and conversion

After passing an initial, minimal format check these 3 CSV-files are imported into a ’data frame’ in R, Version 3.3.2 [1, R Development Core Team]. These data frames are combined into a list with named components.

```
> sapply( Filepaths, function( fn) unique( count.fields( fn, sep = ";")))
> Files <- lapply( Filepaths, read.csv, sep = ";")   # List of imported data frames
```

To assess the correct import the structure and contents of each of these 3 data frames are presented:

```
> invisible(
+  lapply( names( Files),
+          function( dn) {
+            X <- Files[[ dn]]
+            cat0( "\nName of data frame: ", dn,
+                  "\n########################\nStructure:\n");   str( X)
+            cat( "\nHead:\n");      print( head( X))
+            cat( "\nSummary:\n");   print( summary( X));   cat( "\n")
+            } ) )

Name of data frame: Hydroxy
########################
Structure:
'data.frame':        105 obs. of  3 variables:
 $ hive  : int  1 1 1 1 1 1 1 1 1 1 ...
 $ method: Factor w/ 7 levels "SC","t1","t1mail",..: 2 2 2 2 2 4 4 4 4 4 ...
 $ conc  : num  1.8 1 2.2 1.9 2.5 1.7 1.3 1.6 1.5 1.6 ...

Head:
  hive method conc
1    1     t1  1.8
2    1     t1  1.0
3    1     t1  2.2
4    1     t1  1.9
5    1     t1  2.5
6    1    t24  1.7

Summary:
      hive        method       conc
 Min.   :1   SC      :15   Min.   :0.100
 1st Qu.:1   t1      :15   1st Qu.:1.300
 Median :2   t1mail  :15   Median :1.500
 Mean   :2   t24     :15   Mean   :1.551
```

```
 3rd Qu.:3   t24group:15   3rd Qu.:1.900
 Max.   :3   t24UV   :15   Max.   :3.000
             t48     :15   NA's   :11


Name of data frame: Imi
########################
Structure:
'data.frame':        105 obs. of  3 variables:
 $ hive  : int  1 1 1 1 1 1 1 1 1 1 ...
 $ method: Factor w/ 7 levels "SC","t1","t1mail",..: 2 2 2 2 2 4 4 4 4 4 ...
 $ conc  : num  15.94 4.08 14.85 10.7 10.35 ...

Head:
  hive method  conc
1    1     t1 15.94
2    1     t1  4.08
3    1     t1 14.85
4    1     t1 10.70
5    1     t1 10.35
6    1    t24  8.58

Summary:
      hive        method        conc
 Min.   :1   SC      :15   Min.   : 0.250
 1st Qu.:1   t1      :15   1st Qu.: 4.287
 Median :2   t1mail  :15   Median : 7.893
 Mean   :2   t24     :15   Mean   : 8.915
 3rd Qu.:3   t24group:15   3rd Qu.:12.828
 Max.   :3   t24UV   :15   Max.   :25.241
             t48     :15   NA's   :12


Name of data frame: Oel
########################
Structure:
'data.frame':        105 obs. of  3 variables:
 $ hive  : int  1 1 1 1 1 1 1 1 1 1 ...
 $ method: Factor w/ 7 levels "SC","t1","t1mail",..: 2 2 2 2 2 4 4 4 4 4 ...
 $ conc  : num  1.6 1.1 2.4 2.3 2.4 1.3 0.9 1.1 1 1.3 ...

Head:
  hive method conc
1    1     t1  1.6
2    1     t1  1.1
3    1     t1  2.4
4    1     t1  2.3
5    1     t1  2.4
6    1    t24  1.3

Summary:
      hive        method        conc
 Min.   :1   SC      :15   Min.   :0.300
 1st Qu.:1   t1      :15   1st Qu.:1.100
 Median :2   t1mail  :15   Median :1.300
 Mean   :2   t24     :15   Mean   :1.355
 3rd Qu.:3   t24group:15   3rd Qu.:1.600
 Max.   :3   t24UV   :15   Max.   :2.500
             t48     :15   NA's   :16
```

To ensure that all data frames inherit the same order of rows, the rows are ordered according to the first two columns:

```
> Files <- lapply( Files, function( x) x[ order( x[[ 1]], x[[ 2]]),] )
```

Subsequently, the last column of each data frame is renamed into the respective original file name:

```
> Files <- lapply( names( Files), function( dn) {
+                                    X <- Files[[ dn]]
+                                    names( X)[ ncol( X)] <- dn
+                                    X } )
```

Finally, the data frames are merged into one by extracting from each data frame in `Files` all columns *without* `hive` and `method` and merging these into a new data frame called `Bees`. Then, the columns `hive` and `method` of the *first* data frame in `Files` are padded on the left to `Bees` as its ("new") first two columns:

```
> not <- c( "hive", "method")
> Bees <- as.data.frame( lapply( Files, function( x) x[ -match( not, names( x))]))
> Bees <- cbind( Files[[ 1]][ not], Bees)
```

The data frame `Bees` consists now of 105 rows and 5 columns (= variables) named `hive`, `method`, `Hydroxy`, `Imi` and `Oel`. Below the following piece of code one can see how they are going to be renamed (*old* `->` *new*):

```
> new.variable.names <- c( "hive", "method", "Hyd", "Imi", "Oel")
> names( new.variable.names) <- names( Bees)
> names( Bees) <- new.variable.names
> cat.from.to( names( new.variable.names), format( new.variable.names))
```

```
hive    -> hive       method  -> method     Hydroxy -> Hyd
Imi     -> Imi        Oel     -> Oel
```

Now, two variables in `Bees` are restructured and recoded, respectively. Additionally, two columns are changed in their order (only due to 'aesthetic' reasons):

- The still numeric variable `hive` is transformed into a factor variable with three levels presented below:

  ```
  > Bees$hive <- factor( Bees$hive, levels = 1:3, labels = paste0( "h", 1:3))
  > levels( Bees$hive)
  ```

  ```
  [1] "h1" "h2" "h3"
  ```

- The levels of the factor variable `method` are slightly recoded:

  ```
  > Bees$method <- factor( Bees$method,
  +   levels = c( "SC", "t1",  "t24",  "t48",  "t1mail",  "t24UV",  "t24group"),
  +   labels = c( "SC", "RT1", "RT24", "RT48", "RT1mail", "RT24UV", "RT24GF"))
  ```

- The columns 3 and 4 (i.e., `Hyd` and `Imi`) are interchanged:

  ```
  > Bees <- Bees[ c( 1:2, 4, 3, 5)]
  ```

There are some `NA`s ("missing values") in the columns 3, 4 and 5 (i.e., `Imi`, `Hyd` and `Oel`) of `Bees`:

```
> xtabs( is.na( Bees[ ix]) ~ hive + method, data = Bees)
```

```
, ,  = Imi

    method
hive SC RT1 RT24 RT48 RT1mail RT24UV RT24GF
  h1  5   0    0    0       0      0      0
  h2  5   0    0    0       0      0      0
  h3  2   0    0    0       0      0      0
```

```
, ,  = Hyd

    method
hive SC RT1 RT24 RT48 RT1mail RT24UV RT24GF
  h1  5   0    0    0       0      0      0
  h2  5   0    0    0       0      0      0
  h3  1   0    0    0       0      0      0

, ,  = Oel

    method
hive SC RT1 RT24 RT48 RT1mail RT24UV RT24GF
  h1  5   0    0    0       0      0      0
  h2  5   0    0    0       0      0      1
  h3  5   0    0    0       0      0      0
```

However, neither the `method SC` nor `Oel` in `method RTGF24` will be relevant for the analyses to come, so that we do not have to take any care of them.

## 1.2 Inspection of the imported raw data

For each generated data frame: example excerpts and summarizing inspection of contents:

```
> op <- options( width = 105)
> for( dfname in c( "Bees")) {
+   x <- get( dfname)
+   k <- 11    # + 5*(dfname == "?")
+   cat0( "\n \\bigskip \nThe first as well as the last ", k, " of ", nrow( x),
+         " rows of the \\texttt{", dfname, "} data frame:\n")
+   cat( "\\begin{verbatim}\n")
+   print( head( x, k))
+   cat( "....\n")
+   print( tail( x, k))
+   cat( "\\end{verbatim}\n\n \\bigskip")
+   cat0( "\nAnd the ``summary statistics'' of all variables of ",
+         "\\texttt{", dfname, "}:\n")
+   cat( "\\begin{verbatim}\n")
+   print( summary( x))
+   cat( "\\end{verbatim}\n\n \\hrule \n \\bigskip \n")
+   };     options( op)
```

The first as well as the last 11 of 105 rows of the `Bees` data frame:

```
   hive  method   Imi Hyd Oel
26   h1      SC    NA  NA  NA
27   h1      SC    NA  NA  NA
28   h1      SC    NA  NA  NA
29   h1      SC    NA  NA  NA
30   h1      SC    NA  NA  NA
1    h1     RT1 15.94 1.8 1.6
2    h1     RT1  4.08 1.0 1.1
3    h1     RT1 14.85 2.2 2.4
4    h1     RT1 10.70 1.9 2.3
5    h1     RT1 10.35 2.5 2.4
16   h1 RT1mail  6.66 2.0 2.5
....
    hive method    Imi Hyd Oel
```

```
105   h3 RT24GF 20.018 2.1 1.6
96    h3 RT24UV  3.435 1.2 1.3
97    h3 RT24UV  2.699 1.4 1.6
98    h3 RT24UV  4.287 1.9 1.2
99    h3 RT24UV  2.455 1.7 1.4
100   h3 RT24UV  4.164 1.7 1.4
81    h3   RT48  3.706 2.9 1.3
82    h3   RT48  1.731 2.5 1.2
83    h3   RT48  4.396 3.0 1.9
84    h3   RT48  3.077 2.9 1.4
85    h3   RT48  3.746 2.4 1.3
```

And the "summary statistics" of all variables of `Bees`:

```
 hive        method        Imi              Hyd             Oel
 h1:35   SC      :15   Min.   : 0.250   Min.   :0.100   Min.   :0.300
 h2:35   RT1     :15   1st Qu.: 4.287   1st Qu.:1.300   1st Qu.:1.100
 h3:35   RT24    :15   Median : 7.893   Median :1.500   Median :1.300
         RT48    :15   Mean   : 8.915   Mean   :1.551   Mean   :1.355
         RT1mail:15    3rd Qu.:12.828   3rd Qu.:1.900   3rd Qu.:1.600
         RT24UV :15    Max.   :25.241   Max.   :3.000   Max.   :2.500
         RT24GF :15    NA's   :12       NA's   :11      NA's   :16
```

# 2 The actual questions

The actual questions, which are going to be examined in the following, are:

1. Does Imidacloprid and its known metabolites degrade in dead bees at room temperature? Comparison of RT1, RT24, and RT48 with respect to
   (a) Imidacloprid (cf. to §2.1.1)
   (b) 5-hydroxyimidacloprid (cf. to §2.1.2)
   (c) Olefin (cf. to §2.1.3)
2. Can the process of degradation be slowed down by short freezing? (Comparison: RT1mail vs. each of RT1, RT24, and RT48)

   (Cf. to sec. 2.2)
3. Does heavy exposure to UV-light accelerate the rate of degradation? (Comparison: RT24UV with RT24, but also with RT1 and RT48)

   (Cf. to sec. 2.3)
4. Does the feeding method (single vs. group feeding) influence the measurement results and the quality (here: *variance*) of the data? (Comparison: RT24GF with RT24, but also with RT1 and RT48)

   (Cf. to sec. 2.4)

**Remark:** For all following statistical analyses we set the level of significance to $\alpha = 5\%$ and the confidence level to $1 - \alpha = 95\%$:

```
> alpha <- 0.05
```

## 2.1 Question 1: Time before freezing

Preparations:

```
> lev.of.int <- c( "RT1", "RT24", "RT48")   # treatment levels of interest
```

1. Extracting the rows of `Bees`, whose corresponding elements in `method` are in the set of "values" {`"RT1"`, `"RT24"`, `"RT48"`}, i.e., which belong to the treatments RT1, RT24 or RT48, and storage of the "extract" in a new data frame named `BeesX`. Thereafter, generating a variable `time` by extracting the 3rd until (at most) 4th symbol out of the abbreviations RT1, RT24 or RT48, respectively, and converting that into mode `numeric`, i.e., a number (if possible):

```
> BeesX <- droplevels( subset( Bees, subset = method %in% lev.of.int))
> BeesX$time <- as.numeric( substr( BeesX$method, 3, 4))
```

2. Generating variables which contain some of the "values" which will be needed and repeatedly (!) used in the following paragraphs, so that a flexible and passably efficient modifiable code can be designed, that is also simply reusable for subsequent paragraphs:

```
> Response <- c( Imidachloprid = "Imi");   ContCovar <- "time"
> Treatment <- "method";   Group <- "hive"
```

### 2.1.1 Imidachloprid

Question: Does Imidachloprid degrade in dead bees at room temperature?

Asked a bit more precisely: Does the average concentration of Imidachloprid (in dead bees at room temperature) decrease along time?

To answer this question we perform a (log-)linear regression of Imidachloprid on time $t$ (with data at the times $t \in \{1, 24, 48\}$).

Points to consider: Repeated measurements on the same hive *usually* induce dependencies (and hence correlation) within each group of measurements from the same hive. However, thorough model diagnostics (in particular, inspection of the distribution of the residuals) revealed no evidence for a correlation structure here. In addition, the small number of only three groups (hives) did not warrant the use of a mixed-effects regression model (which could typically be employed for the analysis of data with a hierarchical grouping structure like the present).

**Graphical Exploratory Data Analysis (EDA)**

Fig. 1 presents a first exploratory graph of the Imidachloprid data. It displays – without consideration of a potential influence of time – the distribution of the Imidachloprid values separately for each hive using "strip plots" (also called one-dimensional scatter plots) on different scales: the left panel uses the original measurement scale, the right one the decimal logarithmic scale, i.e., $\log_{10}$-scale (for reasons given below).

Fig. 2 shows – color-coded for all hives overlaid – the distribution of Imidachloprid values versus time on the original scale (left) and on the decimal logarithmic scale (right). The strip plots on the original scale appear to present a slight heteroscedasticity (i. e., inhomogeneity of variances) of Imidachloprid along time: the variability (spread) of the data seems to decline with falling average Imidachloprid values (i.e., with advancing time), across hives. This is (maybe a bit over-)compensated by the (typically variance-stabilizing) logarithmic transformation. (For details regarding the log-transformation see the following remarks.)

**Remarks:** Any logarithmic transformation – due to its non-linearity – "stretches" the lower end of the measurement scale relatively stronger than the upper end. Consequently, "cluttered" data points at the lower end of the scale become separated and wide-spread data points at the upper end get pushed together. This has typically two statistically useful consequences: it may symmetrize an asymmetric data distribution and it may homogenize variances. In addition, it ensures that the log-linearly modelled response values yield positive values on the original measurement scale (after re-transforming the model with the antilogarithm, i.e., exponentiation with the base 10).

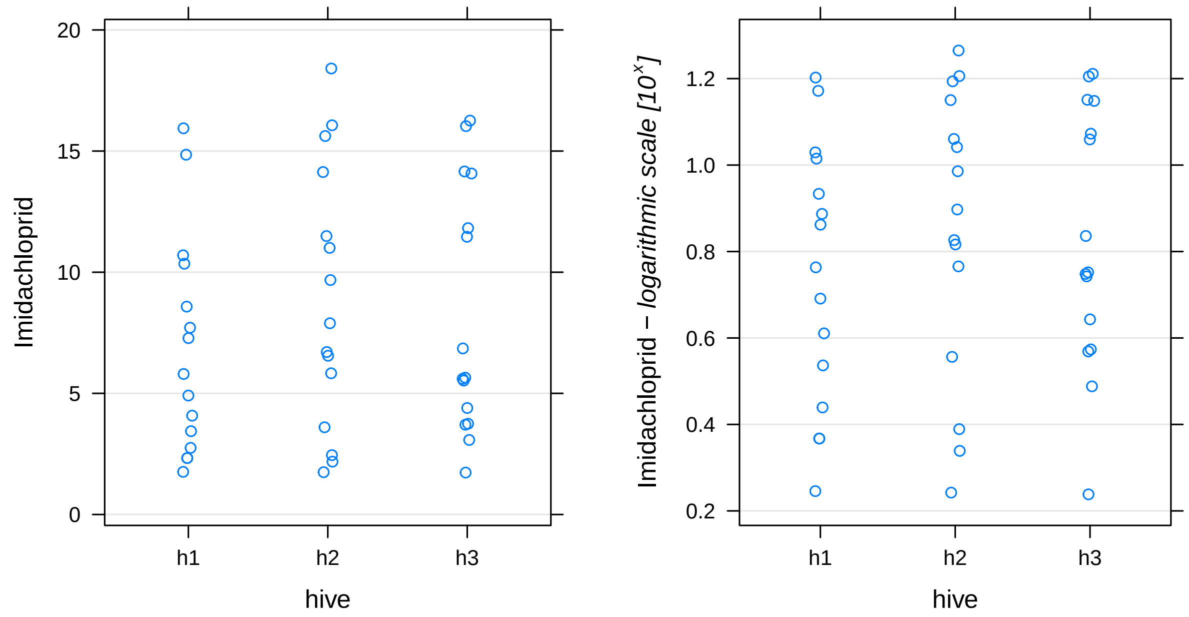


Figure 1: Imidachloprid by hive: on the original measurement scale in the left panel, on the decimal logarithmic scale in the right. For details see text. To avoid potential overlap of plotting symbols (circles) of tied observations, i.e., of values which are very close to each other or even identical, their horizontal *positions* (but, of course, not the original data on which all subsequent analyses are based) are slightly, randomly "jittered". (File names: left: *MS-Q1_EDA_Imi_by_hive_NOLOG.pdf*, right: *MS-Q1_EDA_Imi_by_hive_LOG.pdf*)

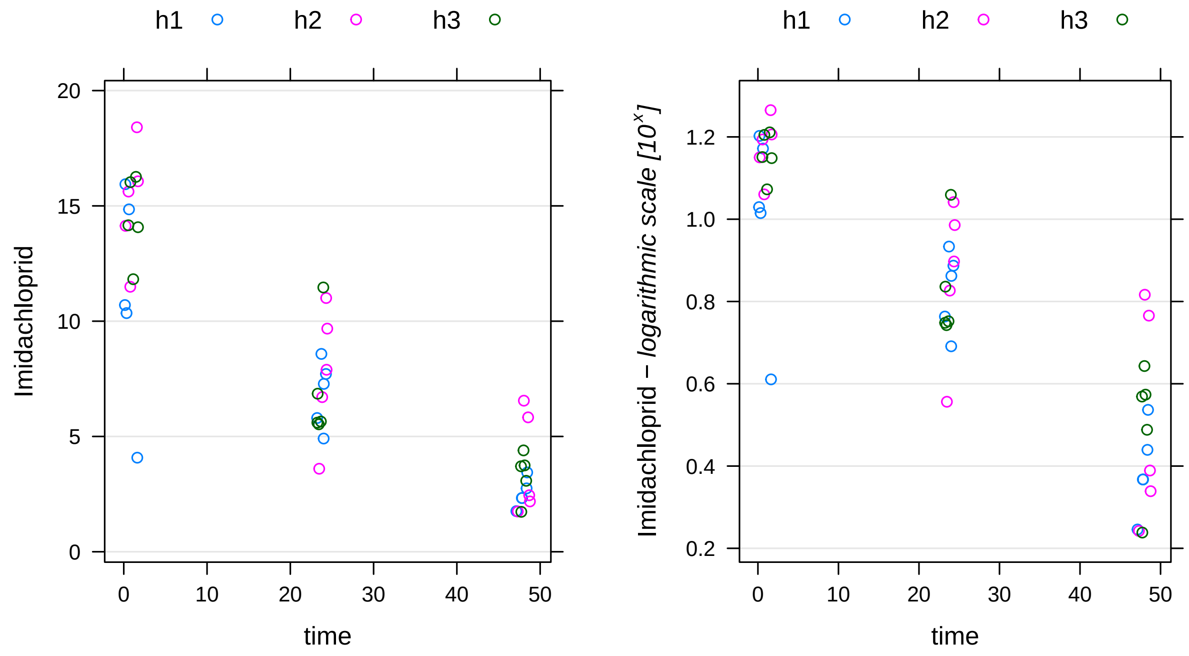


Figure 2: Imidachloprid (left on original scale, right on $\log_{10}$-scale) vs. time with hive-specific colors for the plotting symbols (horizontally jittered; for explanations see caption of fig. 1). For details see text. (File names: left: *MS-Q1_EDA_Imi_vs_time_NOLOG.pdf*, right: *MS-Q1_EDA_Imi_vs_time_LOG.pdf*)

**Model fitting**

Tab. 1 contains a **linear regression model of Imidachloprid on time** with (arbitrary) regression lines along time that **vary with hive** in their vertical position *and* in their slope ("interaction"). This allows to **assess if a significant time effect is present** (across hives) and if there is a difference between hives with respect to the hive-specific Imidachloprid-level or with respect to the hive-specific Imidachloprid-trend along time. (Such models are also known as "analyis of covariance" (or ANCOVA) models.)

Table 1: Regression of Imidachloprid on time and hive with interaction.

```
>    # 'building' the model formula using variables (for the sake of flexibility):
> form <- formula( paste( Response, "~", Group, "*", ContCovar))
> fit <- lm( form, data = BeesX)  # fitting the linear model.
> fit <- update( fit, ~ .)  # use of update() here is just a trick to see the model
>                           # formula in evaluated form in the following outputs.
> summary( fit)

Call:
lm(formula = Imi ~ hive + time + hive:time, data = BeesX)

Residuals:
    Min      1Q  Median      3Q     Max 
-7.0736 -1.1155  0.2569  1.5322  4.7864 

Coefficients:
            Estimate Std. Error t value Pr(>|t|)    
(Intercept) 11.33789    1.04249  10.876 2.25e-13 ***
hiveh2       3.43777    1.47430   2.332   0.0250 *  
hiveh3       2.68601    1.47430   1.822   0.0761 .  
time        -0.18427    0.03364  -5.478 2.75e-06 ***
hiveh2:time -0.05758    0.04757  -1.210   0.2335    
hiveh3:time -0.05209    0.04757  -1.095   0.2803    
---
Signif. codes:  0 '***' 0.001 '**' 0.01 '*' 0.05 '.' 0.1 ' ' 1

Residual standard error: 2.5 on 39 degrees of freedom
Multiple R-squared:  0.7775,	Adjusted R-squared:  0.749 
F-statistic: 27.26 on 5 and 39 DF,  p-value: 9.326e-12
```

Short formal representation of the model in tab. 1:

There were three points in time (remember: $t \in \{1, 24, 48\}$) at which the residue level measurements were taken. For hive $h \in \{1, 2, 3\}$ in bee-group $i \in \{1, \ldots, 5\}$ the observed residue level at time $t_j$ with $j \in \{1, 2, 3\}$ is denoted $Y_{hi}(t_j)$ and modelled as:

$$Y_{hi}(t_j) = \beta_0 + \alpha_h + \beta_1 \cdot t_j + \gamma_h \cdot t_j + e_{hij} \qquad (1)$$

with both $\alpha_1$ and $\gamma_1$ set to 0 (zero).

The R-output in short: the `Estimate`-column of the `Coefficients`-block in tab. 1 contains the estimated values for the regression coefficients $\beta_0$, the $\alpha_h$s, $\beta_1$, and the $\gamma_h$s in its rows (starting with `(Intercept)`). The last column `Pr(>|t|)` presents the $p$-values of the significance tests for the respective coefficients. The `Multiple R-squared` value is a goodness-of-fit measure and quantifies the proportion of the observed total variability of the response variable that can be "explained" by the regression model.

Tab. 2 contains almost the same model as tab. 1. The only difference is that the response variable is $\log_{10}$-transformed (so that this could be called a log-linear regression model).

Fig. 3 visualizes in its left part the hive-specific estimated regression lines of the fitted model on the $\log_{10}$-scale in separate so-called panels, one per hive, augmented by the raw data (as they are already presented in fig. 2 on the right). In its right part fig. 3 shows the respective estimated regression functions re-transformed (using the antilogarithm, i.e., exponentiation with the base 10) onto the original scale (and also augmented by the raw data as they are seen in fig. 2 on the left).

Table 2: Regression of the transformed response variable $\log_{10}$(Imidachloprid) on time and hive with interaction.

```
>    # Here, update() modifies only the response-side
>    # of the model formula and refits the model:
> summary( fitlog <- update( fit, log10( .) ~ .))

Call:
lm(formula = log10(Imi) ~ hive + time + hive:time, data = BeesX)

Residuals:
     Min       1Q   Median       3Q      Max
-0.43682 -0.08180  0.00217  0.10538  0.30212

Coefficients:
              Estimate Std. Error t value Pr(>|t|)
(Intercept)  1.0605948  0.0662384  16.012  < 2e-16 ***
hiveh2       0.1325657  0.0936752   1.415    0.165
hiveh3       0.1077569  0.0936752   1.150    0.257
time        -0.0131128  0.0021375  -6.135 3.37e-07 ***
hiveh2:time -0.0010311  0.0030228  -0.341    0.735
hiveh3:time -0.0008253  0.0030228  -0.273    0.786
---
Signif. codes:  0 '***' 0.001 '**' 0.01 '*' 0.05 '.' 0.1 ' ' 1

Residual standard error: 0.1589 on 39 degrees of freedom
Multiple R-squared:  0.7662,       Adjusted R-squared:  0.7363
F-statistic: 25.57 on 5 and 39 DF,  p-value: 2.395e-11
```

Short formal representation of the model in tab. 2:
For hive $h \in \{1, 2, 3\}$ in bee-group $i \in \{1, \dots, 5\}$ the observed residue level at time $t_j$ with $j \in \{1, 2, 3\}$ is denoted $Y_{hi}(t_j)$ and modelled as:

$$\log_{10}(Y_{hi}(t_j)) = \beta_0 + \alpha_h + \beta_1 \cdot t_j + \gamma_h \cdot t_j + e_{hij} \tag{2}$$

with both $\alpha_1$ and $\gamma_1$ set to 0 (zero). This model is described in short together with the corresponding R-output after equation (1).

The right part of fig. 3 shows the re-transformed estimated hive-specific regression functions

$$\hat{y}_h(t) = 10^{\left(\hat{\beta}_0 + \hat{\alpha}_h + \hat{\beta}_1 \cdot t + \hat{\gamma}_h \cdot t\right)} \qquad \text{for } h \in \{1, 2, 3\} \tag{3}$$

with the estimated regression coefficients $\hat{\beta}_0$, $\hat{\alpha}_h$, $\hat{\beta}_1$, and $\hat{\gamma}_h$, where $\hat{\alpha}_1 = 0$ and $\hat{\gamma}_1 = 0$.

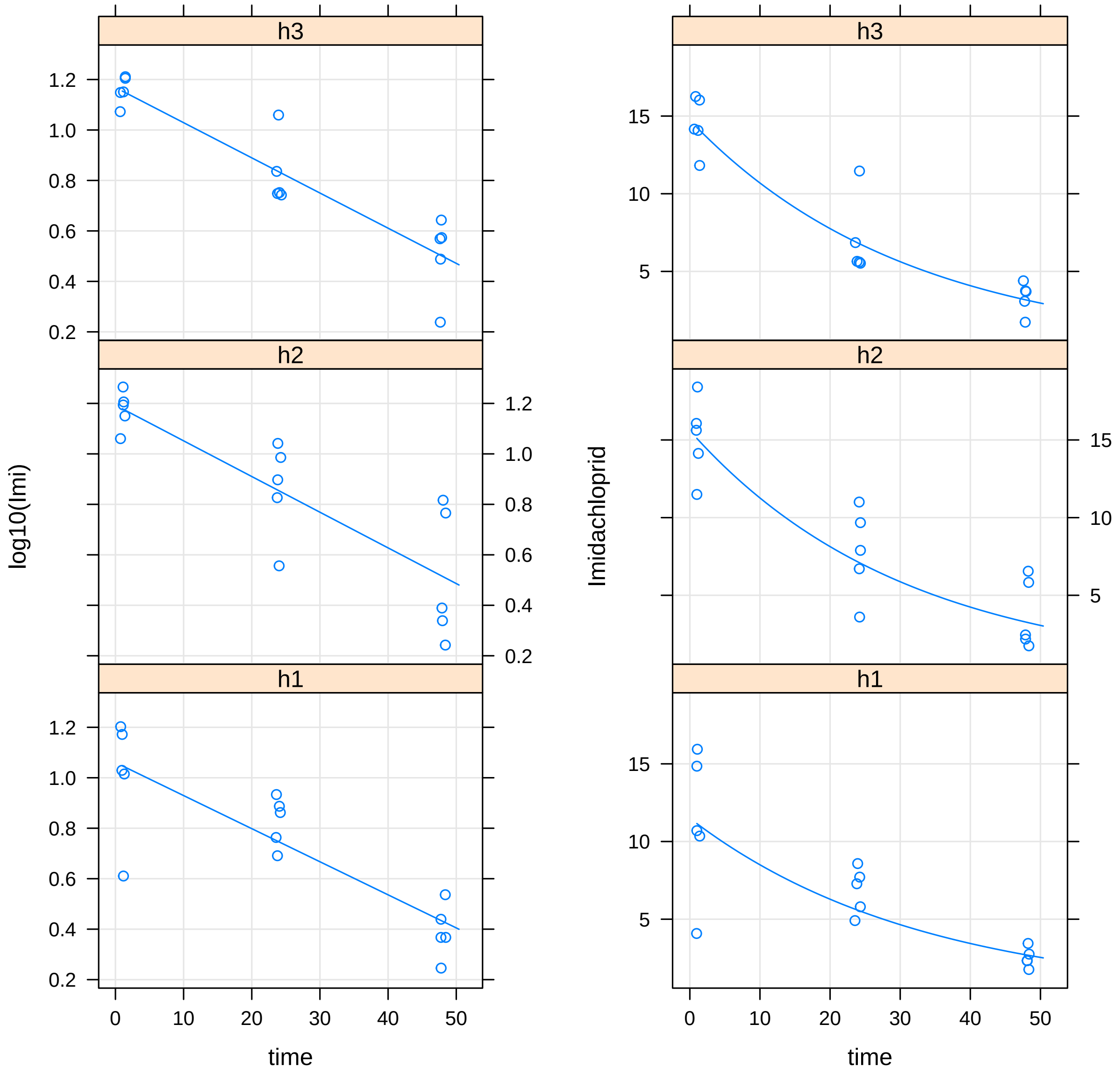


Figure 3: "Augmented prediction plots" for $\log_{10}$(Imidachloprid) vs. time by hive: left: on the $\log_{10}$-scale; right: re-transformed onto the original scale; both augmented by the raw data. (File names: left: *MS-Q1_AugmPredPlots_Imi.pdf*, right: *MS-Q1_AugmPredPlots_Imi_B.pdf*)

### Model diagnostics

Fig. 4 displays three qualitative diagnostic plots for each of the models (1) and (2): in its left column for the model with Imidachloprid on its original scale, and in its right column for the $\log_{10}$-transformed response (see column titles).

**Summary:** None of the two models indicates serious violations of the typical model assumptions of homoscedasticity and normality of errors, as can be seen in the plots of the residuals vs. the fitted values (top row) and in the normal q-q plots for the (studentized) residuals (bottom row). Also, there are no unduly influential observations (outliers) according to Cook's distances (middle row).

Comparing the multiple $R^2$-values (see `Multiple R-squared` in tables 1 and 2), which measure the goodness-of-fit of the models to the data, the non-transformed model appears to fit slightly better. However, its technical "advantage" to produce positive response values leads us to prefer the $\log_{10}$-transformed model.

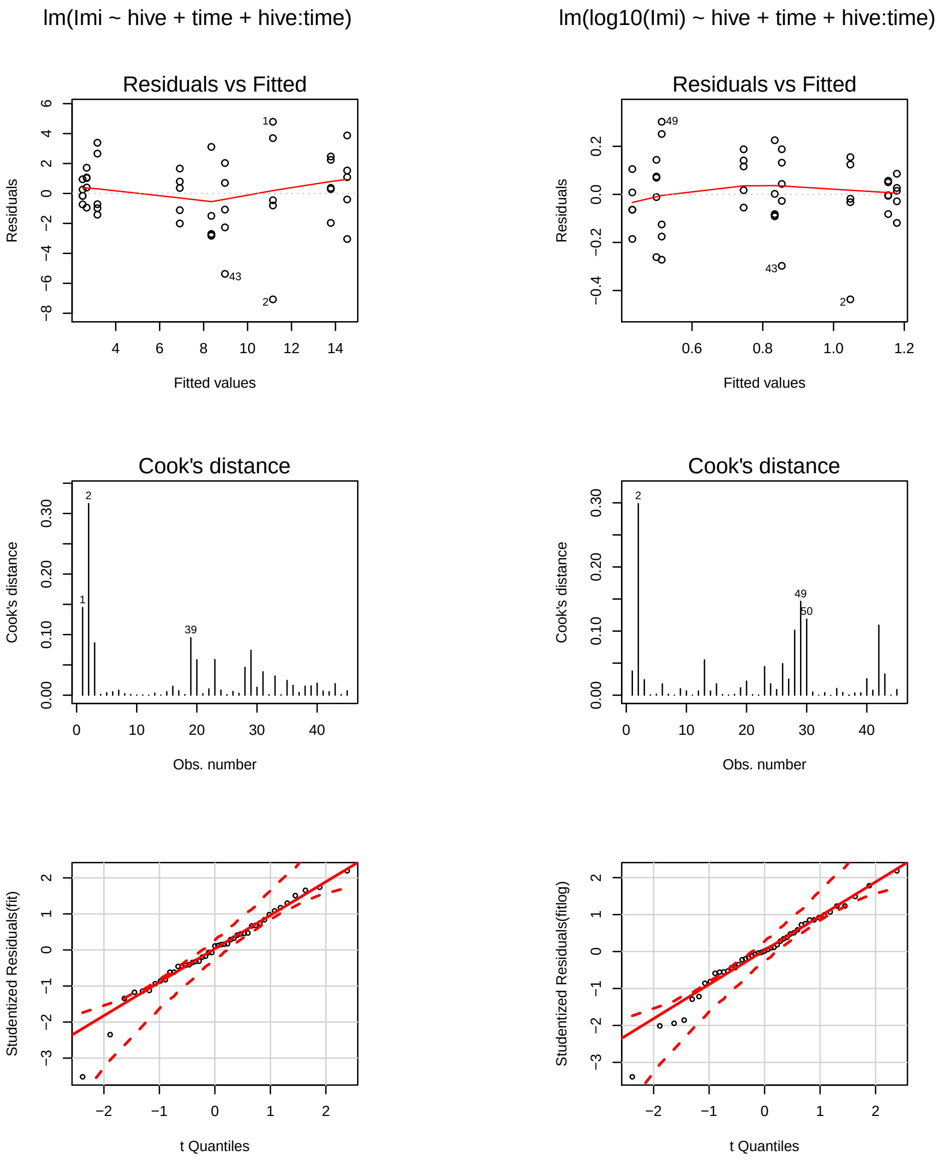


Figure 4: Diagnostic plots for the regression models of Imidachloprid on time and hive on the left, and of $\log_{10}$(Imidachloprid) on time and hive on the right. Top row: Residuals vs. fitted values; should neither show a trend (red) deviating from zero nor changing variability in the residuals along the fitted values. Middle row: Cook's distance measures the influence of each data point on the fit; should show neither any "spike" heavily towering over the others nor being larger than 1. Bottom row: Sorted (studentized) residuals vs. corresponding theoretical $t$-quantiles; should show a linear "chain of points" not too far away from the red, solid reference line and mainly within the pointwise 95 % confidence interval bounds (dashed). (File names: left: *MS-Q1_DiagPlots_Imi.pdf*, right: *MS-Q1_DiagPlots_Imi_B.pdf*)

**Statistical inference for the fitted models**

Tab. 3 presents the analysis of variance table (ANOVA table) of the so-called sequential tests (also known as "type-I tests") for the model terms of the regression model of the log-transformed response Imidachloprid on time and hive with interaction. The ANOVA table has one row per model term and the respective term is given at the beginning of the row. It contains in its last column (`Pr(>F)`) the $p$-value of the test of the hypothesis of no influence of the respective model term, given that the model already contains the terms "above" the row's term. This is the reason why the tests are called sequential. (Warning: If the design is unbalanced – which is not the case here – the results depend on the order of the terms in the model formula.)

Table 3: Sequential ("type-I tests") ANOVA table for the log-linear regression model of Imidachloprid on time and hive with interaction.

```
> anova( fitlog)

Analysis of Variance Table

Response: log10(Imi)
          Df  Sum Sq Mean Sq  F value    Pr(>F)    
hive       2 0.09815 0.04907   1.9447    0.1566    
time       1 3.12439 3.12439 123.8133 1.142e-13 ***
hive:time  2 0.00329 0.00164   0.0652    0.9370    
Residuals 39 0.98415 0.02523                       
---
Signif. codes:  0 '***' 0.001 '**' 0.01 '*' 0.05 '.' 0.1 ' ' 1
```

**Summary:** There is no significant difference ($p = 0.157$) between hives in the overall $\log_{10}$(Imidachloprid)-levels (averaged across time). After adjusting for a main effect of hive there is a significant time main effect ($p = 1.14 \times 10^{-13}$). After adjusting for the main effects of hive and time there is no significant interaction effect between hive and time ($p = 0.937$).

Consequently, we fit the simpler log-linear regression model of Imidachloprid on only time without any hive-effect (see tab. 4), and compare it with the previous, more complicated model to find out if all hive-effects are statistically negligeable simultaneously (see tab. 5):

Table 4: Regression of the transformed response variable $\log_{10}$(Imidachloprid) on time alone.

```
> summary( fitlog1 <- update( fitlog, ~ time))

Call:
lm(formula = log10(Imi) ~ time, data = BeesX)

Residuals:
     Min       1Q   Median       3Q      Max
-0.51631 -0.09242  0.02329  0.08410  0.33479

Coefficients:
             Estimate Std. Error t value Pr(>|t|)
(Intercept)  1.140702   0.038252   29.82  < 2e-16 ***
time        -0.013732   0.001234  -11.12 3.08e-14 ***
---
Signif. codes:  0 '***' 0.001 '**' 0.01 '*' 0.05 '.' 0.1 ' ' 1

Residual standard error: 0.1589 on 43 degrees of freedom
Multiple R-squared:  0.7421,	Adjusted R-squared:  0.7361
F-statistic: 123.8 on 1 and 43 DF,  p-value: 3.083e-14
```

**Remark:** The not shown qualitative diagnostic plots for the model neither indicate violations of the typical model assumptions of homoscedasticity and normality of errors, neither appear overly influential observations (outliers). The slightly smaller multiple $R^2$-value is nearly fully compensated by the simplicity of this model.

Table 5: ANOVA table for the comparison of the log-linear regression model of Imidachloprid on only time, i.e., without any hive-effect, (`Model 1`) with the model on time and hive with interaction (`Model 2`).

```
> anova( fitlog1, fitlog)

Analysis of Variance Table

Model 1: log10(Imi) ~ time
Model 2: log10(Imi) ~ hive + time + hive:time
  Res.Df     RSS Df Sum of Sq     F Pr(>F)
1     43 1.08559
2     39 0.98415  4   0.10144 1.005 0.4167
```

**Summary:** There is no significant difference ($p = 0.417$) between the two models. This means that the terms in which the two models differ provide no significant contribution to the distributional "behaviour" of Imidachloprid, i.e., no hive-effect is statistically significant. So, we end up with the following model:

*Short formal representation of the model in tab. 4:*
For hive $h \in \{1, 2, 3\}$ in bee-group $i \in \{1, \ldots, 5\}$ the observed residue level at time $t_j$ with $j \in \{1, 2, 3\}$ is denoted $Y_{hi}(t_j)$ and modelled as:

$$\log_{10}(Y_{hi}(t_j)) = \beta_0 + \beta_1 \cdot t_j + e_{hij} \tag{4}$$

Note: the regression function now does (of course) *not* depend on hive!

So, the re-transformed estimated regression function reads:

$$\hat{y}(t) = 10^{\left(\hat{\beta}_0 + \hat{\beta}_1 \cdot t\right)} \tag{5}$$

with the estimated regression coefficients $\hat{\beta}_0$ and $\hat{\beta}_1$, whose values are provided in the rows `(Intercept)` and `time`, respectively, of the `Estimate`-column of the `Coefficients`-table in the corresponding R-output in tab. 4. In addition, tab. 6 presents the estimated regression coefficients together with their lower and upper confidence limits for a confidence level of 95 %.

### Some additional considerations and visualizations

Table 6: Estimated regression coefficients of model (4) with lower and upper bounds (labelled 2.5 % and 97.5 %, respectively) of their 95 %-confidence intervals in the log-linear regression model of Imidachloprid on time.

```
> est.and.ci <- cbind( Estimate = coef( fitlog1), confint( fitlog1, level = 1 - alpha))
> signif( est.and.ci[, c( 2, 1, 3)], 4)    # for a nicer output
```

```
                2.5 % Estimate   97.5 %
(Intercept)  1.06400  1.14100  1.21800
time        -0.01622 -0.01373 -0.01124
```

**Remark:** From the re-transformed fitted model in (5) we deduce that for any two time points which are $\Delta$ time units apart the ratio $q$ of their pertaining Imidachloprid concentrations is estimated by

$$q \equiv \frac{\hat{y}(t+\Delta)}{\hat{y}(t)} = 10^{\hat{\beta}_1 \cdot \Delta} = 10^{-0.01373 \cdot \Delta} \tag{6}$$

So, for a given relative change $q$ in the Imidachloprid concentration the required time to reach it can be estimated by

$$\hat{\Delta} := \frac{\log_{10}(q)}{\hat{\beta}_1} = \frac{\log_{10}(q)}{-0.01373} \tag{7}$$

For example, the time required to reach a reduction to 50 % of any original Imidachloprid concentration, i.e., the half time, is estimated to be $\log_{10}(0.5)/\hat{\beta}_1 \approx 22$ time units, here hours (with a 95 %-confidence interval of $[18.6, 26.8]$). After estimated $\log_{10}(0.1)/\hat{\beta}_1 \approx 73$ time units (with a 95 %-confidence interval of $[61.6, 88.9]$) only 10 % of any original Imidachloprid concentration is left.

For the sake of completeness visualizes fig. 5 in its left part the estimated regression line of the fitted simple log-linear model (5), augmented by the raw data (as they are already presented in fig. 2 on the right, but here without color-coding the now ignored hives). In its right part, fig. 5 shows the respective estimated regression function re-transformed onto the original scale (also augmented by the raw data as they are seen in fig. 2 on the left).

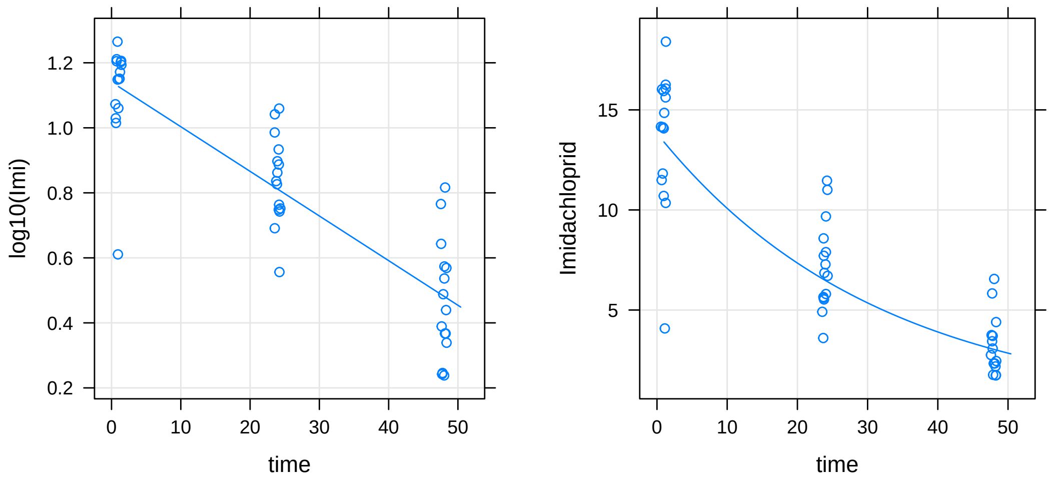


Figure 5: Augmented prediction plots for $\log_{10}$(Imidachloprid) vs. time: left: on the $\log_{10}$-scale; right: re-transformed onto the original scale; both augmented by the raw data. (File names: left: *MS-Q1_AugmPredPlots2_Imi.pdf*, right: *MS-Q1_AugmPredPlots2_Imi_B.pdf*)

Preparation:

```
> Response <- c( "5-hydroxyimidacloprid" = "Hyd")
```

### 2.1.2 5-hydroxyimidacloprid

Question: Does 5-hydroxyimidacloprid degrade in dead bees at room temperature?

#### Graphical EDA

Figures 6 and 7 show exploratory displays analogous to figures 1 and 2. For technical details confer with the beginning of section 2.1.1 on page 8, and regarding the R code that creates the following respective figures or regression models see the corresponding parts in section 2.1.1.

**Remark:** Fig. 7 indicates that a transformation appears to be unnecessary here, and, in particular, a log-transformation even counterproductive.

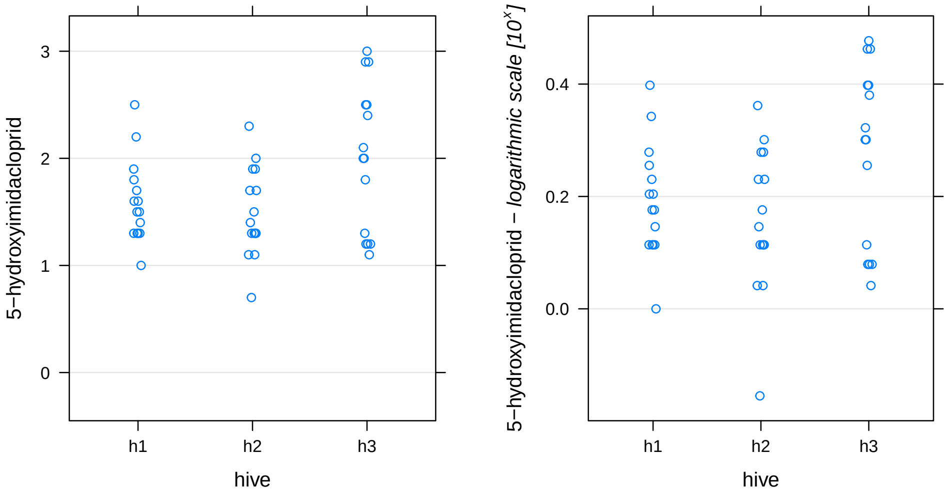


Figure 6: 5-hydroxyimidacloprid by hive, for explanations see caption of fig. 1. (File names: left: *MS-Q1_EDA_Hyd_by_hive_NOLOG.pdf*, right: *MS-Q1_EDA_Hyd_by_hive_LOG.pdf*)

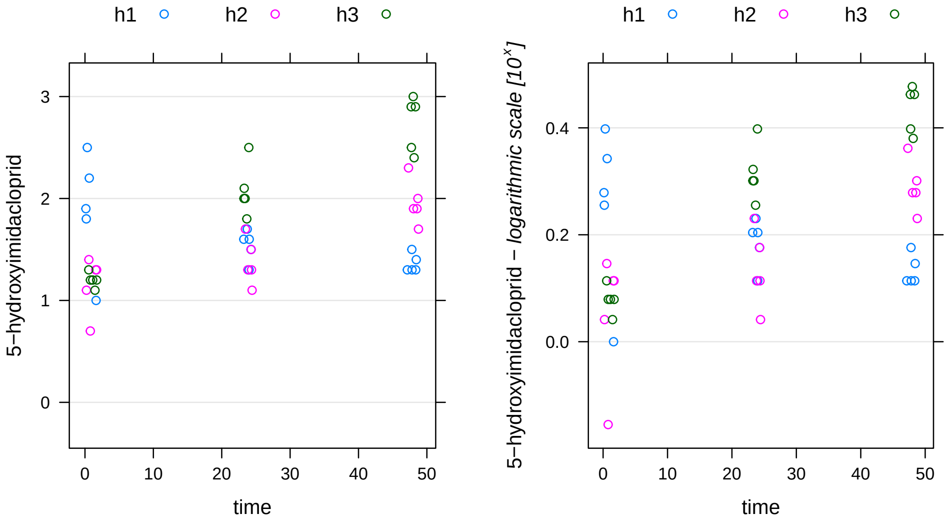


Figure 7: 5-hydroxyimidacloprid (left on original scale, right on $\log_{10}$-scale) vs. time with hive-specific plotting colors (horizontally jittered; for explanations see caption of fig. 1 and for details see text there). (File names: left: *MS-Q1_EDA_Hyd_vs_time_NOLOG.pdf*, right: *MS-Q1_EDA_Hyd_vs_time_LOG.pdf*)

### Model fitting & model diagnostics

Tables 7 and 8 contain the linear and the log-linear regression model, respectively, of 5-hydroxyimidacloprid on time and hive with interaction, details for which are given on pages 10 and 11, and whose corresponding short formal representations are identical to those given in (1) and (2), respectively. Comparison of the goodness-of-fit measure multiple $R^2$ for the models (`Multiple R-squared` in tables 7 and 8) suggests to prefer the *not*-transformed model of tab. 7 (regardless of the technical advantage of the log-linear model to produce positive response values). In addition, the qualitative diagnostic plots in fig. 8 for each of the models (left: for the untransformed model; right: for the model with $\log_{10}$-transformed response) give also reason to prefer the untransformed model because its normal q-q plot for the (studentized) residuals (bottom row) looks better than the one for the log-linear model, while the plots of the residuals vs. the fitted values (top row) and the Cook-distances (bottom row) do not present noteable differences between the two models.

**Summary:** The linear regression model of (untransformed) 5-hydroxyimidacloprid on time and hive with interaction appears to describe the trend in 5-hydroxyimidacloprid along time well enough over the time-range observed (so that a log-linear transformation seems not necessary here).

Fig. 9 visualizes the hive-specific estimated regression lines of the fitted model in one panel per hive, augmented by the raw data (as they are already presented in fig. 7 on the left).

Table 7: Regression of 5-hydroxyimidacloprid on time and hive with interaction.

```
>    # 'building' the model formula using variables (for the sake of flexibility):
> form <- formula( paste( Response, "~", Group, "*", ContCovar))
> fit <- lm( form, data = BeesX)  # fitting the linear model.
> fit <- update( fit, ~ .)  # use of update() here is just a trick to see the model
>                           # formula in evaluated form in the following outputs.
> summary( fit)

Call:
lm(formula = Hyd ~ hive + time + hive:time, data = BeesX)

Residuals:
     Min       1Q   Median       3Q      Max
-0.85089 -0.09701  0.00299  0.11877  0.64911

Coefficients:
             Estimate Std. Error t value Pr(>|t|)
(Intercept)  1.861925   0.112910  16.490  < 2e-16 ***
hiveh2      -0.777369   0.159679  -4.868 1.90e-05 ***
hiveh3      -0.651635   0.159679  -4.081 0.000215 ***
time        -0.011038   0.003644  -3.029 0.004332 **
hiveh2:time  0.028111   0.005153   5.456 2.95e-06 ***
hiveh3:time  0.043766   0.005153   8.494 2.10e-10 ***
---
Signif. codes:  0 '***' 0.001 '**' 0.01 '*' 0.05 '.' 0.1 ' ' 1

Residual standard error: 0.2708 on 39 degrees of freedom
Multiple R-squared:  0.784,	Adjusted R-squared:  0.7563
F-statistic: 28.31 on 5 and 39 DF,  p-value: 5.287e-12
```

Table 8: Regression of $\log_{10}$(5-hydroxyimidacloprid) on time and hive with interaction.

```
Call:
lm(formula = log10(Hyd) ~ hive + time + hive:time, data = BeesX)

Residuals:
      Min        1Q    Median        3Q       Max
-0.251679 -0.020500  0.006158  0.027075  0.146261

Coefficients:
             Estimate Std. Error t value Pr(>|t|)
(Intercept)  0.254273   0.032138   7.912 1.24e-09 ***
hiveh2      -0.218624   0.045450  -4.810 2.28e-05 ***
hiveh3      -0.162179   0.045450  -3.568 0.000971 ***
time        -0.002594   0.001037  -2.502 0.016668 *
hiveh2:time  0.007669   0.001467   5.229 6.06e-06 ***
hiveh3:time  0.010181   0.001467   6.942 2.58e-08 ***
---
Signif. codes:  0 '***' 0.001 '**' 0.01 '*' 0.05 '.' 0.1 ' ' 1

Residual standard error: 0.07707 on 39 degrees of freedom
Multiple R-squared:  0.7241,	Adjusted R-squared:  0.6888
F-statistic: 20.47 on 5 and 39 DF,  p-value: 5.588e-10
```

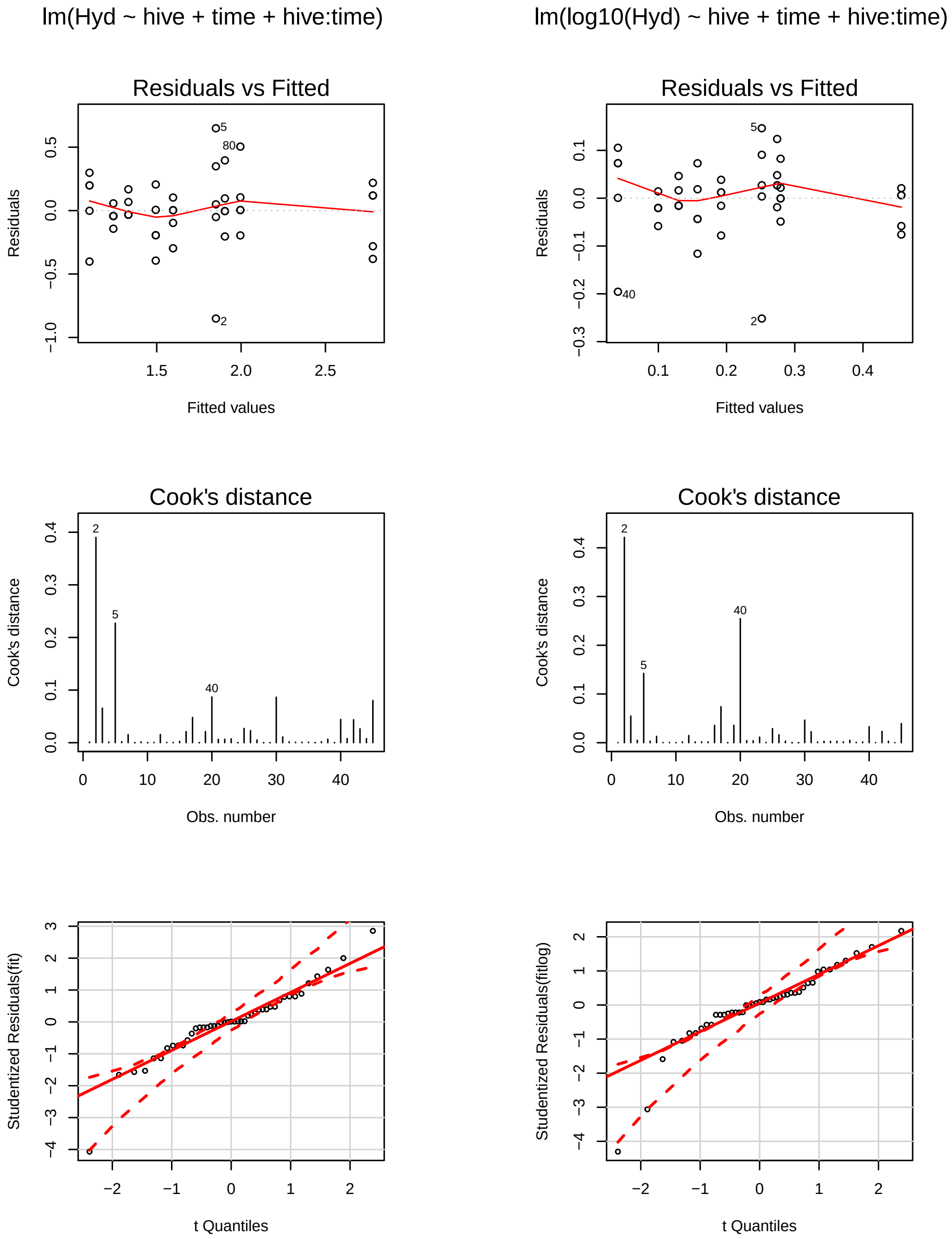


Figure 8: Diagnostic plots for the regression models of 5-hydroxyimidacloprid on time and hive on the left, and of $\log_{10}$(5-hydroxyimidacloprid) on time and hive on the right. (For a few technical explanations see caption of fig. 4.) (File names: left: *MS-Q1_DiagPlots_Hyd.pdf*, right: *MS-Q1_DiagPlots_Hyd_B.pdf*)

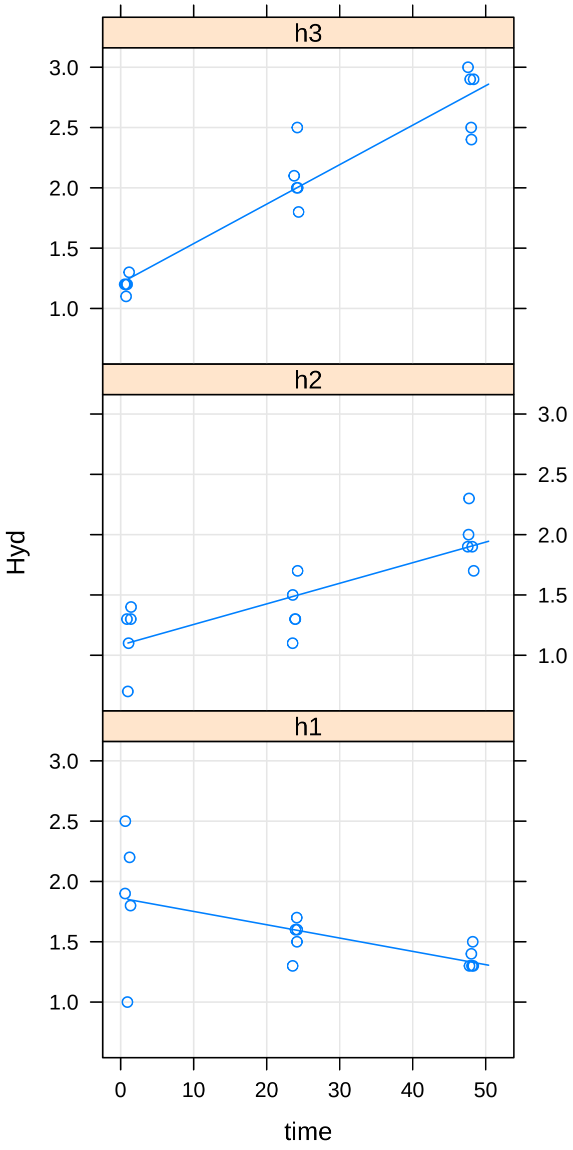


Figure 9: Augmented prediction plots for 5-hydroxyimidacloprid vs. time by hive, augmented by the raw data. (File name: *MS-Q1_AugmPredPlots_Hyd.pdf*

### Statistical inference for the fitted models

Tab. 9 presents the ANOVA table of the sequential tests for the terms of the regression model in tab. 7 of 5-hydroxyimidacloprid on time and hive with interaction. For details on structure and interpretation of the ANOVA table see the explanations regarding tab. 3.

Table 9: Sequential ("type-I tests") ANOVA table for the regression model of 5-hydroxyimidacloprid on time and hive with interaction.

```
> anova( fit)

Analysis of Variance Table

Response: Hyd
          Df Sum Sq Mean Sq F value    Pr(>F)
hive       2 2.1813 1.09067  14.875 1.581e-05 ***
time       1 2.7664 2.76637  37.728 3.285e-07 ***
hive:time  2 5.4327 2.71633  37.046 9.636e-10 ***
Residuals 39 2.8596 0.07332
---
Signif. codes:  0 '***' 0.001 '**' 0.01 '*' 0.05 '.' 0.1 ' ' 1
```

**Summary:** There is a significant difference ($p = 1.58 \times 10^{-5}$) between hives in the overall 5-hydroxyimidacloprid-levels (averaged across time). After adjusting for a main effect of hive there is a significant time main effect ($p = 3.29 \times 10^{-7}$). After adjusting for the main effects of hive and time there is a significant interaction effect between hive and time ($p = 9.64 \times 10^{-10}$). (The latter significance, in turn, means that each main effect hypothesis has to be interpreted on average across the levels/values of the respective other factor/variable.)

Tab. 10 presents the estimated regression coefficients of the model in tab. 7 together with their lower and upper confidence limits for a confidence level of 95 %.

Table 10: Estimated regression coefficients with lower and upper bounds (labelled 2.5 % and 97.5 %, respectively) of their 95 %-confidence intervals in the linear regression model of 5-hydroxyimidacloprid on time and hive (analogue to model (1)).

```
> est.and.ci <- cbind( Estimate = coef( fit), confint( fit, level = 1 - alpha))
> signif( est.and.ci[, c( 2, 1, 3)], 4)   # for a nicer output

                2.5 % Estimate    97.5 %
(Intercept)  1.63400  1.86200  2.090000
hiveh2      -1.10000 -0.77740 -0.454400
hiveh3      -0.97460 -0.65160 -0.328700
time        -0.01841 -0.01104 -0.003668
hiveh2:time  0.01769  0.02811  0.038530
hiveh3:time  0.03334  0.04377  0.054190
```

Preparation:

```
> Response <- c( "Oelefin" = "Oel")
```

### 2.1.3 Oelefin

Question: Does Oelefin degrade in dead bees at room temperature?

#### Graphical EDA

Figures 10 and 11 show exploratory displays analogous to figures 1 and 2. For technical details confer with the beginning of section 2.1.1 on page 8, and regarding the R code that creates the following respective figures or regression models see the corresponding parts in section 2.1.1.

**Remark:** Fig. 11 indicates that a transformation appears to be unnecessary here, and, in particular, a log-transformation even counterproductive.

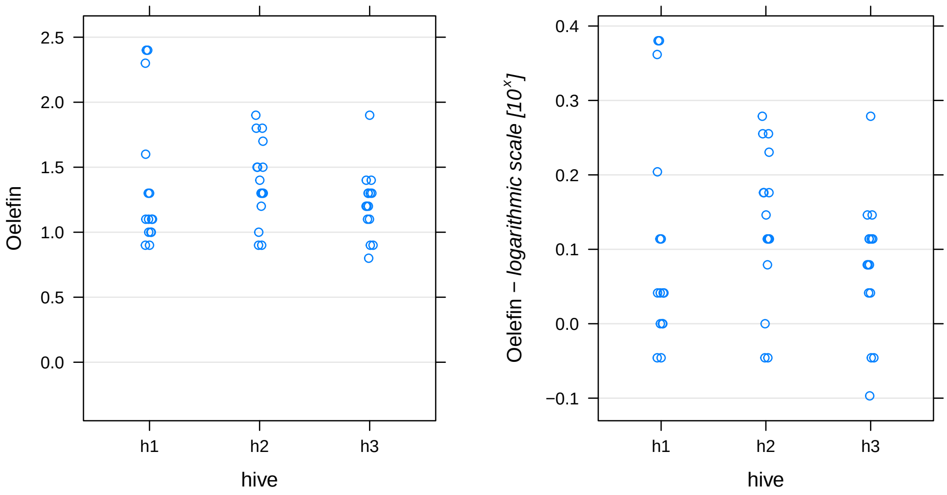


Figure 10: Oelefin by hive, for explanations see caption of fig. 1. (File names: left: *MS-Q1_EDA_Oel_by_hive_NOLOG.pdf*, right: *MS-Q1_EDA_Oel_by_hive_LOG.pdf*)

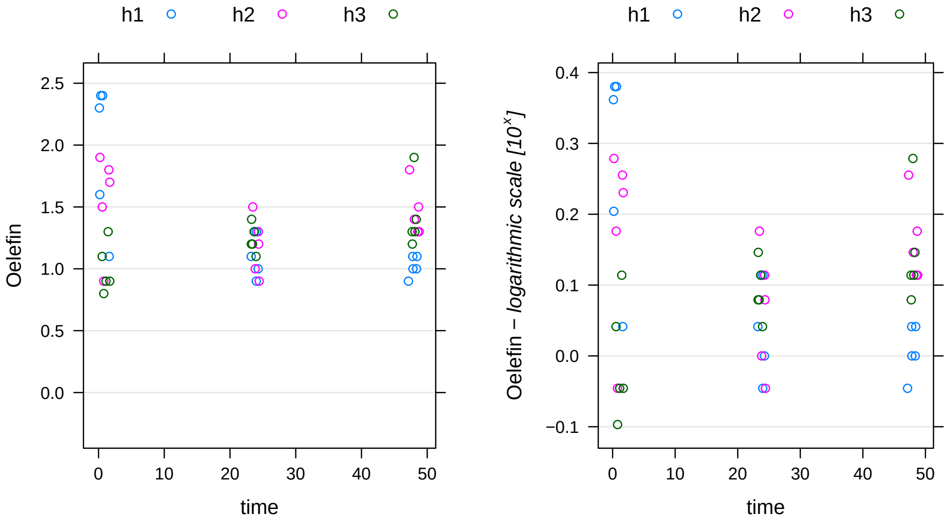


Figure 11: Oelefin (left on original scale, right on $\log_{10}$-scale) vs. time with hive-specific plotting colors (horizontally jittered; for explanations see caption of fig. 1 and for details see text there). (File names: left: *MS-Q1_EDA_Oel_vs_time_NOLOG.pdf*, right: *MS-Q1_EDA_Oel_vs_time_LOG.pdf*)

**Model fitting & model diagnostics**

Tables 11 and 12 contain the linear and the log-linear regression model, respectively, of Oelefin on time and hive with interaction, details for which are given on pages 10 and 11, and whose corresponding short formal representations are identical to those given in (1) and (2), respectively. Comparison of the goodness-of-fit measure multiple $R^2$ for the models (`Multiple R-squared` in tables 11 and 12) suggests a slightly worse fit of the log-transformed model of tab. 12. But, the technical advantage of the log-linear model to produce positive response values in combination with the qualitative diagnostic plots in fig. 12 for each of the models (left: for the untransformed model; right: for the model with $\log_{10}$-transformed response) give reason to prefer the transformed log-linear model: its diagnostic plots look a little better than the ones for the untransformed model.

**Summary:** The log-linear regression model of Oelefin on time and hive with interaction appears to describe the hive-specific trends in Oelefin along time well enough over the time-range observed.

Fig. 13 visualizes the hive-specific estimated regression lines of the fitted model in one panel per hive, augmented by the raw data (as they are already presented in fig. 11 on the left).

Table 11: Regression of Oelefin on time and hive with interaction.

```
>    # 'building' the model formula using variables (for the sake of flexibility):
> form <- formula( paste( Response, "~", Group, "*", ContCovar))
> fit <- lm( form, data = BeesX)  # fitting the linear model.
> fit <- update( fit, ~ .)  # use of update() here is just a trick to see the model
>                           # formula in evaluated form in the following outputs.
> summary( fit)

Call:
lm(formula = Oel ~ hive + time + hive:time, data = BeesX)

Residuals:
     Min       1Q   Median       3Q      Max 
-0.73066 -0.13124 -0.01702  0.18298  0.56934 

Coefficients:
             Estimate Std. Error t value Pr(>|t|)    
(Intercept)  1.850543   0.132374  13.980  < 2e-16 ***
hiveh2      -0.401201   0.187205  -2.143  0.03840 *  
hiveh3      -0.847737   0.187205  -4.528 5.48e-05 ***
time        -0.019885   0.004272  -4.655 3.69e-05 ***
hiveh2:time  0.017858   0.006041   2.956  0.00527 ** 
hiveh3:time  0.028811   0.006041   4.769 2.59e-05 ***
---
Signif. codes:  0 '***' 0.001 '**' 0.01 '*' 0.05 '.' 0.1 ' ' 1

Residual standard error: 0.3175 on 39 degrees of freedom
Multiple R-squared:  0.4264,	Adjusted R-squared:  0.3529 
F-statistic: 5.799 on 5 and 39 DF,  p-value: 0.0004278
```

Table 12: Regression of $\log_{10}$(Oelefin) on time and hive with interaction.

```
Call:
lm(formula = log10(Oel) ~ hive + time + hive:time, data = BeesX)

Residuals:
      Min        1Q    Median        3Q       Max
-0.198580 -0.047209  0.003018  0.066148  0.140238

Coefficients:
             Estimate Std. Error t value Pr(>|t|)
(Intercept)  0.245606   0.039966   6.145 3.25e-07 ***
hiveh2      -0.102211   0.056521  -1.808  0.07826 .
hiveh3      -0.247403   0.056521  -4.377 8.73e-05 ***
time        -0.005633   0.001290  -4.367 8.99e-05 ***
hiveh2:time  0.005284   0.001824   2.897  0.00615 **
hiveh3:time  0.008881   0.001824   4.869 1.89e-05 ***
---
Signif. codes:  0 '***' 0.001 '**' 0.01 '*' 0.05 '.' 0.1 ' ' 1

Residual standard error: 0.09585 on 39 degrees of freedom
Multiple R-squared:  0.4197,	Adjusted R-squared:  0.3454
F-statistic: 5.642 on 5 and 39 DF,  p-value: 0.0005247
```

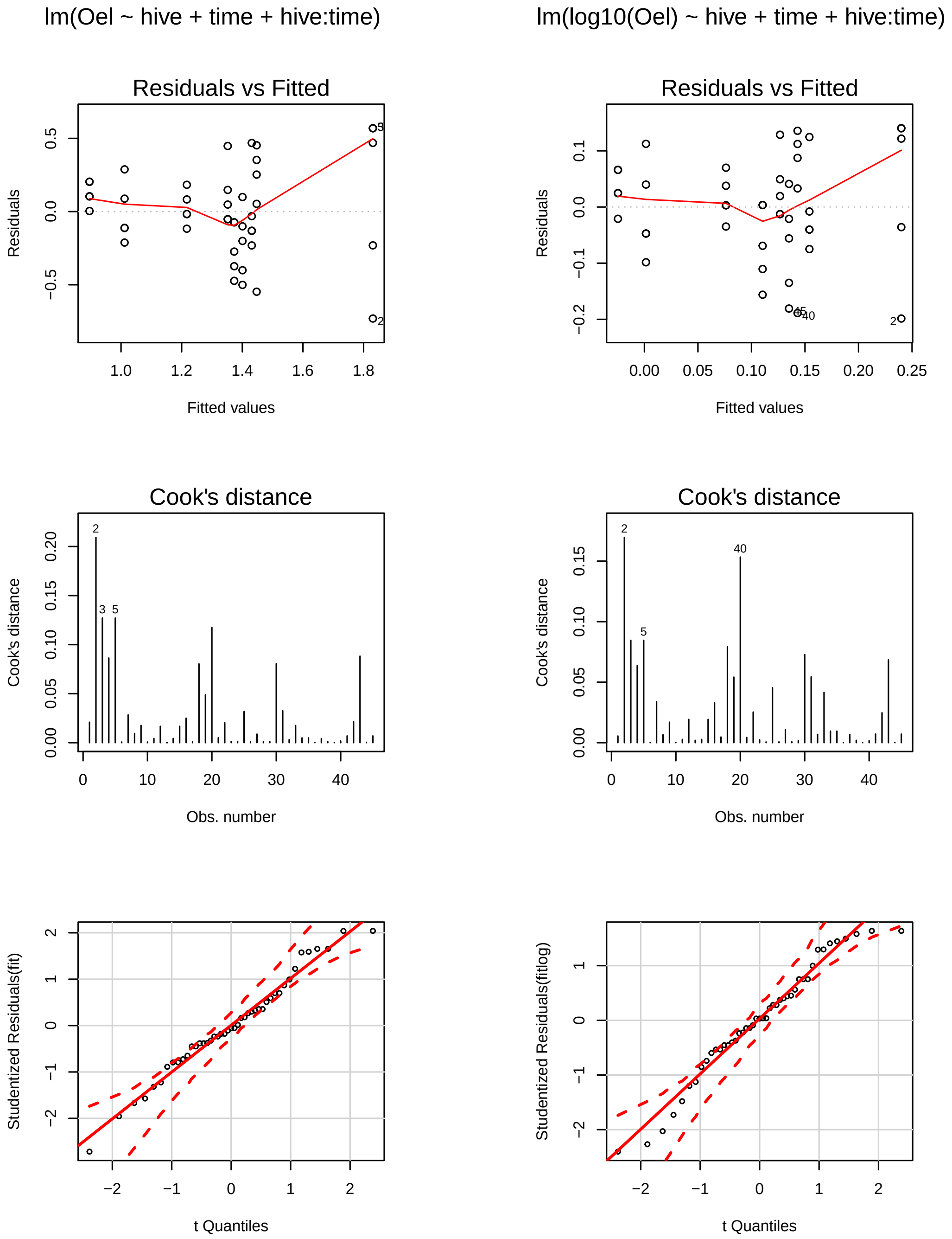


Figure 12: Diagnostic plots for the regression models of Oelefin on time and hive on the left, and of $\log_{10}$(Oelefin) on time and hive on the right. (For a few technical explanations see caption of fig. 4.) (File names: left: *MS-Q1_DiagPlots_Oel.pdf*, right: *MS-Q1_DiagPlots_Oel_B.pdf*)

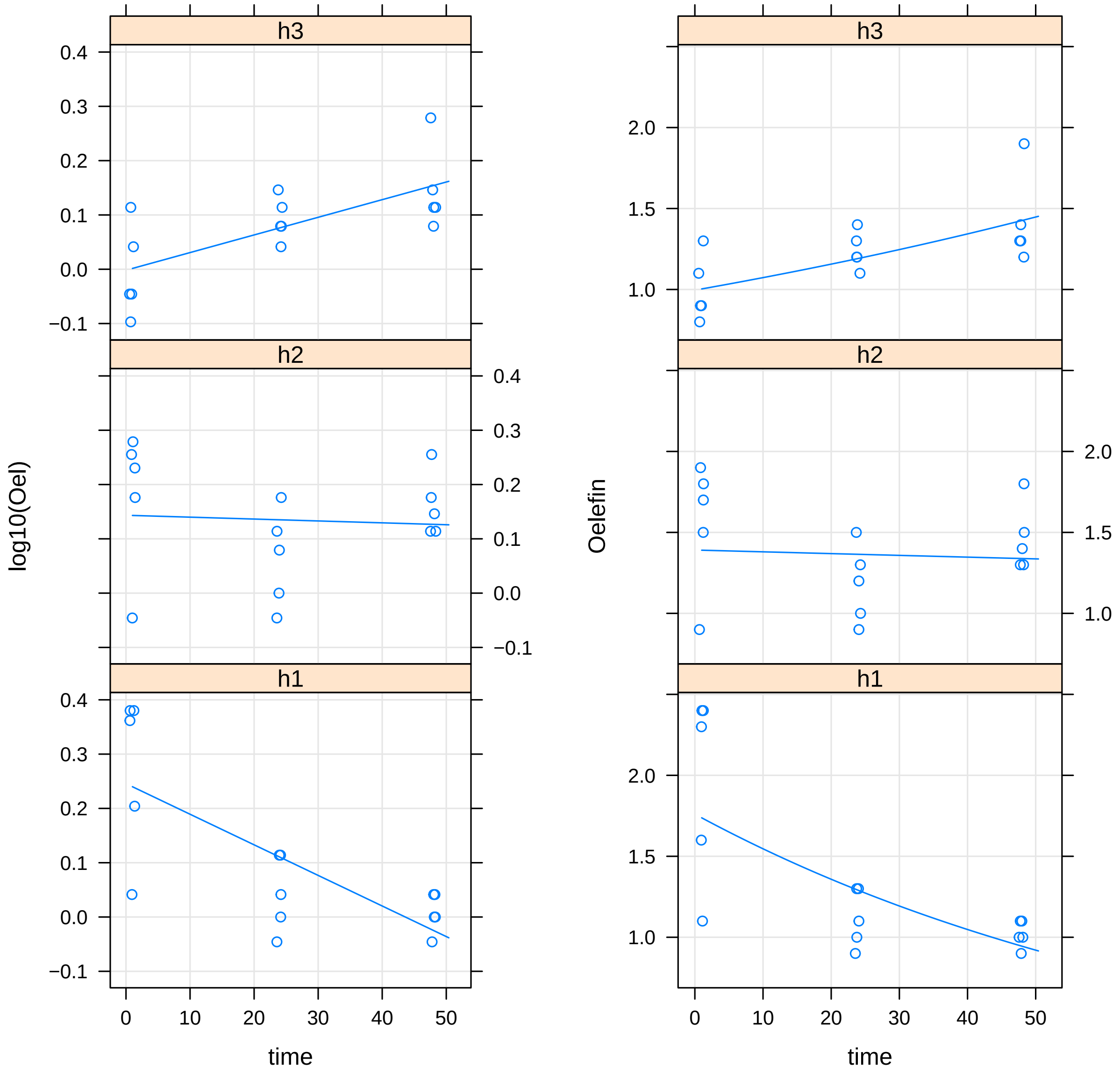


Figure 13: Augmented prediction plots for Oelefin vs. time by hive, augmented by the raw data. (File name: left: *MS-Q1_AugmPredPlots_Oel.pdf* right: *MS-Q1_AugmPredPlots_Oel_B.pdf*)

**Statistical inference for the fitted models**

Table 13 presents the ANOVA table of sequential tests (aka "type-I tests") for the terms of the regression model. It shows the sequential tests for the log-linear model of Oelefin on time and hive with interaction. (For details of the structure of the ANOVA table see the explanations regarding tab. 3.)

**Summary:** There is no significant difference ($p = 0.268$) between hives in the overall $\log_{10}$(Oelefin)-levels (averaged across time). After adjusting for a main effect of hive there is no significant time main effect ($p = 0.229$). After adjusting for the main effects of hive and time there is a significant interaction effect between hive and time ($p = 8.7 \times 10^{-5}$). (The latter significance, in turn, means that each main effect hypothesis has to be interpreted on average across the levels/values of the respective other factor/variable.)

Tab. 14 presents the estimated regression coefficients of the model in tab. 12 together with their lower and upper confidence limits for a confidence level of 95 %.

Table 13: Sequential ("type-I tests") ANOVA table for the log-linear regression model of Oelefin on time and hive with interaction.

```
> anova( fitlog)

Analysis of Variance Table

Response: log10(Oel)
          Df  Sum Sq  Mean Sq F value    Pr(>F)
hive       2 0.02500 0.012500  1.3606    0.2684
time       1 0.01375 0.013751  1.4968    0.2285
hive:time  2 0.22043 0.110215 11.9971 8.698e-05 ***
Residuals 39 0.35829 0.009187
---
Signif. codes:  0 '***' 0.001 '**' 0.01 '*' 0.05 '.' 0.1 ' ' 1
```

Table 14: Estimated regression coefficients with lower and upper bounds (labelled 2.5 % and 97.5 %, respectively) of their 95 %-confidence intervals in the log-linear regression model of Oelefin on time and hive (analogue to model (2)).

```
> est.and.ci <- cbind( Estimate = coef( fitlog), confint( fitlog, level = 1-alpha))
> signif( est.and.ci[, c( 2, 1, 3)], 4)   # for a nicer output

                2.5 %  Estimate    97.5 %
(Intercept)  0.164800  0.245600  0.326400
hiveh2      -0.216500 -0.102200  0.012110
hiveh3      -0.361700 -0.247400 -0.133100
time        -0.008241 -0.005633 -0.003024
hiveh2:time  0.001595  0.005284  0.008973
hiveh3:time  0.005192  0.008881  0.012570
```

## 2.2 Question 2: Short freezing before mailing

Can the process of degradation be slowed down by short freezing?

Preparations:

```
> lev.of.int <- c( "RT1", "RT24", "RT48", "RT1mail")   # treatment levels of interest
> reflev <- "RT1mail"   # reference level for, e.g., multiple comparisons
```

1. Extracting the rows of `Bees`, whose corresponding elements in `method` are in the set {"RT1", "RT24", "RT48", "RT1mail"}, i.e., which belong to the treatments RT1, RT24, RT48 or RT1mail, and storing the "extract" in a new data frame named `BOMail`:

   ```
   > BOMail <- droplevels( subset( Bees, subset = method %in% lev.of.int))
   ```

2. Changing `method`'s level order, so that `RT1mail` is the first level and hence the reference level in later analyses (like multiple comparisons):

   ```
   > BOMail$method <- relevel( BOMail$method, ref = reflev)
   ```

3. Generating variables containing some "values" which will be needed and repeatedly (!) used in the following paragraphs, so that flexible and passably efficiently modifiable code can be designed, which is also simply reusable for subsequent paragraphs:

   ```
   > Response <- c( Imidachloprid = "Imi");   Treatment <- "method";   Group <- "hive"
   > WorkData <- BOMail
   ```

### 2.2.1 Imidachloprid

Question, stated a bit more precisely: Can the process of degradation of Imidachloprid be slowed down by short freezing in comparison to the "normal" process of degradation?

Even more precisely: Is the concentrarion of Imidachloprid in treatment-method RT1mail higher than in the other three?

To this end: Two-factorial ANOVA of Imidachloprid on the treatment (in the following called method) and on hive (with interaction) with subsequent "multiple comparisons with a control" (i.e., with a reference level) and calculation of simultaneous confidence intervals for the respective differences.

Points to consider: Repeated measurements on the same hive may induce dependencies within each group of measurements from the same hive, so analoguos remarks apply as in the "points to consider" on page 8.

#### Graphical EDA

Fig. 14 shows strip plots of Imidachloprid values on the original scale (left) and on the decimal logarithmic scale (right), grouped by method and color-coded for all hives overlaid. For a few more details about this sort of presentation see the explanations for figures 1 and 2, and recall the remark on logarithmic transformations on 8.

Fig. 15 shows in principle the same, but uses for each hive a separate panel and is amended with indications of the mean values for all method-groups which are joined by straight lines (just) for the purpose of visualization.

The latter can also be interpreted as a so-called interaction plot to assess if an interaction may be present between hive and method. Indication for interaction would be given if the hive-specific "profiles" of line segments "along" method were *not* parallel. This is not strong enough the case here, esp. in view of the variability of the raw data around their means, and will be confirmed by a formal test yielding a non-significant interaction effect.

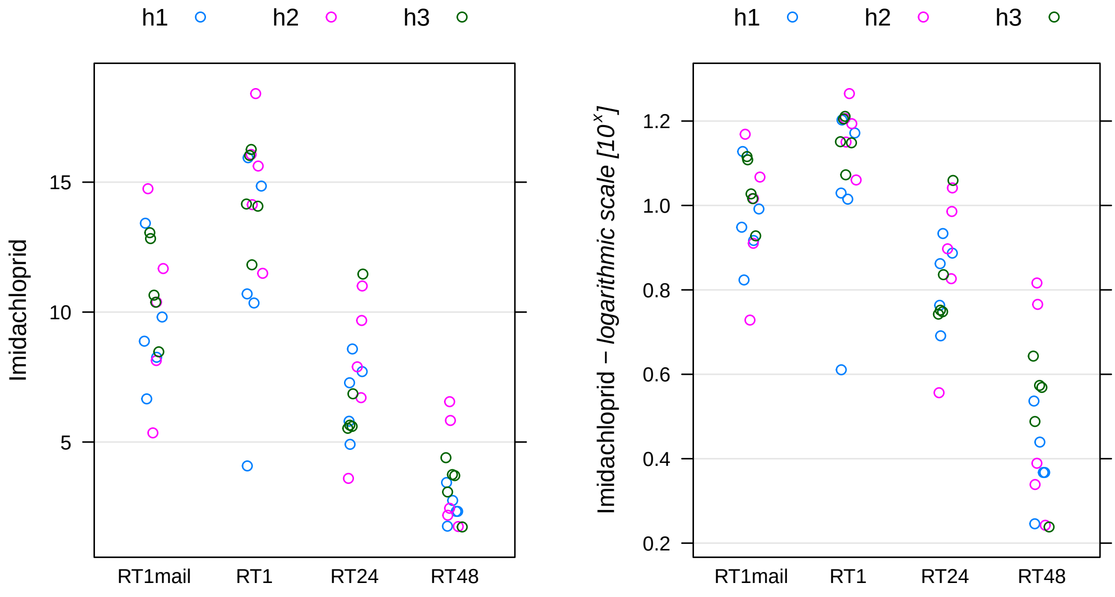


Figure 14: Imidachloprid (left on original scale, right on $\log_{10}$-scale) by method with hive-specific plotting colors (horizontally jittered; for explanations see caption of fig. 1 and for details see text there). (File names: left: *MS-Q2_EDA_Imi_by_hive_1_NOLOG.pdf*, right: *MS-Q2_EDA_Imi_by_hive_1_LOG.pdf*)

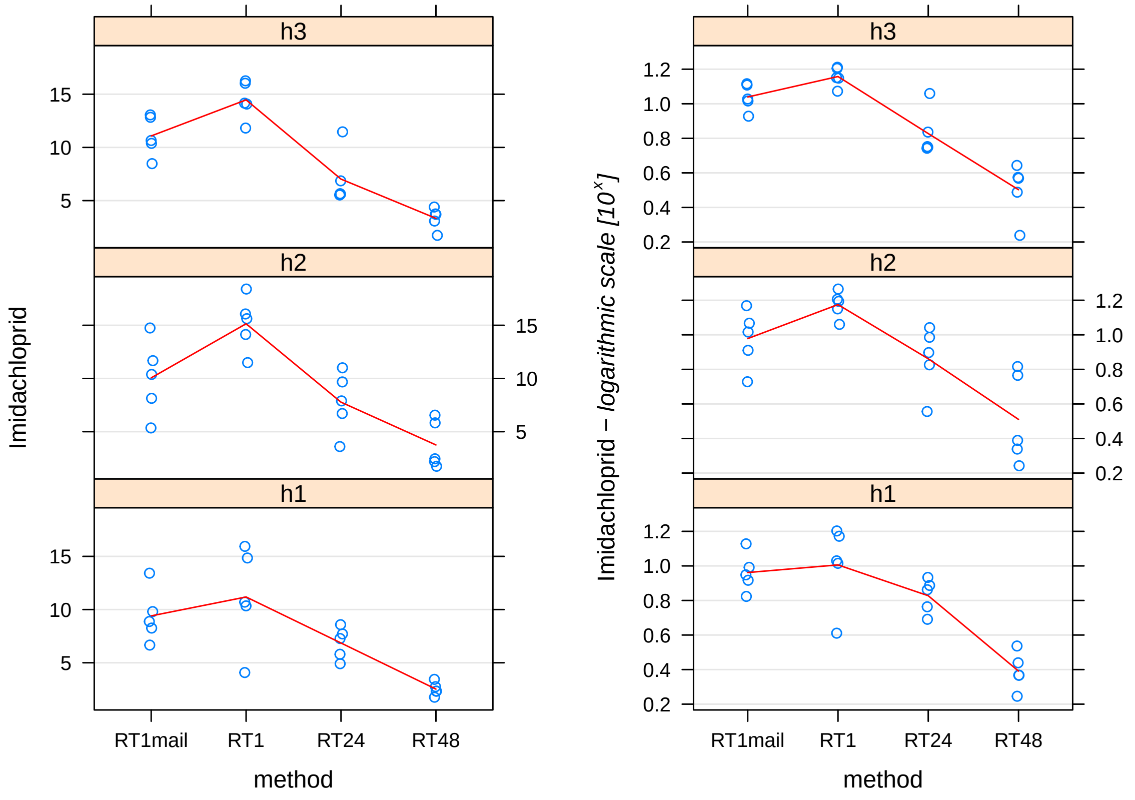


Figure 15: Imidachloprid (left on original scale, right on $\log_{10}$-scale) by method per hive with method-specific averages joined by line segments (interaction profiles (red)). (File names: left: *MS-Q2_EDA_Imi_by_hive_2_NOLOG.pdf* right: *MS-Q2_EDA_Imi_by_hive_2_LOG.pdf*)

### Model fitting & model diagnostics

Tab. 15 presents the two-factorial analysis of variance (ANOVA) table of Imidachloprid on method and hive with interaction using sequential tests (aka "type-I tests") for the model terms. For details on structure and interpretation of the ANOVA table see the explanations regarding tab. 3.

Table 15: Sequential ("type-I tests") two-factorial ANOVA of Imidachloprid on method and hive with interaction between method and hive. (`update()` is used here for the same reason as in tab. 1.)

```
>    # Orthogonal contrasts to increase numerical and statistical "stability"
> oc <- options( contrasts = c( "contr.helmert", "contr.poly"))

>    # 'Building' the model formula using variables (for the sake of flexibility):
> form <- formula( paste( Response, "~", Group, "*", Treatment))
> fit <- aov( form, data = WorkData)   # Fitting the underlying linear model.
> fit <- update( fit, ~ .)
> anova( fit)

Analysis of Variance Table

Response: Imi
            Df Sum Sq Mean Sq F value    Pr(>F)    
hive         2  33.98  16.990  2.6298   0.08246 .  
method       3 877.94 292.647 45.2978 4.862e-14 ***
hive:method  6  24.31   4.051  0.6271   0.70772    
Residuals   48 310.10   6.460                      
---
Signif. codes:  0 '***' 0.001 '**' 0.01 '*' 0.05 '.' 0.1 ' ' 1
```

Table 16: Sequential ("type-I tests") two-factorial ANOVA of $\log_{10}$(Imidachloprid) on method and hive with interaction between method and hive.

```
> anova( fitlog <- update( fit, log10( .) ~ .))

Analysis of Variance Table

Response: log10(Imi)
            Df Sum Sq Mean Sq F value    Pr(>F)    
hive         2 0.0962 0.04809  2.0617    0.1384    
method       3 3.5326 1.17754 50.4840 7.012e-15 ***
hive:method  6 0.0553 0.00921  0.3950    0.8786    
Residuals   48 1.1196 0.02332                      
---
Signif. codes:  0 '***' 0.001 '**' 0.01 '*' 0.05 '.' 0.1 ' ' 1

> options( oc)  # Resetting the contrasts to original settings; cf. with above!
```

Short formal representation of the models in tab. 15 and 16:

For each hive $h \in \{1, 2, 3\}$ and method $m \in \{1, 2, 3, 4\}$ in bee-group $i \in \{1, \ldots, 5\}$ the observed (untransformed) residue level is denoted $Y_{hm}$ and modelled as:

$$Y_{hm} = \mu_0 + \alpha_h + \beta_m + \gamma_{hm} + e_{hmi} \tag{8}$$

and the log-transformed as

$$\log_{10}(Y_{hm}) = \mu'_0 + \alpha'_h + \beta'_m + \gamma'_{hm} + e'_{hmi} \tag{9}$$

with $\alpha_1$, $\beta_1$, and $\gamma_1$ as well as $\alpha'_1$, $\beta'_1$, and $\gamma'_1$ set to 0 (zero).

Fig. 16 displays three qualitative diagnostic plots for each of the models (8) and (9): in its left column for the model with Imidachloprid on its original scale, and in its right column for the $\log_{10}$-transformed response (see column titles).

**Diagnostic summary:** (Based on fig. 16.) None of the two models indicates serious violations of the model assumption of normality of errors, see the normal q-q plots for the (studentized) residuals (bottom row). However, the plot of residuals vs. fitted values for the untransformed model (top row, left) contains some hints against homoscedasticity which are not seen in the corresponding plot for the log-transformed model (top row, right). In neither model are there any markedly influential observations (outliers) according to Cook's distances (middle row), but for the log-transformed model they seem to look even a little bit more alike.

Comparing the multiple $R^2$-values (goodness-of-fit measure) of 0.75 of the underlying (but not shown) untransformed linear model and 0.77 of the respective log-transformed model (also not shown), the latter appears to fit slightly better. Together with its technical "advantage" to produce positive response values this leads us to prefer the $\log_{10}$-transformed model.

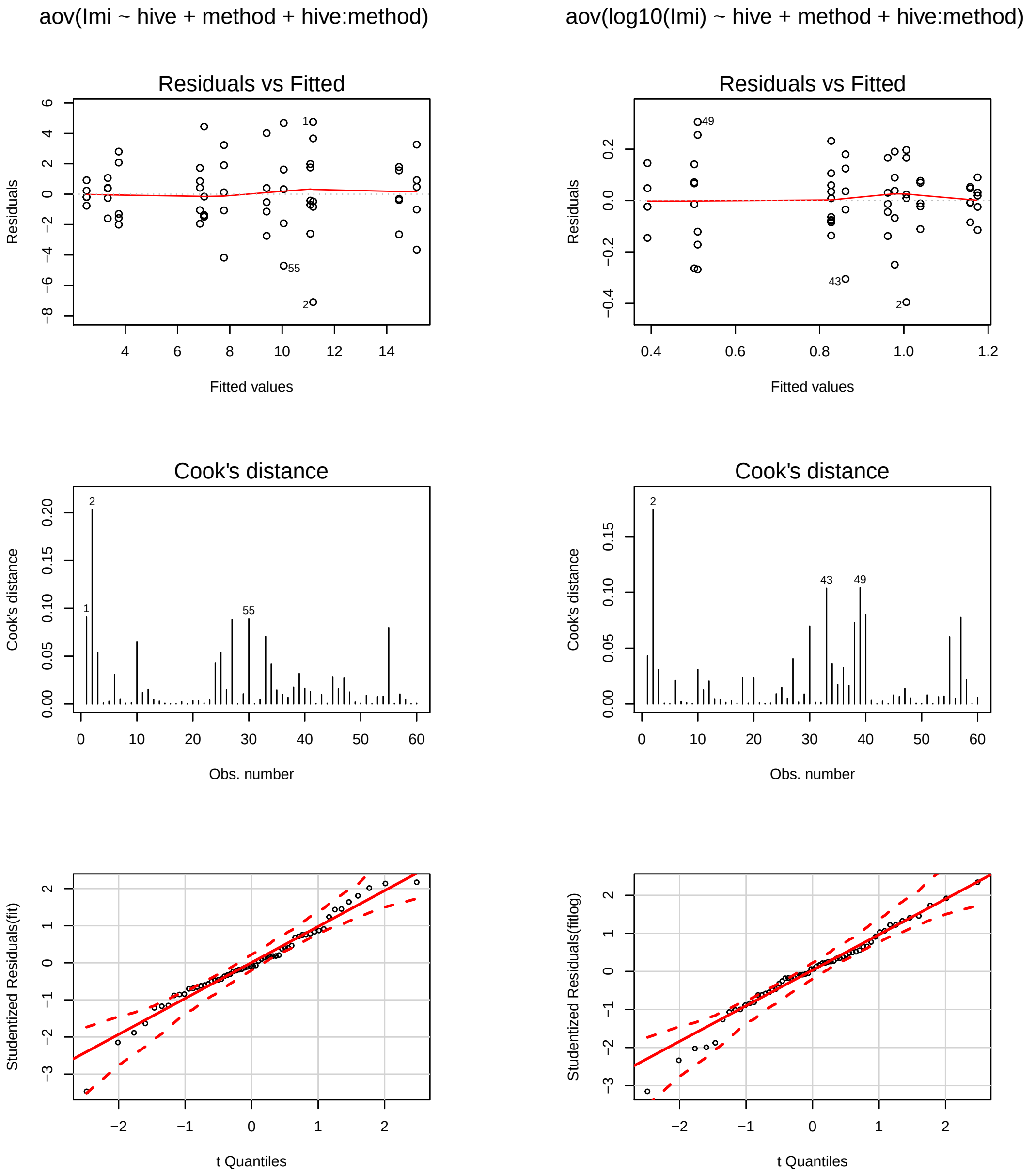


Figure 16: Diagnostic plots for the two-factorial ANOVA models of Imidachloprid on time and hive on the left, and of $\log_{10}$(Imidachloprid) on time and hive on the right. (For a few technical explanations see caption of fig. 4.) (File names: left: *MS-Q2_DiagPlots_Imi.pdf*, right: *MS-Q2_DiagPlots_Imi_B.pdf*)

### Statistical inference for the fitted models

**ANOVA summary:** (Based on tab. 16.) There is no significant difference ($p = 0.138$) between hives in the overall $\log_{10}$(Imidachloprid)-levels (averaged across method). After adjusting for a main effect of hive there is a significant method main effect ($p = 7.01 \times 10^{-15}$). After adjusting for the main effects of hive and method there is no significant interaction effect between hive and method ($p = 0.879$).

### Additional considerations and visualizations: multiple comparisons

Tab. 17 contains the multiple tests for all pairwise comparisons with the reference level, i.e., control, RT1mail (MCC for short), based on the so-called Dunnett contrasts, and tab. 18 presents the pertaining simultaneous confidence intervals. Fig. 17 displays those simultaneous confidence intervals graphically.

*Remark:* The following piece of R-code is for documentation purposes only and shows the technical preparations for the computations underlying tab. 17.

```
> fm <- fitlog

> facnames <- rev( names( fm$contrasts))
> CmpFactor <- WorkData[[ facnames[ 1]]]
> GrpFactor <- WorkData[[ facnames[ 2]]]
> levgrid <- expand.grid( levels( CmpFactor), levels( GrpFactor))
> names( levgrid) <- facnames
> X <- model.matrix( formula( fm)[ -2], data = levgrid,
+                    contrasts.arg = fm$contrasts)
> CM <- contrMat( table( CmpFactor), "Dunnett")
> IM <- diag( nlevels( GrpFactor))
> dimnames( IM) <- list( levels( GrpFactor), levels( GrpFactor))
> Kron <- kronecker( IM, CM, make.dimnames = TRUE)
> ContrastMat <- Kron %*% X
```

Table 17: Imidachloprid (on $\log_{10}$-scale) by method per hive: multiple tests for all pairwise comparisons with the control RT1mail.

```
> set.seed( 20160815)    # To reproduce the simulation-based p-values.
> summary( mcc <- glht( fm, linfct = ContrastMat))

         Simultaneous Tests for General Linear Hypotheses

Fit: aov(formula = log10(Imi) ~ hive + method + hive:method, data = WorkData)

Linear Hypotheses:
                       Estimate Std. Error t value Pr(>|t|)
h1:RT1 - RT1mail == 0   0.04418    0.09659   0.457 0.999740
h1:RT24 - RT1mail == 0 -0.13422    0.09659  -1.390 0.744764
h1:RT48 - RT1mail == 0 -0.57039    0.09659  -5.905  < 1e-04 ***
h2:RT1 - RT1mail == 0   0.19684    0.09659   2.038 0.305074
h2:RT24 - RT1mail == 0 -0.11676    0.09659  -1.209 0.854802
h2:RT48 - RT1mail == 0 -0.46776    0.09659  -4.843 0.000122 ***
h3:RT1 - RT1mail == 0   0.11852    0.09659   1.227 0.844938
h3:RT24 - RT1mail == 0 -0.21153    0.09659  -2.190 0.228895
h3:RT48 - RT1mail == 0 -0.53670    0.09659  -5.556  < 1e-04 ***
---
Signif. codes:  0 '***' 0.001 '**' 0.01 '*' 0.05 '.' 0.1 ' ' 1
(Adjusted p values reported -- single-step method)
```

Table 18: Imidachloprid (on $\log_{10}$-scale) by method per hive: simultaneous confidence intervals for all pairwise comparisons with RT1mail.

```
> set.seed( 20160815)    # To reproduce the simulation-based quantile.
> (simci <- confint( mcc))

         Simultaneous Confidence Intervals

Fit: aov(formula = log10(Imi) ~ hive + method + hive:method, data = WorkData)

Quantile = 2.8571
95% family-wise confidence level


Linear Hypotheses:
                       Estimate lwr      upr
h1:RT1 - RT1mail == 0   0.04418 -0.23179  0.32015
h1:RT24 - RT1mail == 0 -0.13422 -0.41019  0.14175
h1:RT48 - RT1mail == 0 -0.57039 -0.84635 -0.29442
h2:RT1 - RT1mail == 0   0.19684 -0.07913  0.47281
h2:RT24 - RT1mail == 0 -0.11676 -0.39272  0.15921
h2:RT48 - RT1mail == 0 -0.46776 -0.74373 -0.19179
h3:RT1 - RT1mail == 0   0.11852 -0.15745  0.39449
h3:RT24 - RT1mail == 0 -0.21153 -0.48749  0.06444
h3:RT48 - RT1mail == 0 -0.53670 -0.81267 -0.26074
```

**MCC Summary:** Fig. 17 displays the simultaneous confidence intervals for all pairwise comparisons with a control (here: RT1mail) graphically: Each horizontal line segment that does not intersect the dashed vertical line at zero indicates that the respective difference of $\log_{10}$-concentrations (indicated on the vertical axis on the left) is significantly different from zero. This means approximately, that the *ratio* of Imidachloprid's concentrations of the two pertaining treatments are significantly different from 1 (one) on the "original" scale.

Hence, on a family-wise significance level of 95 %, RT1 and RT24 are both not significantly different from RT1mail in any hive, while RT48 is significantly different from RT1mail in all hives (with respect to their average $\log_{10}$(Imidachloprid)-values).

```
> par( mar = c( 4, 8.2, 3, 3))
> plot( simci, xlab = bquote( "Difference of"~log[10]~"-concentrations of"
+                             ~.(names( Response))))
```

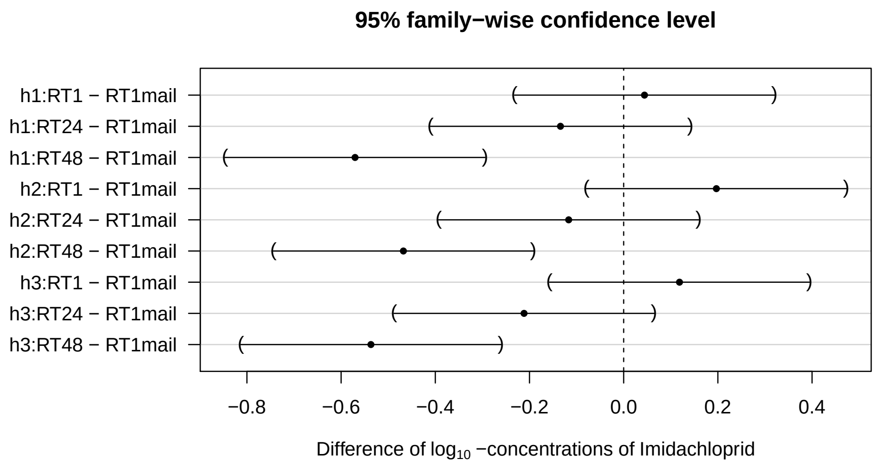


Figure 17: Imidachloprid (on $\log_{10}$-scale!) by method per hive: simultaneous confidence intervals for all pairwise comparisons with RT1mail. (File name: *MS-Q2_AOV_Imi_by_method_and_hive_SimCIplot.pdf*)

**Further considerations and visualizations: MCC for the main effect of method**

Tab. 19 shows simultaneous confidence intervals for the interaction parameters of model tab. 16. According to the practical recommendation in [6, Hsu (1996)], p. 183, we consider the models with and without interaction term as practically equivalent since all simultaneous confidence intervals (and even the not shown unadjusted intervals!) of the interaction effects are not only close to, but in fact contain zero. Hence, we decide to compute multiple tests for all pairwise comparisons with the control and the pertaining simultaneous confidence intervals for the main effect of method ignoring the interaction terms.

*Remark:* The following piece of R-code is shown for documentation purposes only and contains the technical preparations to enable the computations of the simultaneous confidence intervals in tab. 19.

Table 19: Imidachloprid (on $\log_{10}$-scale) by method and hive: simultaneous confidence intervals of the interaction parameters of model (9).

```
> oo <- options( contrasts = c( "contr.treatment", "contr.poly"));  fm.up <- update( fm)
> options( oo);    ni <- prod( sapply( fm$xlevels, length) - 1);    cfm <- tail( coef( fm.up), -ni)
> K <- cbind( matrix( 0, ni, length( cfm)), diag( ni));             rownames( K) <- names( cfm)
> set.seed( 20160816);     confint( glht( fm.up, linfct = K))

         Simultaneous Confidence Intervals

Fit: aov(formula = log10(Imi) ~ hive + method + hive:method, data = WorkData)

Quantile = 2.6821
95% family-wise confidence level


Linear Hypotheses:
                       Estimate lwr      upr
hiveh2:methodRT1 == 0   0.15266 -0.21373  0.51904
hiveh3:methodRT1 == 0   0.07434 -0.29205  0.44072
hiveh2:methodRT24 == 0  0.01746 -0.34892  0.38385
hiveh3:methodRT24 == 0 -0.07730 -0.44369  0.28908
hiveh2:methodRT48 == 0  0.10262 -0.26376  0.46901
hiveh3:methodRT48 == 0  0.03368 -0.33270  0.40006
```

Tab. 20 contains for the two-way model **without interaction** the multiple tests for all pairwise comparisons with the control (here: RT1mail), based on the so-called Dunnett contrasts for the main effect of method, and tab. 21 presents the pertaining simultaneous confidence intervals. Fig. 18 displays those simultaneous confidence intervals graphically.

Table 20: Imidachloprid (on $\log_{10}$-scale) by method and hive: multiple tests for all pairwise comparisons with the control RT1mail.

```
> summary( mcc2 <- glht( fm, linfct = mcp( method = "Dunnett")))

         Simultaneous Tests for General Linear Hypotheses

Multiple Comparisons of Means: Dunnett Contrasts


Fit: aov(formula = log10(Imi) ~ hive + method + hive:method, data = WorkData)

Linear Hypotheses:
                    Estimate Std. Error t value Pr(>|t|)
RT1 - RT1mail == 0   0.11985    0.05577   2.149   0.0930 .
RT24 - RT1mail == 0 -0.15417    0.05577  -2.764   0.0218 *
RT48 - RT1mail == 0 -0.52495    0.05577  -9.413   <0.001 ***
---
Signif. codes:  0 '***' 0.001 '**' 0.01 '*' 0.05 '.' 0.1 ' ' 1
(Adjusted p values reported -- single-step method)
```

Table 21: Imidachloprid (on $\log_{10}$-scale) by method and hive: simultaneous confidence intervals for all pairwise comparisons with RT1mail **ignoring interaction effects**.

```
> (simci <- confint( mcc2))

         Simultaneous Confidence Intervals

Multiple Comparisons of Means: Dunnett Contrasts


Fit: aov(formula = log10(Imi) ~ hive + method + hive:method, data = WorkData)

Quantile = 2.4244
95% family-wise confidence level


Linear Hypotheses:
                     Estimate lwr      upr
RT1 - RT1mail == 0    0.11985 -0.01535  0.25505
RT24 - RT1mail == 0  -0.15417 -0.28937 -0.01897
RT48 - RT1mail == 0  -0.52495 -0.66015 -0.38975


> par( mar = c( 4, 8.2, 3, 3))
> plot( simci, xlab = bquote( "Difference of"~log[10]~"-concentrations of"
+                              ~.(names( Response))))
```

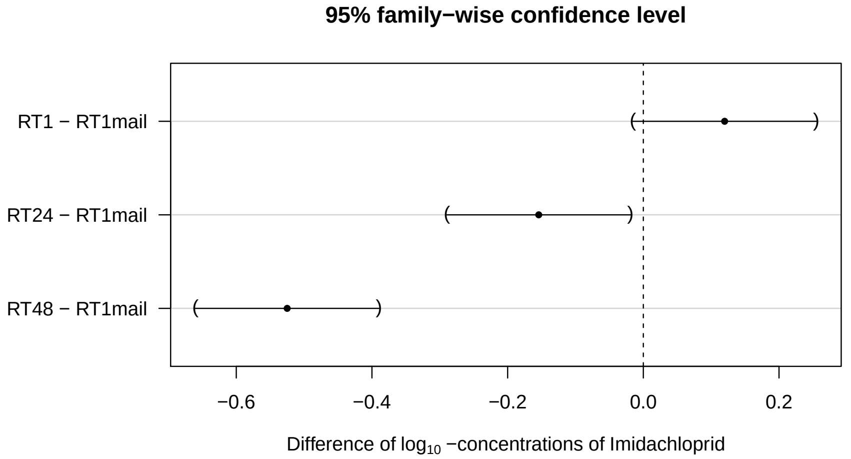


Figure 18: Imidachloprid (on $\log_{10}$-scale!) by method and hive: simultaneous confidence intervals for all pairwise comparisons with RT1mail **ignoring interaction effects**. (File name: *MS-Q2_AOV_Imi_by_method_and_hive_SimCIplot2.pdf*)

**MCC Summary 2:** Fig. 18 displays the simultaneous confidence intervals for all pairwise comparisons with a control, i.e, with the reference level (here: RT1mail), graphically: Each horizontal line segment that does not intersect the dashed vertical line at zero indicates that the respective difference of $\log_{10}$-concentrations (indicated on the vertical axis on the left) is significantly different from zero. This means approximately, that the *ratio* of Imidachloprid's concentrations of the two pertaining treatments are significantly different from 1 (one) on the "original" scale.

Hence, on a family-wise significance level of 95 %, RT1 is not significantly different from RT1mail, while RT24 and RT48 are both significantly different from RT1mail (with respect to their average $\log_{10}$(Imidachloprid)-values).

## 2.3 Question 3: UV-light

Does heavy exposure to UV-light accelerate the rate of degradation?

Preparations:

```
> lev.of.int <- c( "RT1", "RT24", "RT48", "RT24UV")   # treatment levels of interest
> reflev <- "RT24UV"   # reference level for, e.g., multiple comparisons
```

1. Extracting the rows of `Bees`, whose corresponding elements in `method` are in the set {`"RT1"`, `"RT24"`, `"RT48"`, `"RT24UV"`}, i.e., which belong to the treatments RT1, RT24, RT48 or RT24UV, and storing the "extract" in a new data frame named `BOUV`:

   ```
   > BOUV <- droplevels( subset( Bees, subset = method %in% lev.of.int))
   ```

2. Changing `method`'s level order, so that `RT24UV` is the first level and hence the reference level in later analyses (like multiple comparisons):

   ```
   > BOUV$method <- relevel( BOUV$method, ref = reflev)
   ```

3. Generating variables containing some "values" which will be needed and repeatedly (!) used in the following paragraphs (with the intention to design flexible and efficient code that can easily be modified and reused ("recycled") in subsequent paragraphs):

   ```
   > Response <- c( Imidachloprid = "Imi");   Treatment <- "method";   Group <- "hive"
   > WorkData <- BOUV
   ```

### 2.3.1 Imidachloprid

Question, stated a bit more precisely: Is the process of degradation of Imidachloprid speeding up by heavy UV exposure in comparison to the "normal" process of degradation? In this scenario an extreme sun exposure is simulated by means of an UV lamp.

Even more precisely: Is the average concentration of Imidachloprid in treatment RT24UV lower than in the other three and especially RT24?

To this end: Two-factorial ANOVA of Imidachloprid on the treatment (in the following called method) and on hive (with interaction) with subsequent "multiple comparisons with a control" and calculation of simultaneous confidence intervals for the respective differences.

Points to consider: Repeated measurements on the same hive may induce dependencies within each group of measurements from the same hive, so analoguos remarks apply as in the "points to consider" on page 8.

**Graphical EDA**

Fig. 19 shows strip plots of Imidachloprid values on the original scale (left) and on the decimal logarithmic scale (right), grouped by method and color-coded for all hives overlaid. For a few more details about this sort of presentation see the explanations for figures 1 and 2, and recall the remark on logarithmic transformations on 8.

Fig. 20 shows in principle the same, but uses for each hive a separate panel and is amended with indications of the mean values for all method-groups which are joined by straight lines (just) for the purpose of visualization.

The latter can also be interpreted as a so-called interaction plot to assess if an interaction may be present between hive and method. Indication for interaction would be given if the hive-specific "profiles" of line segments "along" method were *not* parallel. This is not strong enough the case here, esp. in view of the variability of the raw data around their means, and will be confirmed by a formal test yielding a non-significant interaction effect.

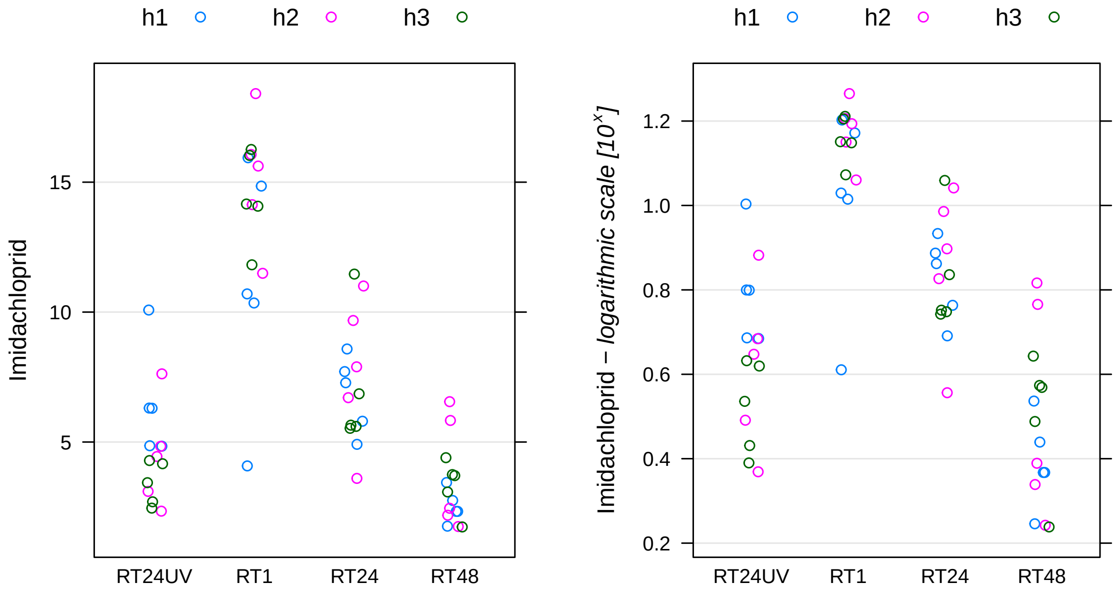


Figure 19: Imidachloprid (left on original scale, right on $\log_{10}$-scale) by method with hive-specific plotting colors (horizontally jittered; for explanations see caption of fig. 1 and for details see text there). (File names: left: *MS-Q3_EDA_Imi_by_hive_1_NOLOG.pdf*, right: *MS-Q3_EDA_Imi_by_hive_1_LOG.pdf*)

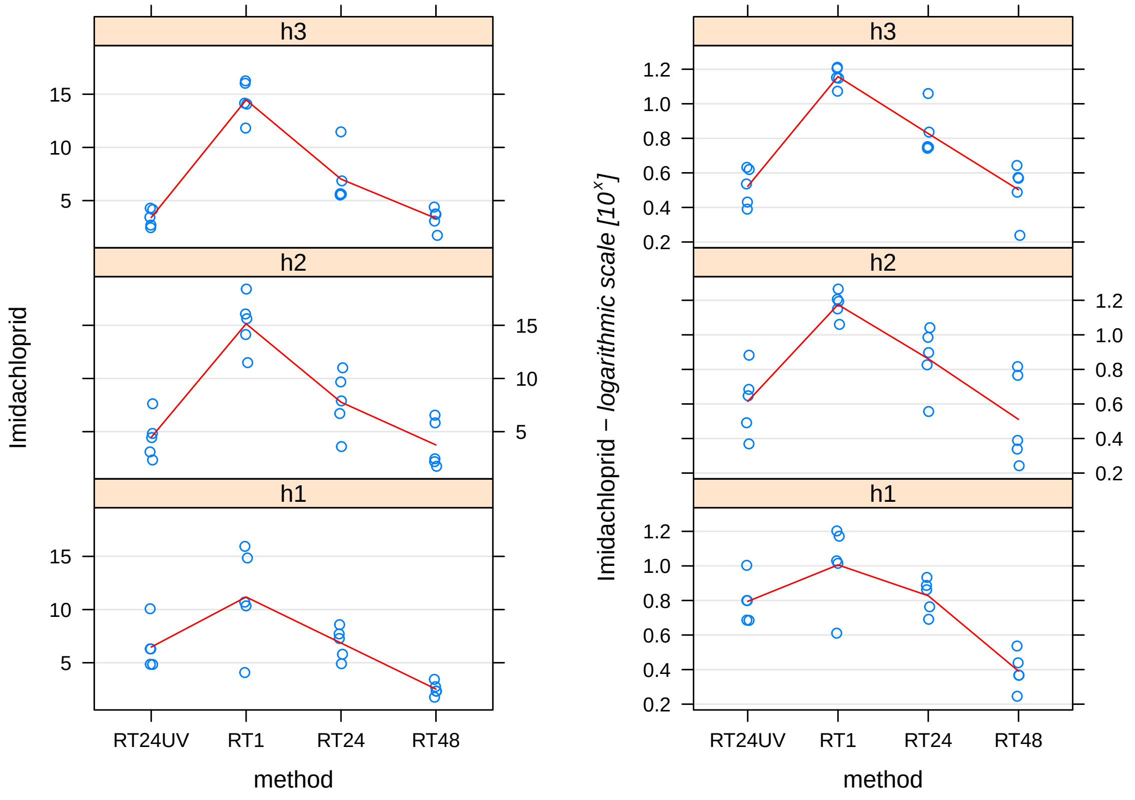


Figure 20: Imidachloprid (left on original scale, right on $\log_{10}$-scale) by method per hive with method-specific averages joined by line segments (interaction profiles (red)). (File names: left: *MS-Q3_EDA_Imi_by_hive_2_NOLOG.pdf* right: *MS-Q3_EDA_Imi_by_hive_2_LOG.pdf*)

**Model fitting & model diagnostics**

Tab. 22 presents the two-factorial analysis of variance (ANOVA) table of Imidachloprid on method and hive with interaction using sequential tests (aka "type-I tests") for the model terms. For details on structure and interpretation of the ANOVA table see the explanations regarding tab. 3.

Table 22: Sequential ("type-I tests") two-factorial ANOVA of Imidachloprid on method and hive with interaction between method and hive. (`update()` is used here for the same reason as in tab. 1.)

```
>    # 'Building' the model formula using variables (for the sake of flexibility):
> form <- formula( paste( Response, "~", Group, "*", Treatment))
> fit <- aov( form, data = WorkData)   # Fitting the underlying linear model.
> fit <- update( fit, ~ .)
> anova( fit)

Analysis of Variance Table

Response: Imi
            Df Sum Sq Mean Sq F value    Pr(>F)
hive         2  11.13   5.567  1.0385   0.36180
method       3 941.54 313.847 58.5491 4.565e-16 ***
hive:method  6  64.34  10.723  2.0005   0.08399 .
Residuals   48 257.30   5.360
---
Signif. codes:  0 '***' 0.001 '**' 0.01 '*' 0.05 '.' 0.1 ' ' 1
```

Table 23: Sequential ("type-I tests") two-factorial ANOVA of $\log_{10}$(Imidachloprid) on method and hive with interaction between method and hive.

```
> anova( fitlog <- update( fit, log10( .) ~ .))

Analysis of Variance Table

Response: log10(Imi)
            Df Sum Sq Mean Sq F value    Pr(>F)
hive         2 0.0182 0.00910  0.3634   0.69720
method       3 3.4398 1.14660 45.8066 3.993e-14 ***
hive:method  6 0.3091 0.05152  2.0583   0.07588 .
Residuals   48 1.2015 0.02503
---
Signif. codes:  0 '***' 0.001 '**' 0.01 '*' 0.05 '.' 0.1 ' ' 1
```

Short formal representation of the models in tab. 22 and 23 is completely analogue to the one in (8) and (9), respectively.

Fig. 21 displays three qualitative diagnostic plots for each of the models (8) and (9): in its left column for the model with Imidachloprid on its original scale, and in its right column for the $\log_{10}$-transformed response (see column titles).

**Diagnostic summary:** (Based on fig. 21.) None of the two models indicates serious violations of the model assumption of normality of errors, as can be seen in the normal q-q plots for the (studentized) residuals (bottom row). However, the plot of residuals vs. fitted values for the untransformed model (top row, left) contains hints against homoscedasticity which appear to be (only a little) weaker in the corresponding plot for the log-transformed model (top row, right). In neither model are there any markedly influential observations

(outliers) according to Cook's distances (middle row), but for the log-transformed model they appear a bit more alike.

Comparing the multiple $R^2$-values (goodness-of-fit measure) of 0.8 of the underlying (but not shown) untransformed linear model and 0.76 of the respective log-transformed model (also not shown), the first appears to fit better. In view of this and the diagnostic summary, we prefer the untransformed model (despite its technical "disadvantage" of possibly producing negative response values).

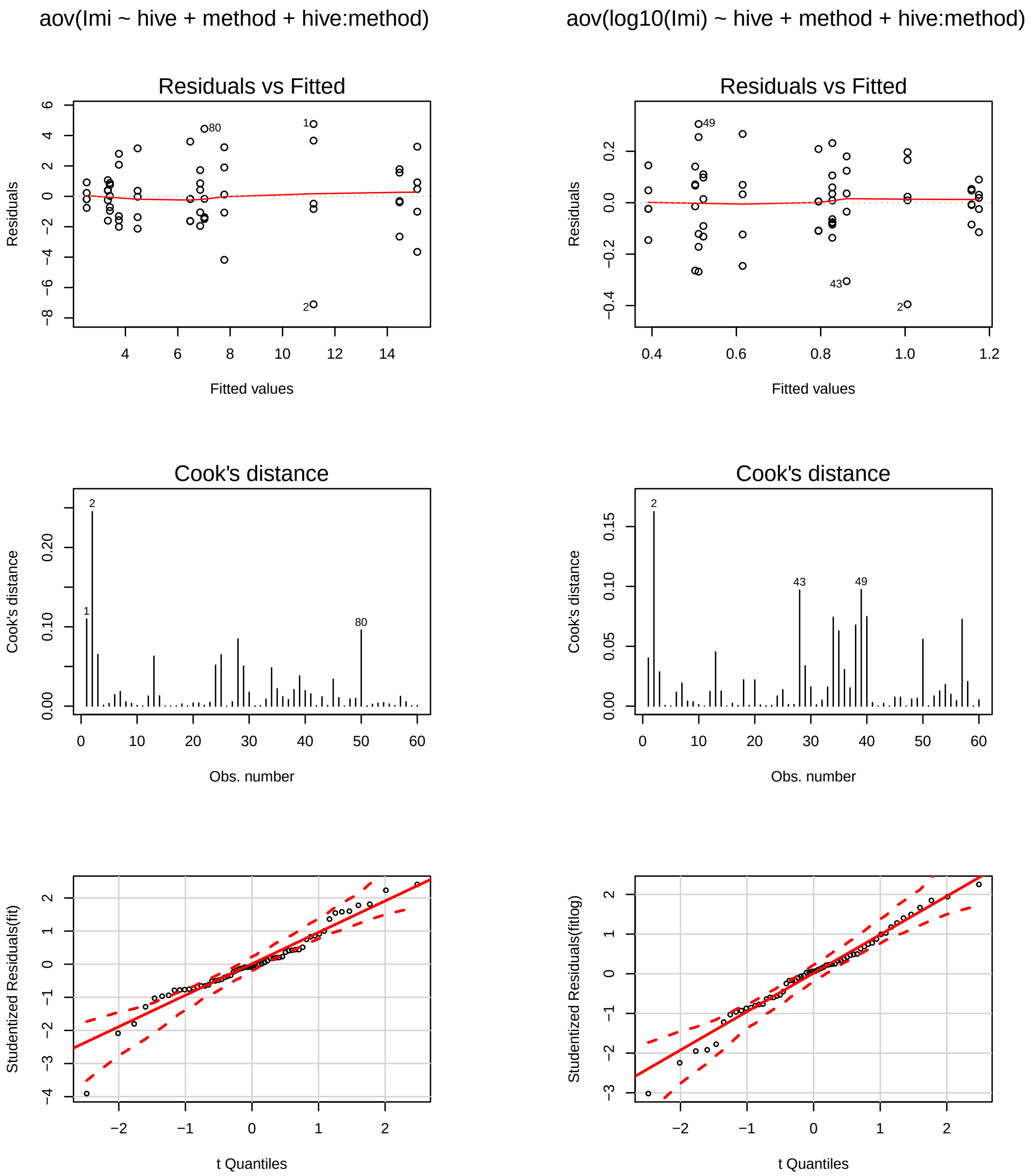


Figure 21: Diagnostic plots for the two-factorial ANOVA models of Imidachloprid on time and hive on the left, and of $\log_{10}$(Imidachloprid) on time and hive on the right. (For a few technical explanations see caption of fig. 4.) (File names: left: *MS-Q3_DiagPlots_Imi.pdf*, right: *MS-Q3_DiagPlots_Imi_B.pdf*)

### Statistical inference for the fitted models

**ANOVA summary:** (Based on tab. 22.) There is no significant difference ($p = 0.362$) between hives in the overall Imidacloprid-levels (averaged across method). After adjusting for a main effect of hive there is a significant method main effect ($p = 4.56 \times 10^{-16}$). After adjusting for the main effects of hive and method there is no significant interaction effect between hive and method ($p = 8.4 \times 10^{-2}$).

**Additional considerations and visualizations: multiple comparisons**

Tab. 24 contains the multiple tests for all pairwise comparisons with the control, i.e., reference level RT24UV (MCC for short), based on the so-called Dunnett contrasts, and tab. 25 presents the pertaining simultaneous confidence intervals. Fig. 22 displays those simultaneous confidence intervals graphically.

*Remark:* The following piece of R-code is for documentation purposes only and shows the technical preparations for the computations underlying tab. 24.

```
> fm <- fit
> facnames <- rev( names( fm$contrasts))
> CmpFactor <- WorkData[[ facnames[ 1]]]
> GrpFactor <- WorkData[[ facnames[ 2]]]
> levgrid <- expand.grid( levels( CmpFactor), levels( GrpFactor))
> names( levgrid) <- facnames
> X <- model.matrix( formula( fm)[ -2], data = levgrid,
+                    contrasts.arg = fm$contrasts)
> CM <- contrMat( table( CmpFactor), "Dunnett")
> IM <- diag( nlevels( GrpFactor))
> dimnames( IM) <- list( levels( GrpFactor), levels( GrpFactor))
> Kron <- kronecker( IM, CM, make.dimnames = TRUE)
> ContrastMat <- Kron %*% X
```

Table 24: Imidachloprid by method per hive: multiple tests for all pairwise comparisons with the reference level RT24UV.

```
> set.seed( 20160815)    # To reproduce the simulation-based p-values.
> summary( mcc <- glht( fm, linfct = ContrastMat))

         Simultaneous Tests for General Linear Hypotheses

Fit: aov(formula = Imi ~ hive + method + hive:method, data = WorkData)

Linear Hypotheses:
                      Estimate Std. Error t value Pr(>|t|)
h1:RT1 - RT24UV == 0    4.7082     1.4643   3.215   0.0193 *
h1:RT24 - RT24UV == 0   0.3802     1.4643   0.260   1.0000
h1:RT48 - RT24UV == 0  -3.9536     1.4643  -2.700   0.0739 .
h2:RT1 - RT24UV == 0   10.6770     1.4643   7.292   <1e-04 ***
h2:RT24 - RT24UV == 0   3.3089     1.4643   2.260   0.1990
h2:RT48 - RT24UV == 0  -0.7151     1.4643  -0.488   0.9996
h3:RT1 - RT24UV == 0   11.0602     1.4643   7.553   <1e-04 ***
h3:RT24 - RT24UV == 0   3.6104     1.4643   2.466   0.1279
h3:RT48 - RT24UV == 0  -0.0768     1.4643  -0.052   1.0000
---
Signif. codes:  0 '***' 0.001 '**' 0.01 '*' 0.05 '.' 0.1 ' ' 1
(Adjusted p values reported -- single-step method)
```

Table 25: Imidachloprid by method per hive: simultaneous confidence intervals for all pairwise comparisons with RT24UV.

```
> set.seed( 20160815)    # To reproduce the simulation-based quantile.
> (simci <- confint( mcc))

         Simultaneous Confidence Intervals

Fit: aov(formula = Imi ~ hive + method + hive:method, data = WorkData)

Quantile = 2.857
95% family-wise confidence level


Linear Hypotheses:
                      Estimate lwr     upr
h1:RT1 - RT24UV == 0   4.7082   0.5247  8.8918
h1:RT24 - RT24UV == 0  0.3802  -3.8033  4.5638
h1:RT48 - RT24UV == 0 -3.9536  -8.1371  0.2300
h2:RT1 - RT24UV == 0  10.6770   6.4935 14.8606
h2:RT24 - RT24UV == 0  3.3088  -0.8747  7.4924
h2:RT48 - RT24UV == 0 -0.7152  -4.8987  3.4684
h3:RT1 - RT24UV == 0  11.0602   6.8767 15.2437
h3:RT24 - RT24UV == 0  3.6104  -0.5731  7.7940
h3:RT48 - RT24UV == 0 -0.0768  -4.2603  4.1067
```

**MCC summary:** Fig. 22 displays the simultaneous confidence intervals for all pairwise comparisons with a control (here: the reference level RT24UV) graphically: Each horizontal line segment that does not intersect the dashed vertical line at zero indicates that the respective difference (indicated on the vertical axis on the left) is significantly different from zero.

Hence, on a family-wise significance level of 95 %, RT1 and RT24UV are significantly different from each other in each hive, while there is no significant difference between RT24UV and either of RT24 and RT48 in all hives (with respect to their average Imidachloprid-values).

```
> par( mar = c( 4, 8.2, 3, 3))
> plot( simci, xlab = paste0( "Difference of ", names( Response),
+                             "-concentrations"))
```

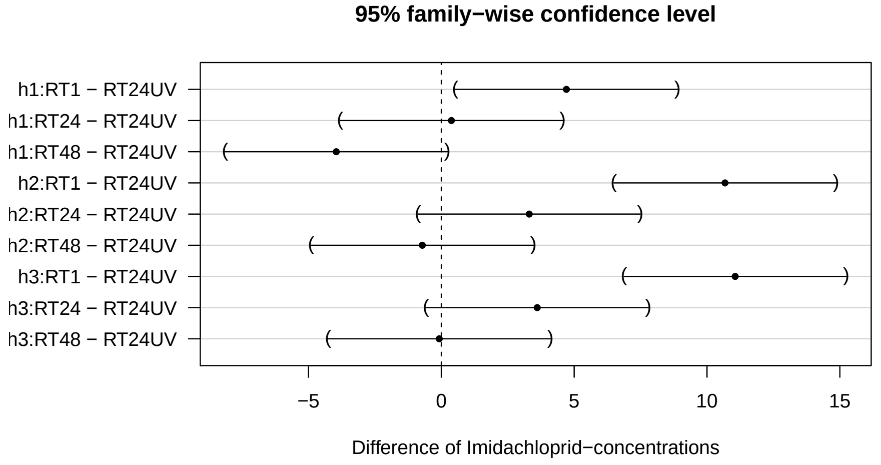


Figure 22: Imidachloprid by method per hive: simultaneous confidence intervals for all pairwise comparisons with RT24UV. (File name: *MS-Q3_AOV_Imi_by_method_and_hive_SimCIplot.pdf*)

**Further considerations and visualizations: MCC for the main effect of method**

Tab. 26 shows simultaneous confidence intervals for the interaction parameters of model tab. 22. According to the practical recommendation in [6, Hsu (1996)], p. 183, we consider the models with and without interaction term as practically equivalent since all *but two* of the simultaneous confidence intervals of the interaction effects are not only close to, but in fact contain zero. The two that do not cover zero are anyhow very close to zero (and quite long). Hence, we decide to compute multiple tests for all pairwise comparisons with the control, i.e., the reference level, and the pertaining simultaneous confidence intervals for the main effect of method ignoring the interaction terms.

Table 26: Imidachloprid by method and hive: simultaneous confidence intervals of the interaction parameters of model (8).

```
> oo <- options( contrasts = c( "contr.treatment", "contr.poly"));  fm.up <- update( fm)
> options( oo);    ni <- prod( sapply( fm$xlevels, length) - 1);    cfm <- tail( coef( fm.up), -ni)
> K <- cbind( matrix( 0, ni, length( cfm)), diag( ni));              rownames( K) <- names( cfm)
> set.seed( 20160816);      confint( glht( fm.up, linfct = K))

         Simultaneous Confidence Intervals

Fit: aov(formula = Imi ~ hive + method + hive:method, data = WorkData)

Quantile = 2.6798
95% family-wise confidence level


Linear Hypotheses:
                       Estimate lwr     upr
hiveh2:methodRT1 == 0   5.9688   0.4193 11.5183
hiveh3:methodRT1 == 0   6.3520   0.8025 11.9015
hiveh2:methodRT24 == 0  2.9286  -2.6209  8.4781
hiveh3:methodRT24 == 0  3.2302  -2.3193  8.7797
hiveh2:methodRT48 == 0  3.2384  -2.3111  8.7879
hiveh3:methodRT48 == 0  3.8768  -1.6727  9.4263
```

Tab. 27 contains for the two-way model **without interaction** the multiple tests for all pairwise comparisons with the control (here: the reference level RT24UV), based on the so-called Dunnett contrasts for the main effect of method, and tab. 28 presents the pertaining simultaneous confidence intervals. Fig. 23 displays those simultaneous confidence intervals graphically.

Table 27: Imidachloprid by method and hive: multiple tests for all pairwise comparisons with the control RT24UV.

```
> summary( mcc2 <- glht( fm, linfct = mcp( method = "Dunnett")))

         Simultaneous Tests for General Linear Hypotheses

Multiple Comparisons of Means: Dunnett Contrasts


Fit: aov(formula = Imi ~ hive + method + hive:method, data = WorkData)

Linear Hypotheses:
                    Estimate Std. Error t value Pr(>|t|)
RT1 - RT24UV == 0    8.8152     0.8454  10.427   <0.001 ***
RT24 - RT24UV == 0   2.4332     0.8454   2.878   0.0163 *
RT48 - RT24UV == 0  -1.5818     0.8454  -1.871   0.1637
---
Signif. codes:  0 '***' 0.001 '**' 0.01 '*' 0.05 '.' 0.1 ' ' 1
(Adjusted p values reported -- single-step method)
```

Table 28: Imidachloprid by method and hive: simultaneous confidence intervals for all pairwise comparisons with RT24UV **ignoring interaction effects**.

```
> (simci <- confint( mcc2))

         Simultaneous Confidence Intervals

Multiple Comparisons of Means: Dunnett Contrasts


Fit: aov(formula = Imi ~ hive + method + hive:method, data = WorkData)

Quantile = 2.4244
95% family-wise confidence level


Linear Hypotheses:
                   Estimate lwr     upr
RT1 - RT24UV == 0   8.8152   6.7656 10.8647
RT24 - RT24UV == 0  2.4332   0.3836  4.4828
RT48 - RT24UV == 0 -1.5818  -3.6314  0.4677


> par( mar = c( 4, 8.2, 3, 3))
> plot( simci, xlab = paste0( "Difference of ", names( Response),
+                               "-concentrations"))
```

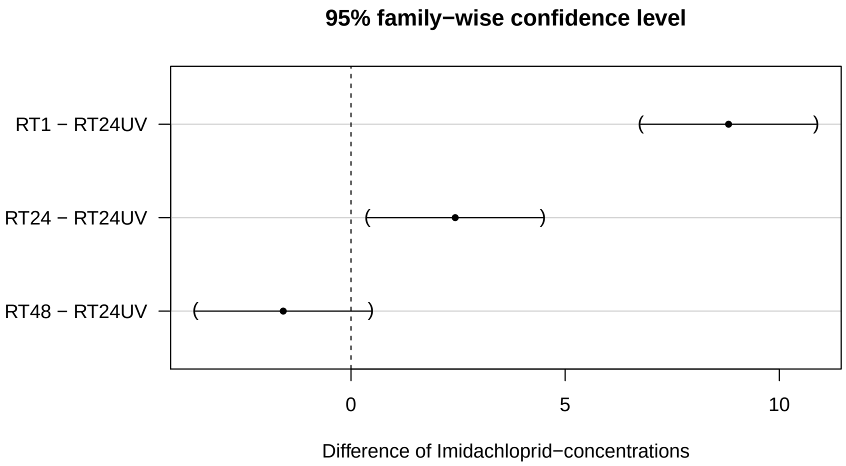


Figure 23: Imidachloprid by method and hive: simultaneous confidence intervals for all pairwise comparisons with RT24UV **ignoring interaction effects**. (File name: *MS-Q3_AOV_Imi_by_method_and_hive_SimCIplot2.pdf*)

**MCC Summary 2:** Fig. 23 displays the simultaneous confidence intervals for all pairwise comparisons with a control (here: the reference level RT24UV) graphically: Each horizontal line segment that does not intersect the dashed vertical line at zero indicates that the respective difference of concentrations (indicated on the vertical axis on the left) is significantly different from zero.

Hence, on a family-wise significance level of 95 %, RT1 and RT24 are both significantly different from RT24UV, while RT48 is not significantly different from RT24UV (with respect to their average Imidachloprid-values).

## 2.4 Question 4: Single vs. group feeding

Does the feeding method (single vs. group feeding) influence the measurement results and/or the quality *(variance!)* of the data?

Preparations:

```
> lev.of.int <- c( "RT1", "RT24", "RT24GF", "RT48")   # treatment levels of interest
> reflev <- "RT24GF"   # reference level for, e.g., multiple comparisons
```

1. Extracting the rows of `Bees`, whose corresponding elements in `method` are in the set {`"RT1"`, `"RT24"`, `"RT24GF"`, `"RT48"`}, i.e., which belong to the treatments RT1, RT24, RT24GF or RT48, and storing the "extract" in a new data frame named `BOGFX`:

```
> BOGFX <- droplevels( subset( Bees, subset = method %in% lev.of.int))
```

2. Setting `method`'s level order explicitly as in `lev.of.int` for the graphical displays only (but `RT24GF` will be the reference level in later analyses (like multiple comparisons)):

```
> BOGFX$method <- factor( BOGFX$method, levels = lev.of.int)
```

3. Generating variables containing some "values" which will be needed and repeatedly (!) used in the following paragraphs (with the intention to design flexible and efficient code that can easily be modified and reused ("recycled") in subsequent paragraphs):

```
> Response <- c( Imidachloprid = "Imi");   Treatment <- "method";   Group <- "hive"
> WorkData <- BOGFX
```

### 2.4.1 Imidachloprid

Question, stated a bit more precisely and with additional information: There are actually two questions here: Does the feeding method (single vs. group feeding) influence the measurement results of Imidachloprid with respect to their ...

a) quality quantified by their variance (regarding only RT24 and RT24GF)?

b) location on the measurement scale (regarding RT1, RT24, RT24GF and RT48)?

Note 1: In the main experiment bees were fed individually to minimise "dilution"-effects by trophalaxis (feeding gustation drops from one bee to another). In the group feeding experiment 10 bees had access to the 10-fold volume of the same food source as in the other experiments. Every other factor was kept similar.

Note 2: Variance is analysed first because the result typically influences the analysis of location.

The key questions even more precisely:

a) Is the variance of Imidachloprid-concentrations in treatment RT24GF different from that in RT24 (taking hives into consideration!)?

b) Is the average concentration of Imidachloprid in treatment RT24GF different from that in RT1, RT24 or RT48 (taking hives into consideration!)?

To these ends: Variance comparison of Imidachloprid between the *combinations* of two treatments, namely RT24GF and RT24, and hives (i.e., between 6 samples), and two-factorial ANOVA of Imidachloprid on (all four) treatments and hive (with interaction) with subsequent "multiple comparisons with a control" and calculation of simultaneous confidence intervals for the respective differences. (In the following, treatment shall be called method.)

Points to consider: Repeated measurements on the same hive may induce dependencies within each group of measurements from the same hive, so analoguos remarks apply as in the "points to consider" on page 8.

#### Graphical EDA

Fig. 24 shows strip plots of Imidachloprid values on the original scale (left) and on the decimal logarithmic scale (right), grouped by method and color-coded for all hives overlaid. For a few more details about this sort of presentation see the explanations for figures 1 and 2, and recall the remark on logarithmic transformations on 8. Fig. 25 shows in principle the same, but uses for each hive a separate panel and is amended with indications of the mean values for all method-groups which are joined by straight lines just for the purpose of visualization.

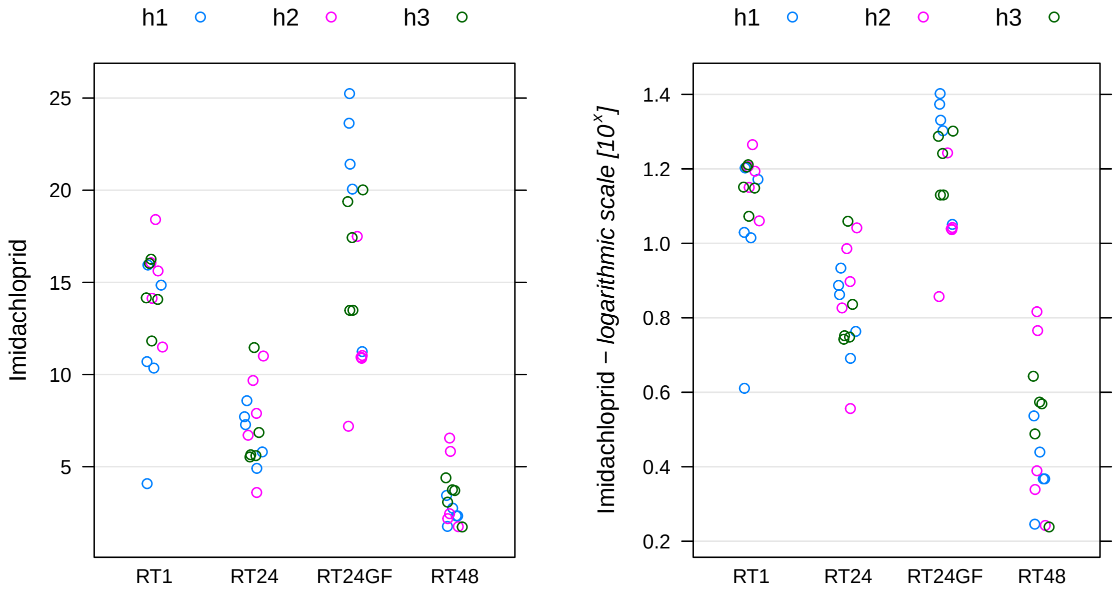


Figure 24: Imidachloprid (left on original scale, right on $\log_{10}$-scale) by method with hive-specific plotting colors (horizontally jittered; for explanations see caption of fig. 1 and for details see text there). (File names: left: *MS-Q4_EDA_Imi_by_hive_1_NOLOG.pdf*, right: *MS-Q4_EDA_Imi_by_hive_1_LOG.pdf*)

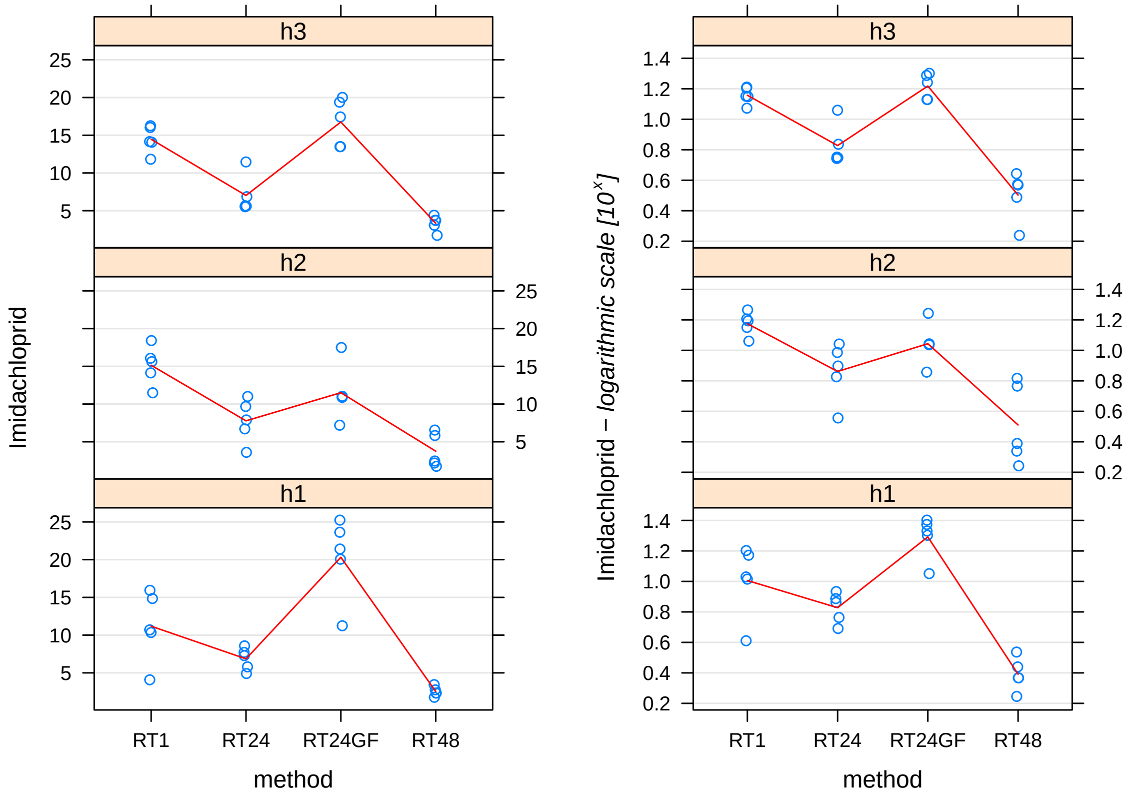


Figure 25: Imidachloprid (left on original scale, right on $\log_{10}$-scale) by method per hive with method-specific averages joined by line segments (interaction profiles (red)). (File names: left: *MS-Q4_EDA_Imi_by_hive_2_NOLOG.pdf* right: *MS-Q4_EDA_Imi_by_hive_2_LOG.pdf*)

#### 2.4.1.1 Comparing variances

First of all, tables 29 and 30 present just for descriptive purposes the sample variances and standard deviations of Imidachloprid and $\log_{10}$(Imidachloprid), respectively, in groups of method-hive-combinations or of methods alone.

Table 29: Sample variances and standard deviations of Imidachloprid in various groups.

```
> vartab <- with( WorkData, cbind( tapply( Imi, list( hive, method), var),
+                                  tapply( Imi, list( hive, method), sd)))
> cgrp <- c( "Variance", "Standard deviation");     dec <- 3
> my.latex( vartab, cgroup = cgrp, dec = dec, table.env = FALSE)
```

| | **Variance** | | | | **Standard deviation** | | | |
|---|---|---|---|---|---|---|---|---|
| | RT1 | RT24 | RT24GF | RT48 | RT1 | RT24 | RT24GF | RT48 |
| h1 | 21.864 | 2.196 | 29.708 | 0.387 | 4.676 | 1.482 | 5.451 | 0.622 |
| h2 | 6.522 | 8.158 | 13.844 | 5.084 | 2.554 | 2.856 | 3.721 | 2.255 |
| h3 | 3.226 | 6.472 | 9.841 | 1.018 | 1.796 | 2.544 | 3.137 | 1.009 |

```
> vartab <- with( WorkData, t( c( tapply( Imi, method, var),
+                                 tapply( Imi, method, sd))))
> my.latex( vartab, cgroup = cgrp, dec = dec, table.env = FALSE)
```

| **Variance** | | | | **Standard deviation** | | | |
|---|---|---|---|---|---|---|---|
| RT1 | RT24 | RT24GF | RT48 | RT1 | RT24 | RT24GF | RT48 |
| 12.237 | 4.979 | 29.289 | 2.133 | 3.498 | 2.231 | 5.412 | 1.460 |

Table 30: Sample variances and standard deviations of $\log_{10}$(Imidachloprid) in various groups.

```
> vartab <- with( WorkData, cbind( tapply( log10( Imi), list( hive, method), var),
+                                  tapply( log10( Imi), list( hive, method), sd)))
> my.latex( vartab, cgroup = cgrp, dec = dec, table.env = FALSE)
```

| | **Variance** | | | | **Standard deviation** | | | |
|---|---|---|---|---|---|---|---|---|
| | RT1 | RT24 | RT24GF | RT48 | RT1 | RT24 | RT24GF | RT48 |
| h1 | 0.056 | 0.010 | 0.020 | 0.011 | 0.236 | 0.098 | 0.140 | 0.107 |
| h2 | 0.006 | 0.036 | 0.019 | 0.069 | 0.076 | 0.189 | 0.137 | 0.262 |
| h3 | 0.003 | 0.018 | 0.007 | 0.025 | 0.056 | 0.135 | 0.083 | 0.158 |

```
> vartab <- with( WorkData, t( c( tapply( log10( Imi), method, var),
+                                 tapply( log10( Imi), method, sd))))
> my.latex( vartab, cgroup = cgrp, dec = dec, table.env = FALSE)
```

| **Variance** | | | | **Standard deviation** | | | |
|---|---|---|---|---|---|---|---|
| RT1 | RT24 | RT24GF | RT48 | RT1 | RT24 | RT24GF | RT48 |
| 0.025 | 0.019 | 0.025 | 0.033 | 0.157 | 0.136 | 0.157 | 0.182 |

Tab. 31 presents firstly Bartlett's several-sample test for homogeneity/equality of variances (= "homoscedasticity") of Imidachloprid, here between groups of method-hive-combinations for the methods RT24GF and RT24, and secondly, the nonparametric Fligner-Killeen test of the same hypothesis. Fig. 25 (left) *contains* the corresponding exploratory display for this analysis (and already "visually announced" the inferential result). Tab. 32 and Fig. 25 (right) do the respective for the $\log_{10}$-transformed Imidachloprid-values.

Table 31: Bartlett's test for homogeneity of variances of Imidachloprid between method-hive-combinations for the methods RT24GF and RT24.

```
> form <- formula( paste( Response, "~ I(", Treatment, ":", Group, ")"))
> grps <- WorkData[[ Treatment]] %in% c( reflev, "RT24")   # Only 2 methods,
> bartlett.test( form, data = WorkData, subset = grps)     # but all hives!

        Bartlett test of homogeneity of variances

data:  Imi by I(method:hive)
Bartlett's K-squared = 6.1986, df = 5, p-value = 0.2874

> fligner.test( form, data = WorkData, subset = grps) # Nonparametric scale test.

        Fligner-Killeen test of homogeneity of variances

data:  Imi by I(method:hive)
Fligner-Killeen:med chi-squared = 2.3748, df = 5, p-value = 0.7952
```

Table 32: Bartlett's test for homogeneity of variances of $\log_{10}$(Imidachloprid) between method-hive-combinations for the methods RT24GF and RT24.

```
> form <- update( form, log10( . ) ~ .)                  # Also only 2 methods,
> bartlett.test( form, data = WorkData, subset = grps)   # but all hives.

        Bartlett test of homogeneity of variances

data:  log10(Imi) by I(method:hive)
Bartlett's K-squared = 2.9273, df = 5, p-value = 0.7112

> fligner.test( form, data = WorkData, subset = grps) # Nonparametric scale test.

        Fligner-Killeen test of homogeneity of variances

data:  log10(Imi) by I(method:hive)
Fligner-Killeen:med chi-squared = 1.1882, df = 5, p-value = 0.946
```

Tables 33 and 34 show the results for all pairwise comparisons with a control, here the reference level RT24GF, for two-sample $F$-tests for homoscedasticity of Imidachloprid and $\log_{10}$(Imidachloprid), respectively, between methods. (Recall that these two-sample $F$-tests are based on the ratios of the variances which are presented in tab. 29, and that the $F$-test in fact tests the hypothesis that the ratio of variances equals 1.)

Table 33: All pairwise comparisons with RT24GF using the $F$-test for equality of two variances of Imidachloprid between methods, using Holm's method for adjusting $p$-values.

```
> form <- formula( paste( Response, "~", Treatment))
> pvals <- sapply( setdiff( lev.of.int, reflev),
+   function( lev) {
+     grps <- WorkData[[ Treatment]] %in% c( lev, reflev)
+     wd <- droplevels( WorkData[ grps, ])
+     wd[[ Treatment]] <- relevel( wd[[ Treatment]], ref = reflev)
+     var.test( form, data = wd)[ c( "estimate", "p.value")]
+     } )
> pmat <- unlist( pvals);   dim( pmat) <- dim( pvals)
> dimnames( pmat) <- list( c( "ratio of variances", "raw p-value"),
+                          paste0( reflev, "/", colnames( pvals)))
> pmat <- rbind( pmat, "Holm-adjusted p-value" = p.adjust( pmat[ "raw p-value",]))
> my.latex( pmat, rdec = c( 3, 6, 6), table.env = FALSE)
```

| | RT24GF/RT1 | RT24GF/RT24 | RT24GF/RT48 |
|---|---|---|---|
| ratio of variances | 2.393 | 5.882 | 13.732 |
| raw p-value | 0.114140 | 0.002087 | $1.6e-05$ |
| Holm-adjusted p-value | 0.114140 | 0.004174 | $4.7e-05$ |

Table 34: All pairwise comparisons with RT24GF using the $F$-test for equality of two variances of $\log_{10}$(Imidachloprid) between methods, using Holm's method for adjusting $p$-values.

```
> form <- update( form, log10( . ) ~ .)
> # ... rest as above ...
```

| | RT24GF/RT1 | RT24GF/RT24 | RT24GF/RT48 |
|---|---|---|---|
| ratio of variances | 0.995 | 1.326 | 0.740 |
| raw p-value | 0.992581 | 0.604828 | 0.580927 |
| Holm-adjusted p-value | 1.000000 | 1.000000 | 1.000000 |

Table 35 shows the results for all pairwise comparisons with a control, here the reference level RT24GF, for two-sample $F$-tests for homoscedasticity of Imidachloprid between the groups of combinations of hive and methods. (These two-sample $F$-tests are based on the ratios of the variances which are presented in tab. 29, and they in fact test the hypothesis that the ratio of variances equals 1.) The raw $p$-values are adjusted by Holm's method for multiple testing *across all nine combinations of hives and method-ratios.*

Table 35: Per hive: all pairwise comparisons with RT24GF using the $F$-test for equality of two variances of Imidachloprid between methods, using Holm's method for adjusting $p$-values *across all hives.*

```
> form <- formula( paste( Response, "~", Treatment))
> TMP <- by( WorkData, WorkData[[ Group]], function( X) {
+   X <- droplevels( X)
+   tmp <- sapply( setdiff( lev.of.int, reflev),
+           function( lev) {
+             grps <- X[[ Treatment]] %in% c( lev, reflev)
+             wd <- droplevels( X[ grps, ])
+             wd[[ Treatment]] <- relevel( wd[[ Treatment]], ref = reflev)
+             vt <- var.test( form, data = wd)
+             c( estimate = unname( vt$estimate), p.value = vt$p.value)
+             } )
+   colnames( tmp) <- paste0( reflev, "/", colnames( tmp))
+   tmp
+   } )
> pv <- p.adjust( sapply( TMP, function( x) x[ "p.value",]))
> pva <- as.data.frame( matrix( pv, nrow = length( TMP),
+                               dimnames = list( colnames( TMP[[1]]), names( TMP))))
> pva <- mapply( rbind, unclass( TMP), pva, SIMPLIFY = FALSE)
> pva <- lapply( pva, function( x) {
+                   row.names( x) <- c( "ratio of variances", "raw p-values",
+                                       "Holm-adjusted p-values")
+                   x
+                   })
> pmat <- do.call( "rbind", pva)
> my.latex( pmat, rdec = c( 3, 5, 5), rgroup = names( pva), table.env = FALSE)
```

| | RT24GF/RT1 | RT24GF/RT24 | RT24GF/RT48 |
|---|---|---|---|
| **h1** | | | |
| ratio of variances | 1.359 | 13.529 | 76.788 |
| raw p-values | 0.77360 | 0.02712 | 0.00098 |
| Holm-adjusted p-values | 1.00000 | 0.21694 | 0.00885 |
| **h2** | | | |
| ratio of variances | 2.123 | 1.697 | 2.723 |
| raw p-values | 0.48396 | 0.62094 | 0.35531 |
| Holm-adjusted p-values | 1.00000 | 1.00000 | 1.00000 |
| **h3** | | | |
| ratio of variances | 3.051 | 1.521 | 9.668 |
| raw p-values | 0.30548 | 0.69464 | 0.04943 |
| Holm-adjusted p-values | 1.00000 | 1.00000 | 0.34600 |

**Summary:** After adjusting for multiplicity only the ratio of variances for RT24GF and RT48 in hive 1 differs significantly from 1.

Apparently, the differences in variances between group feeding and other methods as displayed in tab. 33 disappear almost completely if one takes hive membership into account. This indicates that those differences are mainly due to (biological?) between-hive variability, and not so much due to the feeding technique.
Of course, it should be kept in mind, that the sample size in each combination of hive and method was only 5, so that the statistical power of detecting a variance ratio between two methods different from 1 was per se not very high for any single hive. (The necessary Holm-adjustment for multiple testing reduced the power even further.)

#### 2.4.1.2 Comparing locations

**Model fitting, model diagnostics, and statistical inference for the fitted model**

Tab. 36 presents the two-factorial analysis of variance (ANOVA) table of Imidachloprid on method and hive with interaction using sequential tests (aka "type-I tests") for the model terms. For details on structure and interpretation of the ANOVA table see the explanations regarding tab. 3.

Table 36: Sequential ("type-I tests") two-factorial ANOVA of Imidachloprid on method and hive with interaction between method and hive. (`update()` is used here for the same reason as in tab. 1.)

```
>    # 'Building' the model formula using variables (for the sake of flexibility):
> form <- formula( paste( Response, "~", Group, "*", Treatment))
> fit <- aov( form, data = WorkData)   # Fitting the underlying linear model.
> fit <- update( fit, ~ .)
> anova( fit)

Analysis of Variance Table

Response: Imi
            Df  Sum Sq Mean Sq F value    Pr(>F)
hive         2    8.05    4.03  0.4460  0.642779
method       3 1579.22  526.41 58.3175 4.917e-16 ***
hive:method  6  239.61   39.94  4.4242  0.001228 **
Residuals   48  433.27    9.03
---
Signif. codes:  0 '***' 0.001 '**' 0.01 '*' 0.05 '.' 0.1 ' ' 1
```

Table 37: Sequential ("type-I tests") two-factorial ANOVA of $\log_{10}$(Imidachloprid) on method and hive with interaction between method and hive.

```
> anova( fitlog <- update( fit, log10( .) ~ .))

Analysis of Variance Table

Response: log10(Imi)
            Df Sum Sq Mean Sq F value  Pr(>F)
hive         2 0.0227 0.01134  0.4886 0.61649
method       3 4.7485 1.58282 68.1723 < 2e-16 ***
hive:method  6 0.2747 0.04578  1.9717 0.08833 .
Residuals   48 1.1145 0.02322
---
Signif. codes:  0 '***' 0.001 '**' 0.01 '*' 0.05 '.' 0.1 ' ' 1
```

Short formal representation of the models in tab. 36 and 37 is completely analogue to the one in (8) and (9), respectively.

Fig. 26 displays three qualitative diagnostic plots for each of the models (8) and (9): in its left column for the model with Imidachloprid on its original scale, and in its right column for the $\log_{10}$-transformed response (see column titles).

**Diagnostic summary:** (Based on fig. 26.) Neither model indicates a violation of the model assumption of normality, as can be seen in the normal q-q plots for the (studentized) residuals (bottom row). Homoscedasticity of errors is doubtful for the model with untransformed values, though, readable from the plot of residuals vs. fitted values (top row) and from Bartlett's test in tab. 31. In neither model are there any

markedly influential observations (outliers) according to Cook's distances (middle row), albeit they appear a little bit more alike in the log-transformed model.

Comparing the multiple $R^2$-values (goodness-of-fit measure) of 0.81 of the underlying (but not shown) untransformed linear model and 0.82 of the respective log-transformed model (also not shown), both appear to fit almost equally well. In view of this and of the diagnostic summary, we prefer the log-transformed model (further supported by its technical "advantage" of producing only positive response values).

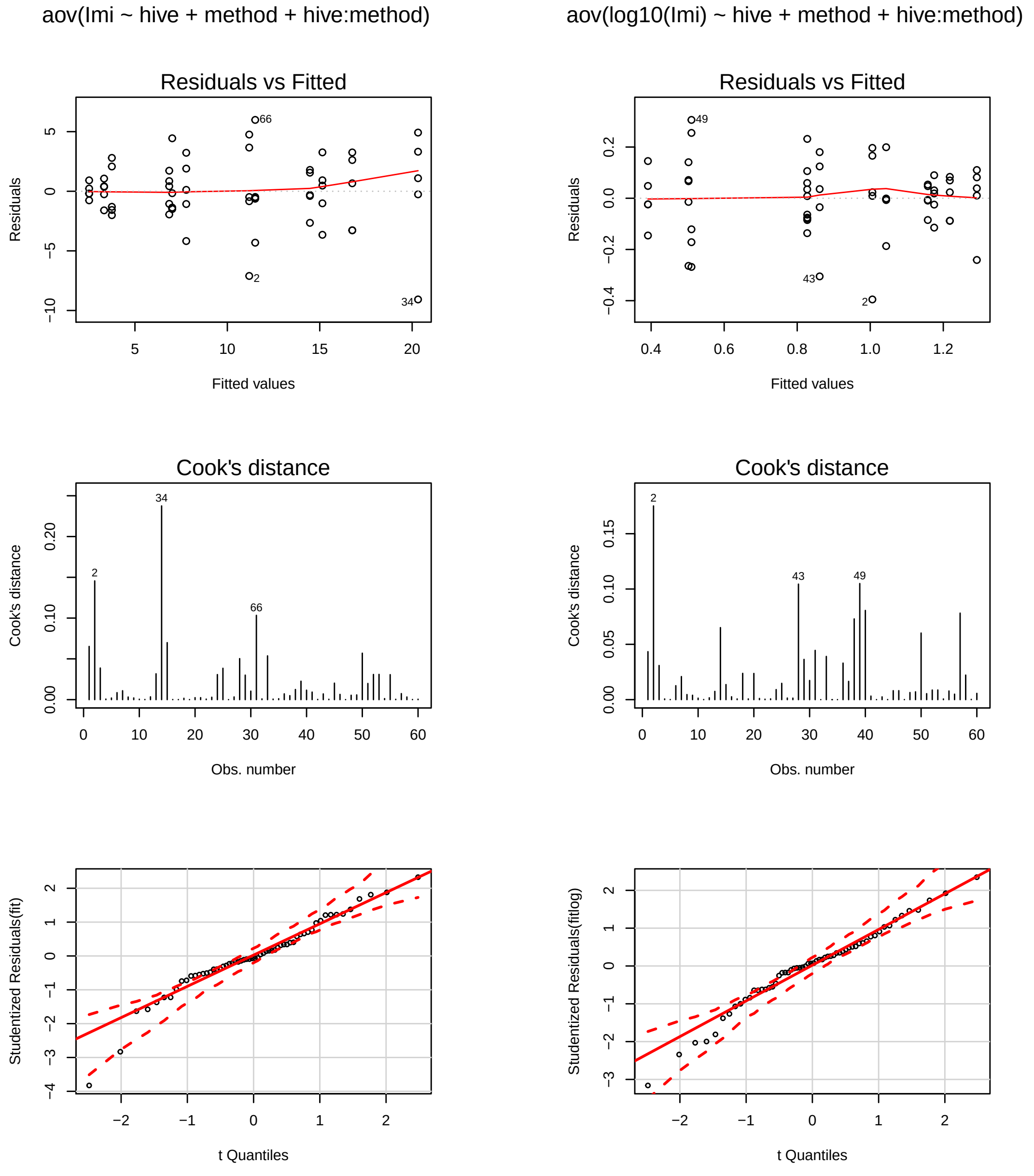


Figure 26: Diagnostic plots for the two-factorial ANOVA models of Imidachloprid on time and hive on the left, and of $\log_{10}$(Imidachloprid) on time and hive on the right. (For a few technical explanations see caption of fig. 4.) (File names: left: *MS-Q4_DiagPlots_Imi.pdf*, right: *MS-Q4_DiagPlots_Imi_B.pdf*)

**ANOVA summary:** (Based on tab. 37.) There is no significant difference ($p = 0.616$) between hives in the overall Imidachloprid-levels (averaged across method). After adjusting for a main effect of hive there is a significant method main effect ($p = 2.51 \times 10^{-17}$). After adjusting for the main effects of hive and method there is no significant interaction effect between hive and method ($p = 8.83 \times 10^{-2}$).

### Additional considerations and visualizations: multiple comparisons

Changing the level order of method so that RT24GF is the first level and hence the reference level in the following analyses. This requires refitting the model under consideration!

```
> WorkData$method <- relevel( WorkData$method, ref = reflev)
> fm <- update( fitlog)
```

Tab. 38 contains the multiple tests for all pairwise comparisons with the control, i.e., the reference level RT24GF (MCC for short), based on the so-called Dunnett contrasts, and tab. 39 presents the pertaining simultaneous confidence intervals. Fig. 27 displays those simultaneous confidence intervals graphically.

*Remark:* The following piece of R-code is for documentation purposes only and shows the technical preparations for the computations underlying tab. 38.

```
> facnames <- rev( names( fm$contrasts))
> CmpFactor <- WorkData[[ facnames[ 1]]]
> GrpFactor <- WorkData[[ facnames[ 2]]]
> levgrid <- expand.grid( levels( CmpFactor), levels( GrpFactor))
> names( levgrid) <- facnames
> X <- model.matrix( formula( fm)[ -2], data = levgrid,
+                    contrasts.arg = fm$contrasts)
> CM <- contrMat( table( CmpFactor), "Dunnett")
> IM <- diag( nlevels( GrpFactor))
> dimnames( IM) <- list( levels( GrpFactor), levels( GrpFactor))
> Kron <- kronecker( IM, CM, make.dimnames = TRUE)
> ContrastMat <- Kron %*% X
```

Table 38: Imidachloprid (on $\log_{10}$-scale!) by method per hive: multiple tests for all pairwise comparisons with the control, i.e., the reference level RT24GF.

```
> set.seed( 20160815)    # To reproduce the simulation-based p-values.
> summary( mcc <- glht( fm, linfct = ContrastMat))

         Simultaneous Tests for General Linear Hypotheses

Fit: aov(formula = log10(Imi) ~ hive + method + hive:method, data = WorkData)

Linear Hypotheses:
                      Estimate Std. Error t value Pr(>|t|)
h1:RT1 - RT24GF == 0  -0.28607    0.09637  -2.968  0.03746 *
h1:RT24 - RT24GF == 0 -0.46448    0.09637  -4.820  0.00013 ***
h1:RT48 - RT24GF == 0 -0.90064    0.09637  -9.346  < 1e-04 ***
h2:RT1 - RT24GF == 0   0.13145    0.09637   1.364  0.76180
h2:RT24 - RT24GF == 0 -0.18215    0.09637  -1.890  0.39306
h2:RT48 - RT24GF == 0 -0.53315    0.09637  -5.532  < 1e-04 ***
h3:RT1 - RT24GF == 0  -0.06034    0.09637  -0.626  0.99713
h3:RT24 - RT24GF == 0 -0.39039    0.09637  -4.051  0.00160 **
h3:RT48 - RT24GF == 0 -0.71557    0.09637  -7.425  < 1e-04 ***
---
Signif. codes:  0 '***' 0.001 '**' 0.01 '*' 0.05 '.' 0.1 ' ' 1
(Adjusted p values reported -- single-step method)
```

Table 39: Imidachloprid (on $\log_{10}$-scale!) by method per hive: simultaneous confidence intervals for all pairwise comparisons with RT24GF.

```
> set.seed( 20160815)    # To reproduce the simulation-based quantile.
> (simci <- confint( mcc))

         Simultaneous Confidence Intervals

Fit: aov(formula = log10(Imi) ~ hive + method + hive:method, data = WorkData)

Quantile = 2.8569
95% family-wise confidence level


Linear Hypotheses:
                       Estimate lwr      upr
h1:RT1 - RT24GF == 0  -0.28607 -0.56139 -0.01076
h1:RT24 - RT24GF == 0 -0.46448 -0.73979 -0.18916
h1:RT48 - RT24GF == 0 -0.90064 -1.17596 -0.62532
h2:RT1 - RT24GF == 0   0.13145 -0.14387  0.40676
h2:RT24 - RT24GF == 0 -0.18215 -0.45747  0.09317
h2:RT48 - RT24GF == 0 -0.53315 -0.80847 -0.25784
h3:RT1 - RT24GF == 0  -0.06034 -0.33566  0.21497
h3:RT24 - RT24GF == 0 -0.39039 -0.66570 -0.11507
h3:RT48 - RT24GF == 0 -0.71557 -0.99088 -0.44025
```

**MCC summary:** Fig. 27 displays the simultaneous confidence intervals for all pairwise comparisons with a control (here: the reference level RT24GF) graphically: Each horizontal line segment that does not intersect the dashed vertical line at zero indicates that the respective difference of $\log_{10}$-concentrations (indicated on the vertical axis on the left) is significantly different from zero. This means approximately, that the *ratio* of Imidachloprid's concentrations of the two pertaining treatments are significantly different from 1 (one) on the "original" scale.

Hence, on a family-wise significance level of 95 %, RT48 and RT24GF are significantly different in all hives, while on the one hand RT24 and RT24GF are significantly different in hives 1 and 3, but not in hive 2, and on the other hand RT1 and RT24GF is only significantly different in hive 1, but not in hives 2 and 3 (with respect to their average $\log_{10}$(Imidachloprid)-values).

```
> par( mar = c( 4, 8.2, 3, 3))
> plot( simci, xlab = bquote( "Difference of"~log[10]~"-concentrations of"
+                             ~.(names( Response))))
```

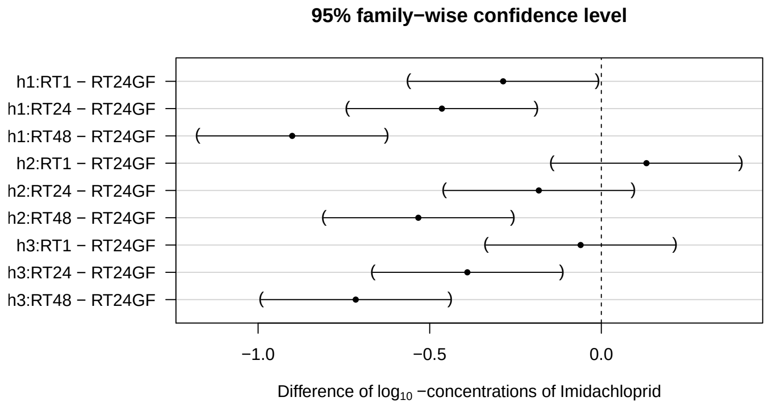


Figure 27: Imidachloprid (on $\log_{10}$-scale!) by method per hive: simultaneous confidence intervals for all pairwise comparisons with RT24GF. (File name: *MS-Q4_AOV_Imi_by_method_and_hive_SimCIplot.pdf*)

### Further considerations and visualizations: MCC for the main effect of method

Tab. 40 shows simultaneous confidence intervals for the interaction parameters of model tab. 37. According to the practical recommendation in [6, Hsu (1996)], p. 183, we consider the models with and without interaction term as practically equivalent since all *but two* of the simultaneous confidence intervals of the interaction effects are not only close to, but in fact contain zero. The two that do not cover zero are anyhow very close to zero (and quite long). Hence, we decide to compute multiple tests for all pairwise comparisons with the control, i.e., the reference level, and the pertaining simultaneous confidence intervals for the main effect of method ignoring the interaction terms.

Table 40: Imidachloprid (on $\log_{10}$-scale!) by method and hive: simultaneous confidence intervals of the interaction parameters of model (9).

```
> oo <- options( contrasts = c( "contr.treatment", "contr.poly"));  fm.up <- update( fm)
> options( oo);    ni <- prod( sapply( fm$xlevels, length) - 1);    cfm <- tail( coef( fm.up), -ni)
> K <- cbind( matrix( 0, ni, length( cfm)), diag( ni));             rownames( K) <- names( cfm)
> set.seed( 20160816);      confint( glht( fm.up, linfct = K))

         Simultaneous Confidence Intervals

Fit: aov(formula = log10(Imi) ~ hive + method + hive:method, data = WorkData)

Quantile = 2.7196
95% family-wise confidence level


Linear Hypotheses:
                       Estimate  lwr       upr
hiveh2:methodRT1 == 0   0.417519  0.046866  0.788172
hiveh3:methodRT1 == 0   0.225731 -0.144922  0.596383
hiveh2:methodRT24 == 0  0.282326 -0.088326  0.652979
hiveh3:methodRT24 == 0  0.074089 -0.296564  0.444742
hiveh2:methodRT48 == 0  0.367486 -0.003167  0.738139
hiveh3:methodRT48 == 0  0.185075 -0.185578  0.555727
```

Tab. 41 contains for the two-way model **without interaction** the multiple tests for all pairwise comparisons with the control (here: the reference level RT24GF), based on the so-called Dunnett contrasts for the main effect of method, and tab. 42 presents the pertaining simultaneous confidence intervals. Fig. 28 displays those simultaneous confidence intervals graphically.

Table 41: Imidachloprid (on $\log_{10}$-scale!) by method and hive: multiple tests for all pairwise comparisons with the control, i.e., the reference level RT24GF.

```
> summary( mcc2 <- glht( fm, linfct = mcp( method = "Dunnett")))

         Simultaneous Tests for General Linear Hypotheses

Multiple Comparisons of Means: Dunnett Contrasts


Fit: aov(formula = log10(Imi) ~ hive + method + hive:method, data = WorkData)

Linear Hypotheses:
                    Estimate Std. Error t value Pr(>|t|)
RT1 - RT24GF == 0  -0.28607    0.09637  -2.968   0.0128 *
RT24 - RT24GF == 0 -0.46448    0.09637  -4.820   <0.001 ***
RT48 - RT24GF == 0 -0.90064    0.09637  -9.346   <0.001 ***
---
Signif. codes:  0 '***' 0.001 '**' 0.01 '*' 0.05 '.' 0.1 ' ' 1
(Adjusted p values reported -- single-step method)
```

Table 42: Imidachloprid (on $\log_{10}$-scale!) by method and hive: simultaneous confidence intervals for all pairwise comparisons with RT24GF **ignoring interaction effects**.

```
> (simci <- confint( mcc2))

         Simultaneous Confidence Intervals

Multiple Comparisons of Means: Dunnett Contrasts


Fit: aov(formula = log10(Imi) ~ hive + method + hive:method, data = WorkData)

Quantile = 2.4244
95% family-wise confidence level


Linear Hypotheses:
                   Estimate lwr      upr
RT1 - RT24GF == 0  -0.28607 -0.51971 -0.05244
RT24 - RT24GF == 0 -0.46448 -0.69811 -0.23084
RT48 - RT24GF == 0 -0.90064 -1.13428 -0.66700


> par( mar = c( 4, 8.2, 3, 3))
> plot( simci, xlab = bquote( "Difference of"~log[10]~"-concentrations of"
+                             ~.(names( Response))),
+       xlim = range( c( 0, simci$confint)))
```

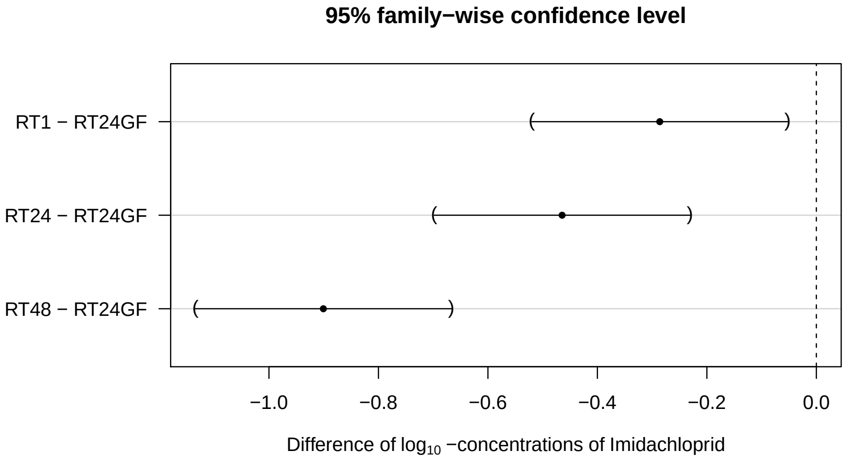


Figure 28: Imidachloprid (on $\log_{10}$-scale!) by method and hive: simultaneous confidence intervals for all pairwise comparisons with RT24GF **ignoring interaction effects**. (File name: *MS-Q4_AOV_Imi_by_method_and_hive_SimCIplot2.pdf*)

**MCC Summary 2:** Fig. 28 displays the simultaneous confidence intervals for all pairwise comparisons with a control (here: the reference level RT24GF) graphically: Each horizontal line segment that does not intersect the dashed vertical line at zero (which is invisible here because it is outside the presented part of the horizontal axis) indicates that the respective difference of $\log_{10}$-concentrations (indicated on the vertical axis on the left) is significantly different from zero. This means approximately, that the *ratio* of Imidachloprid's concentrations of the two pertaining treatments are significantly different from 1 (one) on the "original" scale.

Hence, on a family-wise significance level of 95 %, RT1, R24, and RT48 are all significantly different from RT24GF (with respect to their average $\log_{10}$(Imidachloprid)-values).

Before the end of the report R's original state is regained:

```
> options( startoptions);     rm( startoptions)
```

# 3 Software & References

All graphics and statistical calculations or analyses have been created or made with the "open-source" software R version 3.3.2 (2016-10-31) [1], a programming language and environment for statistical computing and graphics, including the packages:

- **car** (for q-q-plots with confidence intervals), for which [2] is the reference,
- **lattice** (for graphics), see *especially* [3] for reference, and
- **multcomp** (for multiple pairwise comparisons) with [4] as main reference and [5] as even more extensive source.

This report was generated with LaTeX, where [7] as well as [8] are relevant as references for the inclusion of R-code and its results into this report utilizing the R-function Sweave(), and where [9] (for the R-package **Hmisc**) and [10] are references for creating the (few) LaTeX-tables from within R using also Sweave(). This all happened in the "integrated development environment" (IDE) RStudio, Version 0.99.489 [11]. The complete Sweave-files (R- and LaTeX-Code) of this report can be requested by email from the authors.

# References


[1] R Core Team (2016). R: A language and environment for statistical computing. R Foundation for Statistical Computing, Vienna, Austria. `https://www.R-project.org/`.

[2] John Fox and Sanford Weisberg (2011). An R Companion to Applied Regression, Second Edition. Thousand Oaks CA: Sage. `http://socserv.socsci.mcmaster.ca/jfox/Books/Companion`

[3] Sarkar, Deepayan (2008) Lattice: Multivariate Data Visualization with R. Springer, New York. ISBN 978-0-387-75968-5. `http://lmdvr.r-forge.r-project.org`.

[4] Torsten Hothorn, Frank Bretz and Peter Westfall (2008). Simultaneous Inference in General Parametric Models. Biometrical Journal 50(3), 346–363. `http://cran.r-project.org/web/packages/multcomp`.

[5] Bretz, F., Hothorn, T., Westfall, P. (2010). Multiple Comparisons Using R. Chapman & Hall/CRC, Boca Raton/Florida.

[6] Hsu, J. C. (1996): Multiple Comparisons. Theory and methods. Chapman & Hall/CRC, London.

[7] Leisch, F. (2002). Sweave User Manual. `http://www.statistik.lmu.de/~leisch/Sweave/`.

(There exists a much newer version of this manual as *vignette*, which can be "unearthed" by the R-command `vignette("Sweave")`, or at `https://stat.ethz.ch/R-manual/R-devel/library/utils/doc/Sweave.pdf`.)

[8] Leisch, F. (2002). Dynamic generation of statistical reports using literate data analysis. In W. Härdle and B. Rönz, editors, Compstat 2002 - Proceedings in Computational Statistics, pages 575-580. Physika Verlag, Heidelberg, Germany. ISBN 3-7908-1517-9.

[9] Frank E Harrell Jr, with contributions from Charles Dupont and many others. (2015). Hmisc: Harrell Miscellaneous. R package version 3.17-0. `https://CRAN.R-project.org/package=Hmisc`

[10] Whiting, D. (2005). Some examples of conditional typesetting using the latex() function. `http://biostat.mc.vanderbilt.edu/twiki/pub/Main/StatReport/latexFineControl.pdf`

[11] RStudio Team (2015). RStudio: Integrated Development Environment for R. RStudio, Inc., Boston, MA. URL `http://www.rstudio.com/`

# 4 SFig1

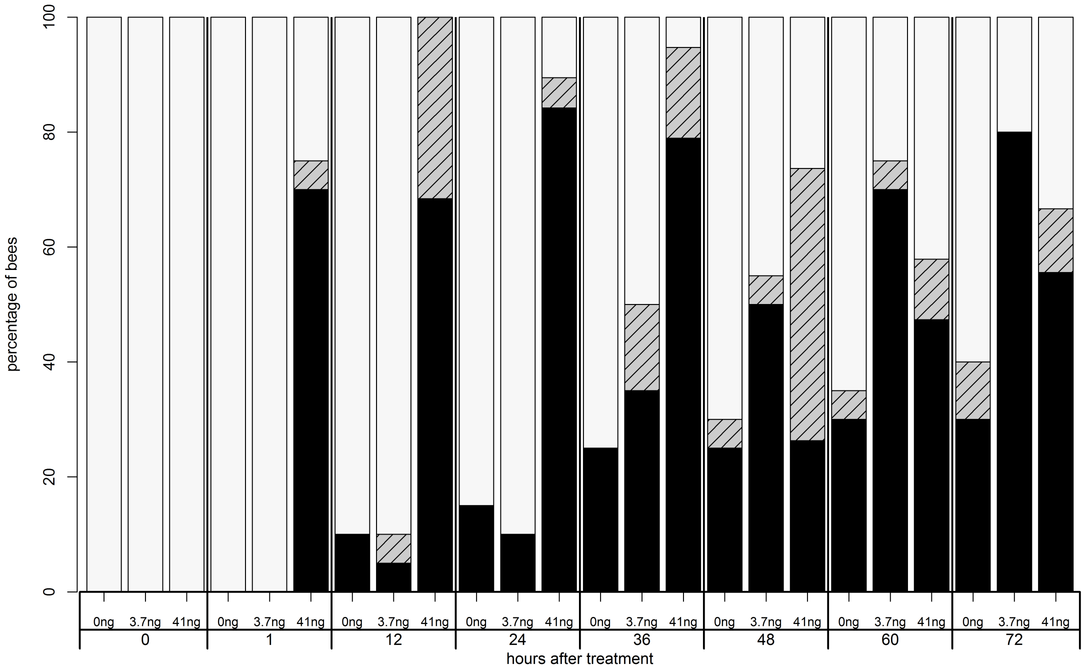


Figure 29: **Supplementary Fig.1**: Dose-dependent effects on locomotory behaviour. Individual honeybees were fed with a single dose of sugar syrup (control) or a dosage of 3.7 and 41 ng/bee imidacloprid (20 bees per treatment group, single experiment). After one, 12, 24, 36, 48, 60, and 72 hours bees were examined for activity. The locomotory behaviour was categorised in (black) immobility, (shaded area) movements of body parts, including ventilation movements of the abdomen, and (white) coordinated activity (e.g. standing, hanging, or walking). After 12 hours, 68% of the bees of the 41 ng treatment group were immobile and 31% only showed movement of body parts. After 48 hours the 41 ng treatment group had the same number of completely immobile bees as the control but the percentage of bees with coordinated movements was lower in this group.
